# Total Synthesis of Kavaratamides A–C and Unnatural Analogs

**DOI:** 10.1021/acsomega.5c02929

**Published:** 2025-06-13

**Authors:** Tomayo I. Berida, Craig W. Lindsley

**Affiliations:** Warren Center for Neuroscience Drug Discovery, Department of Pharmacology, 5718 Vanderbilt University, Nashville, Tennessee 37067, United States

## Abstract

Marine cyanobacteria are a rich source of structurally diverse compounds with biological activity. Herein, we report the total synthesis of kavaratamide A (**1**), a bioactive lipodepsipeptide isolated from *Moorena bouillonii*. The total synthesis of **1** required 16 steps, with a longest linear sequence of 9 steps, and an overall yield of 2.9%. Our convergent synthetic approach led to the efficient synthesis of kavaratamide A–C, C25-*epi-*kavaratamide A, and deoxy-kavaratamide A from a common intermediate providing a blueprint for facilitating rapid structure–activity relationship (SAR) studies.

## Graphical abstract

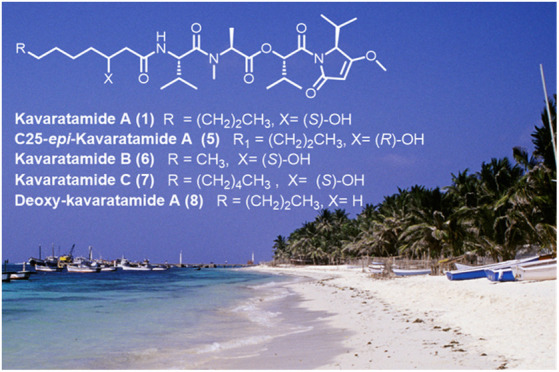

## Introduction

Marine cyanobacteria have garnered significant research interest due to their rich diversity of natural products with potential biological activities. One such cyanobacterium, *Moorena bouillonii*, a filamentous strain collected from Kavaratti, India, is the source of kavaratamide A (**1**) (Figure 1).^1^ Using AI-driven tools such as SMART 2.1 and DeepSAT, along with other structural elucidation methodologies, the Gerwick lab determined that **1** is a lipodepsipeptide containing iheyanone, a rare PKS/NRPS-derived isopropyl-*O*-methylpyrrolidinone moiety (iPr-*O*-Me-pyr). Iheyanone has been reported as a key component of natural products such as iheyamide A (**2**), where it plays a primary role in antiprotozoan activity (Figure 1).^2^ This moiety has also been identified in other peptides, including dysidin (**3**)^3^ while its methyl analog has been found in gallinamide A (**4**) (Figure 1).^4^ Another notable feature of **1** is the unbranched β-hydroxylated fatty acid tail ((*S*)­3-hydroxydecanoic acid (3-HDA)), which imparts lipophilicity to the molecule (Figure 1). Such lipophilic chains are not uncommon in nature and are known to enhance cell permeability in antimicrobials and antiprotozoals.^5–7^ Kavaratamide A (**1**) demonstrates moderate cytotoxic activity against pediatric D283 medulloblastoma cancer, gastric cancer (U251), liver cancer (HepG-2) and pancreatic cancer (PANC-1) cell lines.^1,8,9^ Interestingly, unlike other iheyanone containing natural products, **1** has been shown to be inactive against protozoa.^10^ Given the potential biological significance of **1**, we decided to undertake a total synthesis of this intriguing natural product.^8,9^ During the course of this work, two other groups reported the total synthesis of kavaratamide A (**1**). Ren et al. synthesized **1** and its 5-epimer in 13 steps with overall yields of 0.14 and 0.11%, respectively.^8^ Meanwhile, Sahu et al. reported their synthesis of **1** and its C25-epimer in 18 steps, achieving yields of 3.4 and 4.1%, respectively.^9^

**Figure 1:**
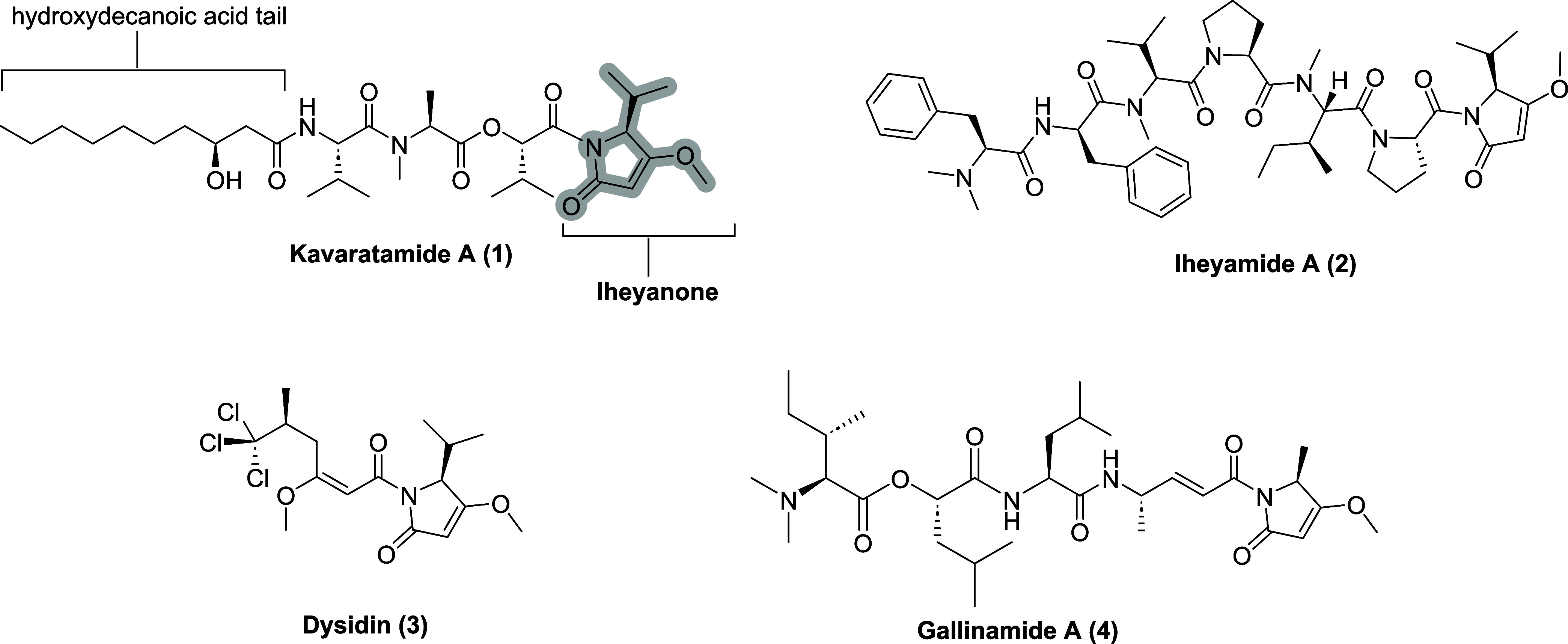
Kavaratamide A and other iheyanone containing natural products.

In this study, we synthesized kavaratamide A (**1**) in 16 steps, with a longest linear sequence (LLS) of 9 steps from commercially available starting materials in 2.9% overall yield utilizing a distinct synthetic strategy.^8,9^ Our convergent synthetic approach enables facile access to analogs for rapid SAR studies and future exploration of biological potential. Therefore, we synthesized C25-*epi-*kavaratamide A (**5**), the C25-epimer of **1** (Figure 2). Furthermore, the Gerwick group proposed the structures for two additional kavaratamide class natural products present in the cyanobacterium extract, kavaratamide B (**6**) and C (**7**) based on “biosynthetic logic” and their MS/MS fragmentation patterns.^1^ Kavaratamides B (**6**) and C (**7**), feature hydroxyhexanoic and hydroxydodecanoic acid tails, respectively. This inspired us to synthesize **6** and **7** through late-stage derivatization (Figure 2). Moreover, it was suggested that the presence of the β-hydroxyl group in **1** could be responsible for its lack of antiprotozoal activity.^1^ Hence, we also report for the first time the synthesis of deoxykavaratamide A (**8**) (Figure 2 **)**.

**Figure 2:**
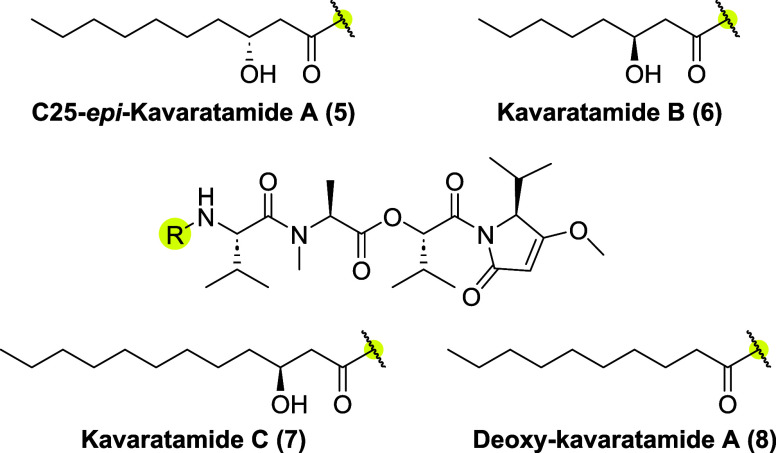
Natural and unnatural analogs of kavaratamide A.

## Results and Discussion

The retrosynthesis of kavaratamide A (**1**) is outlined in Figure 3. To develop a synthetic route that allows for rapid access to diverse analogs to facilitate SAR studies, we divided the synthesis into four segments: iheyanone (**12**), (*S)-*hydroxyisovaleric acid ((*S)*-Hiva, **15**), l-Val-*N*-Me-l-Ala dipeptide fragment (**18**), and (3*S)*-HDA (**22**). We accessed iheyanone (**12**) from Fmoc-protected valine (**9**), using Meldrum’s acid (**10**) as a two-carbon source. We envisioned that asymmetric hydrogenation of commercially available, relatively inexpensive β-keto ester (**23**) would give the desired (3*S)*-HDA (**22**).

**Figure 3:**
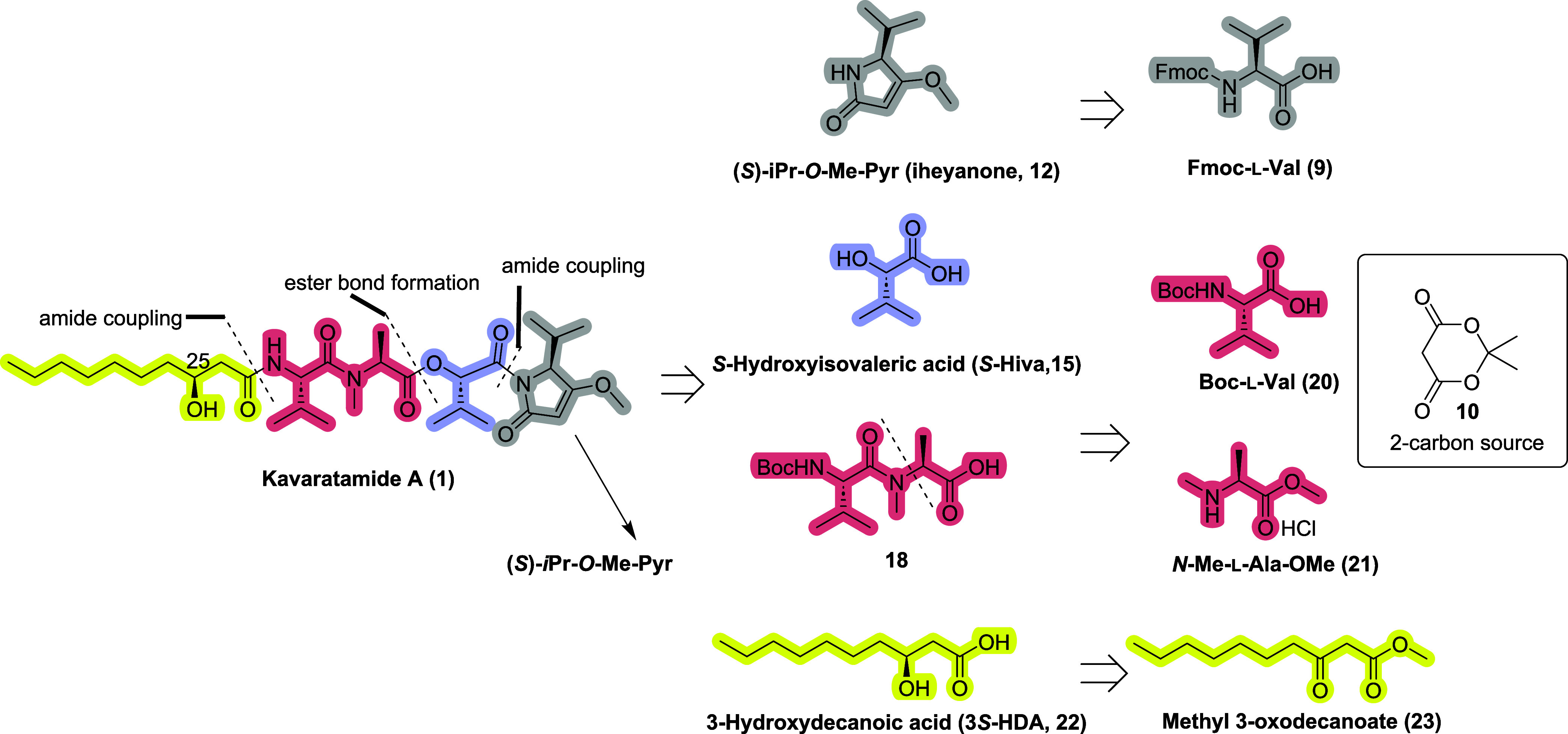
Retrosynthetic analysis of kavaratamide A (**1**).

Iheyanone (**12**) was synthesized in 57% overall yield over four steps, following the previously described procedure (Scheme 1).^4,11^ The two-carbon elongation of Fmoc-protected l-valine (**9**) was achieved using Meldrum’s acid (**10**), followed by decarboxylation and intramolecular cyclization to obtain **11.** Mitsunobu-promoted methylation of the enolic hydroxyl group of **11**, followed by Fmoc-deprotection, yielded iheyanone (**12**). The next step was to synthesize the amidation counterpart pentafluorophenyl ester (**13**). We opted for an activation strategy since classical amide coupling conditions were unsuccessful in our hands, as well as in previous reports.^12^ The activated ester (**13**) was synthesized in three steps from (*S*)-2-hydroxy-3-methylbutanoic acid (**15**). To prevent self-condensation after the formation of the activate ester, TBS protecting group was installed in the α-hydroxy group of **15**. Unsurprisingly, the carboxylic acid was also silylated during the reaction despite the lower temperature employed. However, mild K_2_CO_3_ mediated methanolysis provided **16** which was then used to synthesize the desired product (**13**) with an overall yield of 66% over three steps. Afterward, activated ester (**13**) was condensed with **12** to obtain compound **14**.

**Scheme 1:**
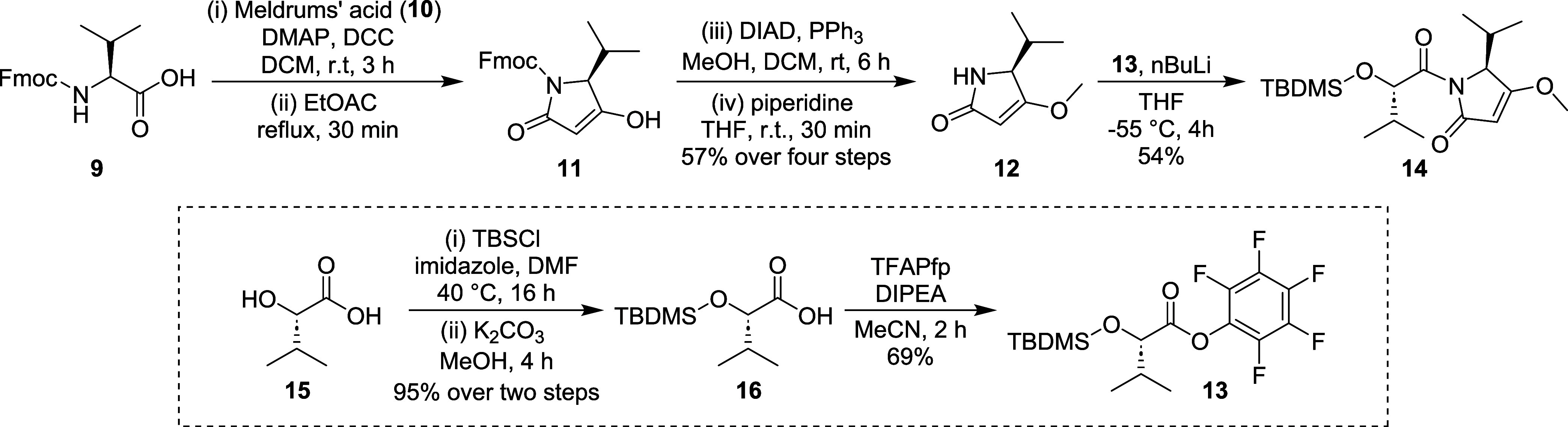
Synthesis of Intermediates **13** and **14**

To complete the assembly of headgroup **19**, which served as the common intermediate for the synthesis of other analogs, we had to esterify alcohol **17** with carboxylic acid **18**. Because decomposition was observed when attempting to desilylate **14** with TBAF, we resorted to deprotection with 4 N HCl in 1,4-dioxane for 30 min to furnish **17**. The alcohol **17** was carried forward without isolation and reacted with dipeptide **18** via Steglich esterification (Scheme 2). Dipeptide (**18**) was synthesized by coupling commercially available amino acids Boc-l-Val (**20**) and *N*-Me-l-Ala-OMe (**21**), followed by LiOH-mediated hydrolysis (Scheme 2). With fragments **17** and **18** in hand, the formation of desipeptide **19** was achieved with EDC·HCI and DMAP in a 40% yield over 2 steps.

**Scheme 2:**
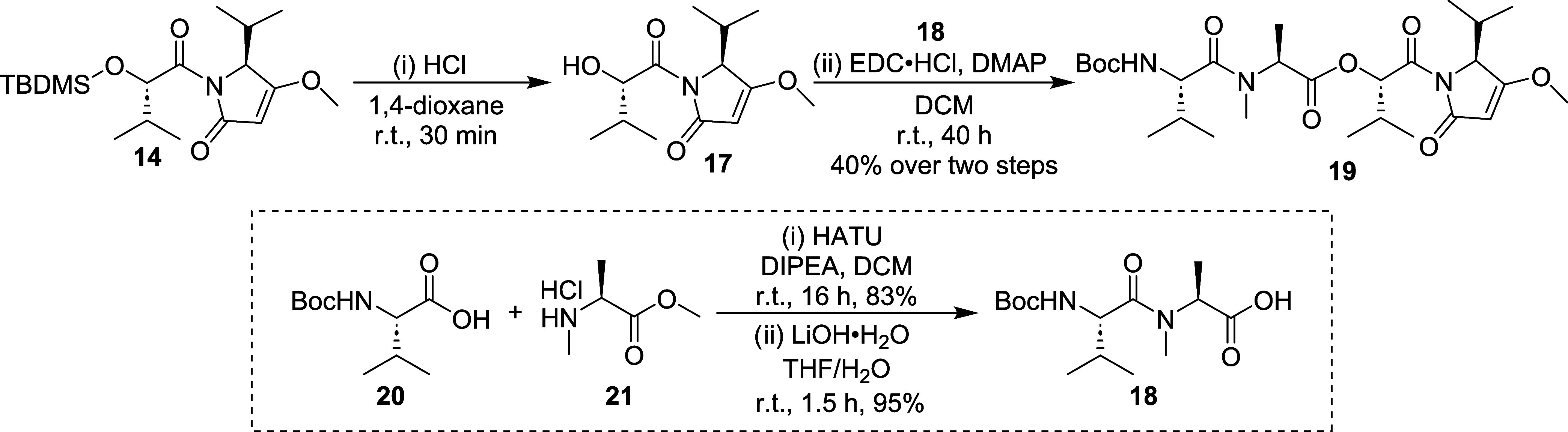
Synthesis of Intermediates **18** and **19**

Several approaches have been employed to enantioselectively access the β-hydroxylated carboxylic acid motif in peptide synthesis.^8,9,13–15^ Ren et al. synthesized (3*S*)-HDA (**22**) in two steps from octanal using a TiCl_4_-mediated Evans-type asymmetric aldol condensation.^8^ However, this approach resulted in a low overall yield of 15% over two steps.^8^ In contrast, Sahu et al. employed a Keck asymmetric allylation approach to obtain (3*S*)-HDA in three steps from octanal.^9^ While the Keck allylation route provides a better yield, the sequence can be labor-intensive and operationally demanding.^9^ In our case, (3*S*)-HDA (**22**) was obtained via asymmetric hydrogenation of β-keto ester using a chiral Ru-(*S*)-BINAP catalyst (Scheme 3).^7,16^ The ruthenium catalyst (Ru-(*S*)-BINAP) was generated *in situ* from (*S*)-BINAP and (COD)­Ru­(2-methylallyl)_2_ for the asymmetric hydrogenation of **23** to give the methyl (*S*)-β-hydroxyester **24**, which was then hydrolyzed to 3*S*-HDA (**22**) using LiOH monohydrate.

**Scheme 3:**
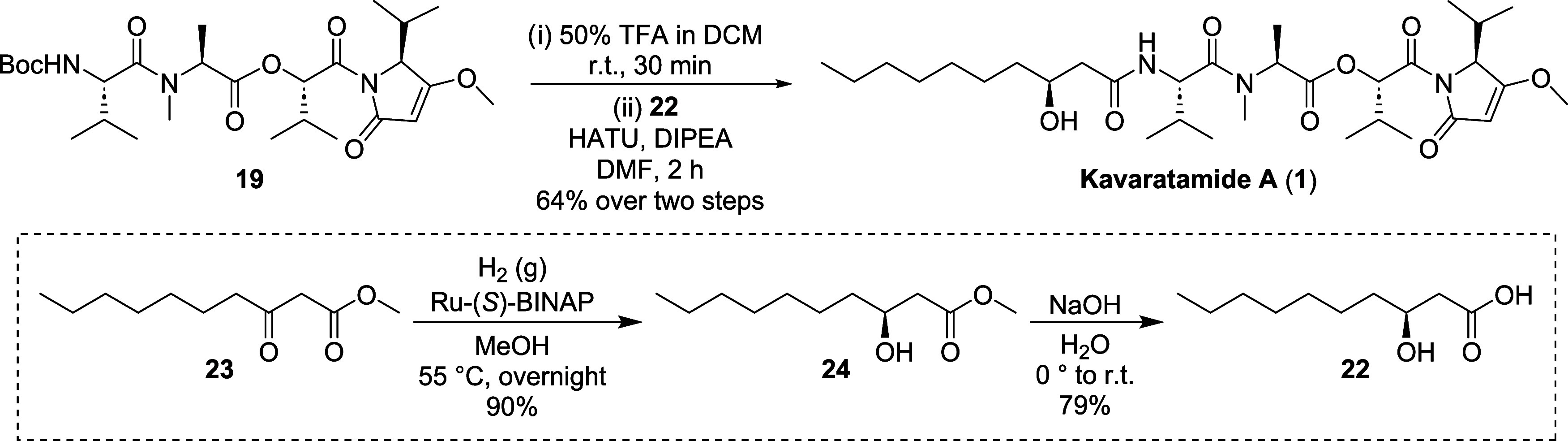
Synthesis of Kavaratamide A and 3-Hydroxydecanoic Acid (**22**)

The final synthetic sequence of **1** commenced with the deprotection of **19** using 50% (v/v) TFA/DCM, after which the TFA salt was coupled with **22** in 64% yield over two steps. Overall, kavaratamide A (**1**) was synthesized over 16 steps (9 LLS) with a total yield of 2.9% starting from commercially available Fmoc-*N*-l-valine (**9**), (*S*)-Hiva acid (**15**), Boc-l-valine (**20**), *N*-Me-l-Ala-OMe (**21**) and methyl 3-oxodecanoate (**23**).

Unnatural analogs of **1** were obtained by reacting the appropriate carboxylic acids (**30**–**32**) with deprotected **19** in a similar fashion delineated for the synthesis of **1** (Scheme 4). To obtain (*R*)-3-hydroxydecanoic acid (3*R*-HDA, **30**), Ru-(*R*)-BINAP was employed in the synthesis of the methyl (*R*)-β-hydroxyester **27.** Analog **8** was synthesized by reacting decanoyl chloride with deprotected **19** (Scheme 4). To our knowledge, this is the first reported synthesis of kavaratamide B (**6**), kavaratamide C (**7**), and deoxy-kavaratamide A (**8**). Variations in the fatty acid chain length of bacterially derived lipodepsipeptides are well-documented to play a role in modulating their biological activity.^1,17–19^ Our strategy enables easy modification of the side chain while maintaining control over stereochemistry at C25. Moreover, other lipophilic or functional groups, besides unbranched β-hydroxylated fatty acids, could also be easily explored.

**Scheme 4:**
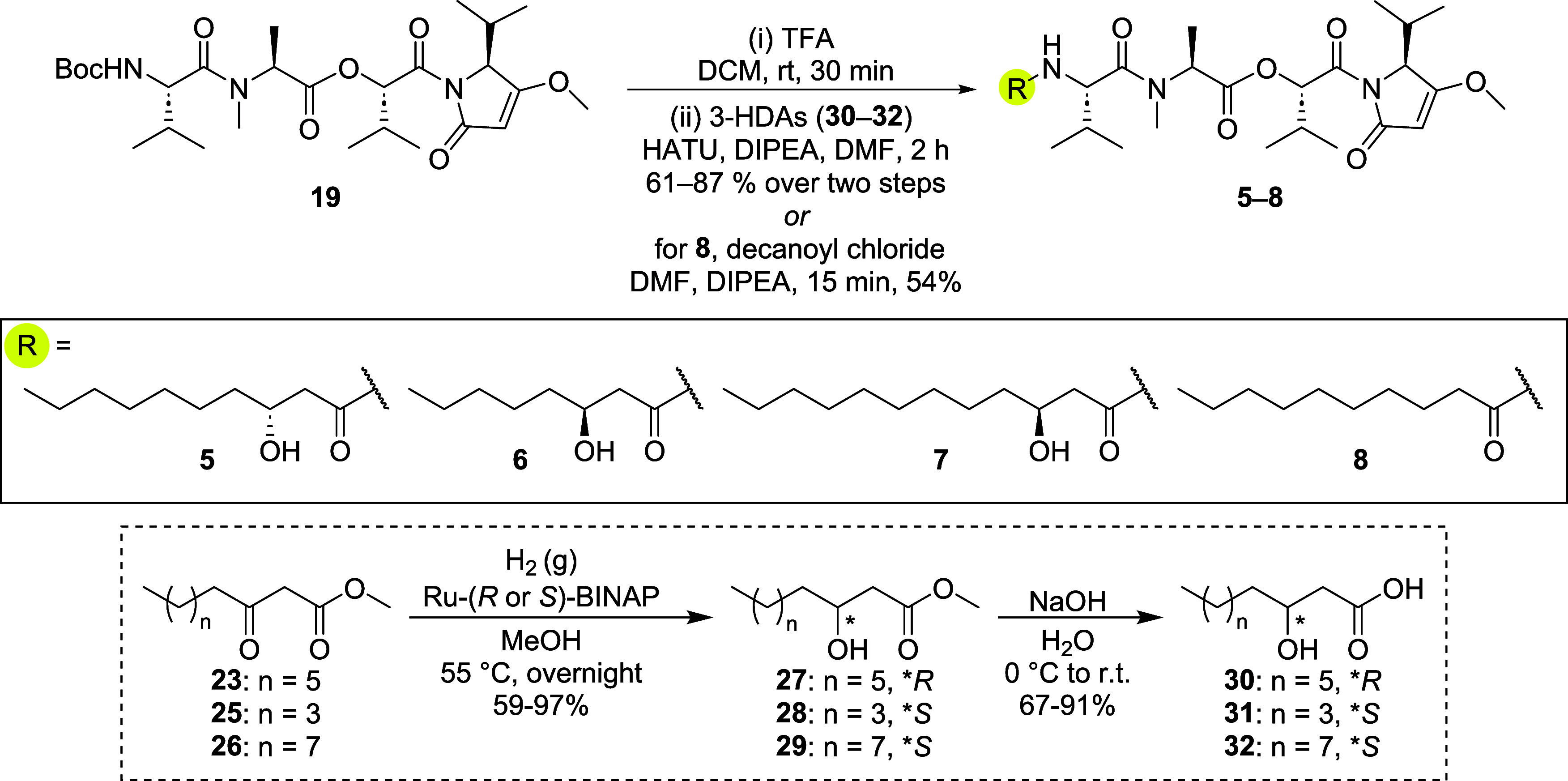
Synthesis of Kavaratamide A Analogs

## Conclusions

In conclusion, we report an efficient synthesis of kavaratamide A (**1**) and four of its analogs: the C25-epimer (**5**), kavaratamide B (**6**), kavaratamide C (**7**), and deox-ykavaratamide A (**8**). Kavaratamide A (**1**) was synthesized in 16 steps (9 LLS) with an overall yield of 2.9% from commercially available Fmoc-*N*-l-valine, *N*-Me-l-Ala-OMe, Boc-l-valine, (*S*)-Hiva acid, and methyl 3-oxodecanoate. Efforts to evaluate the biological activity of these compounds are currently underway, and comprehensive SAR studies will be reported in due course.

## Experimental Section

### General Methods

All reactions were carried out employing standard chemical techniques. Solvents used for reactions and extraction were ACS grade, and HPLC grade solvents were used for purification. All reagents were purchased from commercial sources and were used without further purification.

All NMR spectra were recorded on a 400 MHz Bruker AV-400 instrument. ^1^H chemical shifts are reported as δ values in ppm relative to the residual solvent peak (CDCl_3_ = 7.26). Data are reported as follows: chemical shift, multiplicity (br = broad, s = singlet, d = doublet, t = triplet, q = quartet, p = pentet, dd = doublet of doublets, ddd = doublet of doublet of doublets, td = triplet of doublets, m = multiplet), coupling constant, and integration. ^13^C chemical shifts are reported as δ values in ppm relative to the residual solvent peak (CDCl_3_ = 77.16).

High resolution mass spectra were obtained on an Agilent 6540 UHD Q-TOF with ESI source. MS parameters were as follows: fragmentor: 150, capillary voltage: 3500 V, nebulizer pressure: 60 psig, drying gas flow: 13 L/min, drying gas temperature: 275 °C. Samples were introduced via an Agilent 1290 UHPLC comprised of a G4220A binary pump, G4226A ALS, G1316C TCC, and G4212A DAD with ULD flow cell. UV absorption was observed at 215 and 254 nm with a 4 nm bandwidth. Column: Agilent Zorbax Extend C18, 1.8 μm, 2.1 × 50 mm. Gradient conditions: 5% to 95% CH_3_CN in H_2_O (0.1% formic acid) over 1 min, hold at 95% CH_3_CN for 0.1 min, 0.5 mL/min, 40 °C.

Automated flash column chromatography was performed on Teledyne ISCO CombiFlash system. Optical rotation was obtained on JASCO, Na^+^ P-2000 Series Polarimeter, Cylindrical glass cell 3.5 mm ID x 10 mm. Reverse phase HPLC was performed on a Gilson preparative reverse-phase HPLC system comprised of a 333 aqueous pump with solvent-selection valve, 334 organic pump, GX-271 or GX-281 liquid hander, two column switching valves, and a 155 ultraviolet (UV) detector. UV wavelength for fraction collection was user-defined, with absorbance at 254 nm always monitored. Column: Phenomenex Axia-packed Gemini C18, 30 × 50 mm^2^, 5 μm. Mobile phase: CH_3_CN in H_2_O (0.05% v/v NH_4_OH). Gradient conditions: 0.75 min equilibration, followed by user-defined gradient (25–95%), hold at 95% CH_3_CN in H_2_O (0.05% v/v NH_4_OH) for 1 min, 50 mL/min, 23 °C.
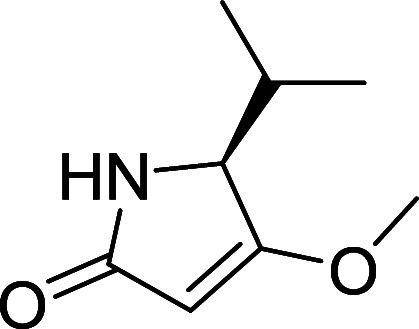


#### Iheyanone (**12**)

Meldrum’s acid (2.12 g, 14.73 mmol), 1,3-dicyclohexylcarbodiimide (2.93 mL, 17.68 mmol), and DMAP (2.38 mL, 17.68 mmol) were added to a solution of Fmoc-l-Val (**9**) (5.00 g, 14.73 mmol) in dry DCM (60 mL). The mixture was stirred at room temperature for 3 h and then filtered. The filtrate was diluted with 40 mL of H_2_O and extracted. Afterward, the combined organic phases were washed sequentially with 0.5 M HCl, H_2_O and brine, dried over anhydrous MgSO_4_, and concentrated under reduced pressure to give a brown oil. The residual oil was refluxed in EtOAc (150 mL) for 30 min. The reaction mixture was concentrated on a rotary evaporator to afford crude residue, (9H-fluoren-9-yl)­methyl (*S*)-3-hydroxy-2-isopropyl-5-oxo-2,5-dihydro-1H-pyrrole-1-carboxylate (**11**) (5.40 g, 14.86 mmol, quantitative yield) as a yellow solid which was carried forward without further purification.

A solution of **11** (5.00 g, 13.67 mmol), triphenylphosphine (3.97 g, 15.13 mmol) and methanol (0.84 mL, 20.64 mmol) in DCM (40 mL) was cooled to 0 °C before diisopropyl azodicarboxylate (4.06 mL, 20.64 mmol) was added dropwise. The reaction mixture was then allowed to warm to room temperature and stirred for 6 h before it was concentrated *in vacuo* to give a colorless oil which was purified by Teledyne ISCO Combi-Flash system (EtOAc/Hex, 0–70%) to afford (9*H*-fluoren-9-yl)­methyl (*S*)-2-isopropyl-3-methoxy-5-oxo-2,5-dihydro-1*H*-pyrrole-1-carboxylate (5.19 g, 13.76 mmol, quantitative yield).

The Fmoc protected iheyanone (5.19 g, 13.76 mmol) was dissolved in THF (15 mL) after which piperidine (1.63 mL, 16.51 mmol) was added slowly and allowed to stir at room temperature for 30 min. The solvent was then removed *in vacuo* to obtain the crude product, which was purified using a Teledyne ISCO Combi-Flash system (0–10% MeOH/DCM) to obtain iheyanone (**12**) (1.20 g, 7.88 mmol, 57% yield over four steps) as an off-white solid. [α]_D_^22^ +3.3 (*c* 1.0, MeOH). ^1^H NMR (400 MHz, CDCl_3_) δ 6.53 (s, 3H), 5.04 (s, 1H), 3.98 (d, *J* = 3.3 Hz, 1H), 3.78 (d, *J* = 1.0 Hz, 3H), 2.13–2.02 (m, 1H), 1.01 (d, *J* = 7.0 Hz, 3H), 0.79 (dd, *J* = 6.8, 0.9 Hz, 3H). ^13^C NMR (101 MHz, CDCl_3_) δ 181.0, 176.8, 92.2, 65.2, 59.5, 29.6, 19.3, 15.6. HRMS (ESI) calculated for C_8_H_13_NO_2_: [M + H]^+^ *m*/*z* = 156.1019, found *m*/*z* = 156.1019. NMR spectra align with previous reports.^2,3^ 
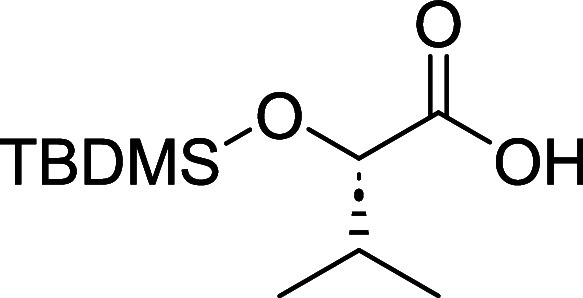


#### (2*S*)-2-[tert-Butyl­(dimethyl)­silyl]­oxy-3-methylbutanoic Acid (**16**)

To a solution of (*S*)-2-hydroxy-3-methylbutanoic acid (**15**) (3.00 g, 25.40 mmol) and tert-butyldimethylchlorosilane (11.81 mL, 63.49 mmol) in DMF (15 mL) was added imidazole (10.07 mL, 152.37 mmol) while stirring at room temperature. The reaction was stirred at 40 °C overnight (16 h) and then partitioned between 100 mL of H_2_O and 100 mL of EtOAc. The organic phase was washed sequentially with 50 mL of saturated citric acid, 50 mL of saturated NaHCO_3_, and 50 mL of brine. The organic phase was then dried over MgSO_4_ and concentrated to afford the crude residue as a viscous colorless oil. To the crude residue dissolved in methanol (25 mL) was added K_2_CO_3_ (3.92 g, 27.94 mmol) in 5 mL of H_2_O slowly. The reaction was stirred at room temperature for 4 h. The mixture’s pH was then adjusted to 4 after which 40 mL of H_2_O was added, and the mixture was extracted with EtOAc (50 mL x 3). The organic phase was washed with 50 mL of H_2_O, and 50 mL of brine, dried over MgSO_4_, filtered and then concentrated to afford a crude residue as viscous colorless oil that was purified by Teledyne ISCO Combi-Flash system (10–60% EtOAc) to obtain **16** (5.60 g, 24.10 mmol, 95% yield) as a colorless oil. [α]_D_^22^ −18.9 (*c* 1.0, MeOH).^20^ ^1^H NMR (400 MHz, CDCl_3_) δ 4.08 (d, *J* = 3.8 Hz, 1H), 2.15–2.02 (m, 1H), 1.12–1.03 (m, 1H), 1.00–0.93 (m, 15H, overlapping signals), 0.11 (d, *J* = 4.4 Hz, 6H). ^13^C NMR (101 MHz, CDCl_3_) δ 175.0, 33.0, 25.8, 18.7, 18.3, 16.8, −4.9, −5.1. Note: compound failed to ionize in both negative and positive modes in HRMS.
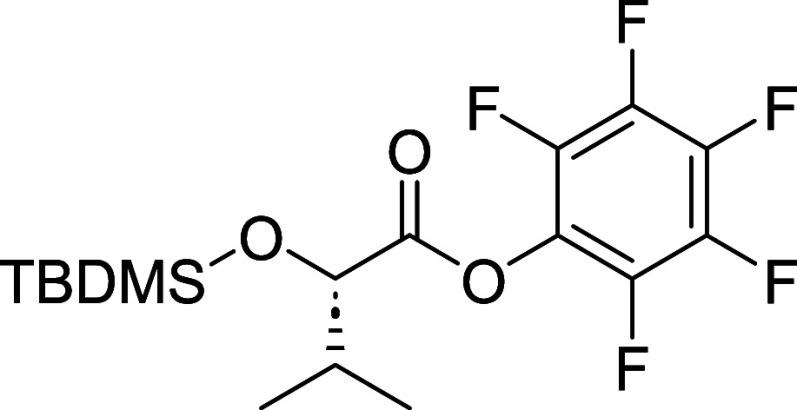


#### (2,3,4,5,6-Pentafluorophenyl) (2*S*)-2-[tert-butyl­(dimethyl)­silyl]­oxy-3-methylbutanoate (**13**)

To compound **16** (3.00 g, 12.91 mmol) dissolved in 20 mL of MeCN and brought to 0 °C was added DIPEA (4.50 mL, 25.82 mmol) and allowed to stir for 10 min. (2,3,4,5,6-pentafluorophenyl) 2,2,2-trifluoroacetate (4.88 mL, 28.4 mmol) was then added to the mixture slowly. The reaction mixture was brought to room temperature and stirred for 2 h. The solvent was then removed under reduced pressure and the crude residue was purified using Teledyne ISCO Combi-Flash system (0–10% EtOAc/Hex,) to afford **13** (2.90 g, 7.30 mmol, 56% yield) as a colorless oil. [α]_D_^22^ −22.5 (*c* 1.0, MeOH). ^1^H NMR (400 MHz, CDCl_3_) δ 4.34 (d, *J* = 4.3 Hz, 1H), 2.30–2.18 (m, 1H), 1.07 (d, *J* = 6.8 Hz, 3H), 1.01 (d, *J* = 6.8 Hz, 3H), 0.94 (s, 9H), 0.11 (d, *J* = 2.1 Hz, 6H). ^13^C NMR (101 MHz, CDCl_3_) δ 169.8, 143.0–142.1 (m), 141.5–140.5 (m), 140.2–139.8 (m), 139.5–139.0 (m), 138.9–137.7 (m), 137.4–136.4 (m), 125.5–124.7 (m), 76.7, 33.3, 25.7, 19.1, 18.4, 16.6, −5.2, −5.3. Note: compound failed to ionize in both negative and positive modes in HRMS.
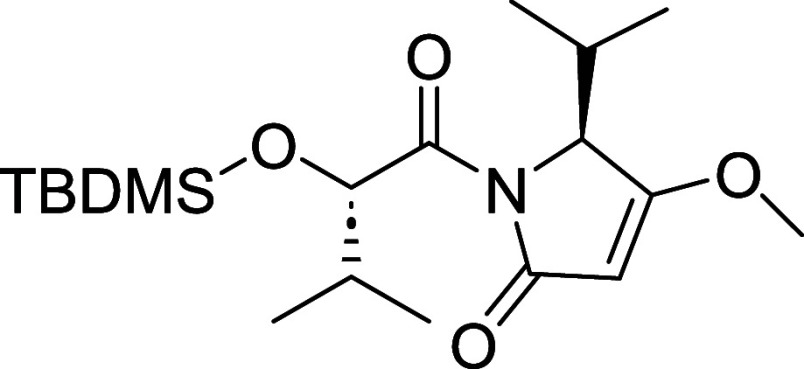


#### (*S*)-1-((*S*)-2-((tert-Butyldimethylsilyl)­oxy)-3-methylbutanoyl)-5-isopropyl-4-methoxy-1,5-dihydro-2H-pyrrol-2-one (**14**)

To iheyanone (**12**) (152 mg, 0.98 mmol) dissolved in THF (2 mL) was added *n*-BuLi (391.52 μL, 0.98 mmol) at −78 °C. The resulting mixture was stirred for 20 min. Compound **13** (260 mg, 0.65 mmol) was dissolved in 1 mL of THF, and added dropwise over 10 min. The reaction mixture was warmed to −55 °C and stirred for 4 h. The reaction was quenched with a saturated solution of NH_4_Cl. The mixture was then extracted with EtOAc x 2. The combined organic phases were washed with H_2_O, brine, dried over MgSO_4_, filtered and concentrated under reduced pressure. The crude residue was then purified using Teledyne ISCO Combi-Flash system (0–30% EtOAc/Hex) to obtain **14** (130 mg, 0.35 mmol, 54% yield) as a white solid. [α]_D_^22^ +44.9 (*c* 1.0, MeOH). ^1^H NMR (400 MHz, CDCl_3_) δ 5.27 (d, *J* = 3.7 Hz, 1H), 5.05 (s, 1H), 4.51 (d, *J* = 2.7 Hz, 1H), 3.84 (s, 3H), 2.68 (pd, *J* = 7.1, 2.7 Hz, 1H), 1.98 (pd, *J* = 6.8, 3.7 Hz, 1H), 1.13 (d, *J* = 7.1 Hz, 3H), 0.98 (d, *J* = 6.8 Hz, 3H), 0.92 (s, 9H), 0.86 (d, *J* = 6.8 Hz, 3H), 0.73 (d, *J* = 7.0 Hz, 3H), 0.05 (s, 3H), 0.02 (s, 3H). ^13^C NMR (101 MHz, CDCl_3_) δ 179.9, 173.5, 170.6, 94.9, 75.9, 64.4, 58.6, 32.0, 28.7, 26.0, 19.8, 19.1, 18.4, 16.0, 15.2, −4.7, −5.1. HRMS (ESI) calculated for C_19_H_35_NO_4_Si: [M + H]^+^ *m*/*z* = 370.2401, found *m*/*z* = 370.2408.
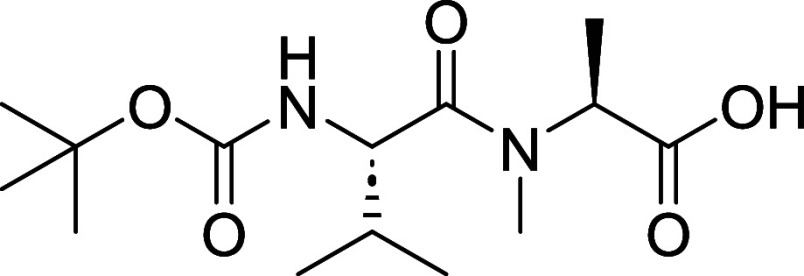


#### *N*-((tert-Butoxycarbonyl)-*L*-valyl)-*N*-methyl-*L*-alanine (**18**)

A solution of *N*-boc-*L*-valine (**20**) (250 mg, 1.15 mmol), HATU (525 mg, 1.38 mmol) and DIPEA (0.60 mL, 3.45 mmol) in DMF (5 mL) was stirred for 10 min before addition of *N*-Me-l-Ala-OMe HCl (**21**) (194 mg, 1.27 mmol). The reaction was stirred at room temperature overnight after which the reaction mixture was diluted with 50 mL of H_2_O and extracted with 25 mL of EtOAc x 2. The combined organic phases were washed with brine, dried over MgSO_4_, filtered and concentrated *in vacuo* to give a residue that was purified by using Teledyne ISCO Combi-Flash system (0–60% EtOAc/Hex) to afford methyl *N*-((tert-butoxycarbonyl)-l-valyl)-*N*-methyl-l-alaninate (**33**) (303 mg, 0.957 mmol, 83% yield) as a colorless viscous liquid. [α]_D_^21^ −51.8 (*c* 1.0, MeOH). ^1^H NMR (400 MHz, CDCl_3_) δ 5.3–5.2 (m, 2H), 4.5 (dd, *J* = 9.3, 5.9 Hz, 1H), 3.7 (s, 3H), 3.0 (s, 3H), 2.1–1.9 (m, 1H), 1.5–1.4 (m, 12H), 1.0 (d, *J* = 6.8 Hz, 3H), 1.0–0.9 (m, 3H). ^13^C NMR (101 MHz, CDCl_3_) δ 172.9, 172.2, 156.1, 55.3, 52.4, 31.5, 31.4, 28.5, 28.4, 19.5, 17.3, 14.3. HRMS (ESI) calculated for C_15_H_28_N_2_O_5_: [M + H]^+^ *m*/*z* = 317.2071, found *m*/*z* = 317.2071.

Methyl ester (**33**) (250 mg, 0.79 mmol) was dissolved in THF (2 mL) and H_2_O (0.2 mL) after which LiOH·H_2_O (17.34 mg, 2.18 mmol) was added. The reaction mixture was stirred at room temperature for 5 h. The reaction mixture was diluted with 1 mL of H_2_O, acidified with 2N aqueous HCl and extracted twice with 25 mL IPA:Chloroform (1:3). The combined organic phases were passed through a phase separator and concentrated to obtain crude residue of **18** (209 mg, 0.69 mmol, 95% yield) which was used in the next step without further purification. [α]_D_^21^ −32.4 (*c* 1.0, MeOH). ^1^H NMR (400 MHz, CDCl_3_) δ 5.34 (d, *J* = 9.3 Hz, 1H), 5.22 (q, *J* = 7.3 Hz, 1H), 4.48 (dd, *J* = 9.4, 6.0 Hz, 1H), 3.06 (s, 3H), 2.01 (dq, *J* = 12.8, 6.3, 5.7 Hz, 1H), 1.44–1.41 (m, 12H), 0.99 (dd, *J* = 6.7, 2.6 Hz, 3H), 0.93–0.88 (m, 3H). ^13^C NMR (101 MHz, CDCl_3_) δ 175.7, 173.4, 156.1, 55.4, 52.5, 31.8, 31.3, 28.5, 28.4, 19.5, 17.4, 14.2. HRMS (ESI) calculated for C_14_H_26_N_2_O_5_: [M + H]^+^ *m*/*z* = 303.1914, found *m*/*z* = 303.1913.
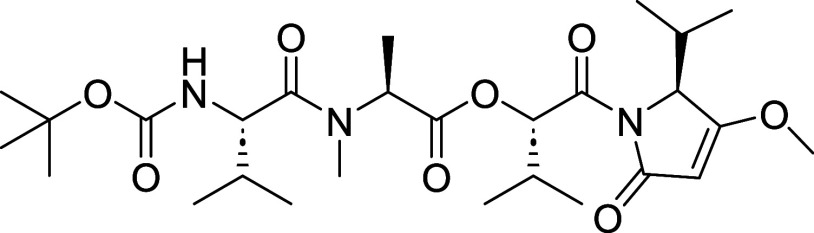


#### (*S*)-1-((S)-2-Isopropyl-3-methoxy-5-oxo-2,5-dihydro-1H-pyrrol-1-yl)-3-methyl-1-oxobutan-2-yl *N*-((tert-butoxycarbonyl)-l-valyl)-*N*-methyl-l-alaninate (**19**)

To (2*S*)-1-[(2*S*)-2-[tert-butyl­(dimethyl)­silyl]­oxy-3-methylbutanoyl]-3-methoxy-2-propan-2-yl-2H-pyrrol-5-one (**14**) (105 mg, 0.28 mmol) was added 1 mL of 4 N HCl in 1,4-dioxane and stirred at room temperature for 30 min. The solvent was removed *in vacuo* to obtain crude residue (**17**) that was carried forward without further purification. Carboxylic acid (**18**), (2*S*)-2-[methyl-[(2*S*)-3-methyl-2-[(2-methylpropan-2-l)­oxycarbonylamino]­butanoyl]­amino]­propanoic acid (**17**) (85.90 mg, 0.28 mmol) and EDC•HCl (65 mg, 0.34 mmol) were added to a flask and allowed to stir for 15 min. DMAP (65 mg, 0.34 mmol) dissolved in 0.50 mL DCM was then added slowly and the reaction mixture stirred for 40 h. The reaction mixture was poured into 10 mL of DCM and washed with H_2_O, brine, dried over MgSO_4_, filtered and concentrated *in vacuo*. The crude residue was purified using a Teledyne ISCO Combi-Flash system (0–40% EtOAc) to obtain **19** as a colorless viscous oil (62 mg, 0.1149 mmol, 40% yield over two steps). [α]_D_^22^ −16.4 (*c* 1.0, MeOH). ^1^H NMR (400 MHz, CDCl_3_) δ 5.80 (d, *J* = 3.1 Hz, 1H), 5.32–5.24 (m, 2H), 5.06 (s, 1H), 4.49 (d, *J* = 2.7 Hz, 1H), 3.84 (s, 3H), 3.02 (s, 3H), 2.63–2.53 (m, 1H), 2.21 (pd, *J* = 6.9, 3.2 Hz, 1H), 1.98 (dt, *J* = 13.0, 6.5 Hz, 1H), 1.79 (s, 1H), 1.47–1.37 (m, 13H), 1.08 (d, *J* = 7.2 Hz, 3H), 1.04 (d, *J* = 6.8 Hz, 3H), 0.99 (d, *J* = 6.8 Hz, 3H), 0.90 (dd, *J* = 11.1, 6.7 Hz, 7H), 0.77 (d, *J* = 6.9 Hz, 3H). ^13^C NMR (101 MHz, CDCl_3_) δ 180.0, 172.5, 171.3, 170.1, 169.2, 156.0, 94.7, 79.5, 78.3, 64.3, 58.7, 55.2, 52.6, 31.5, 28.9, 28.5, 28.4, 19.8, 19.7, 18.8, 17.2, 16.1, 15.3, 14.2. HRMS (ESI) calculated for C_27_H_45_N_3_O_8_: [M + H]^+^ *m*/*z* = 540.3275, found *m*/*z* = 540.3279.
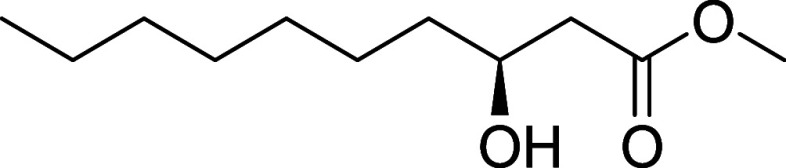


#### Methyl (*S*)-3-Hydroxydecanoate (**24**)

(*S*)-BINAP (16 mg, 0.02 mmol) and (COD)­Ru­(2-methylallyl)_2_ (8 mg, 0.02 mmol) were added to anhydrous acetone (3 mL) in an oven-dried vial and purged with argon. Methanolic HBr (12 μL of 48% HBr diluted in 0.6 mL of anhydrous MeOH) was then added to this suspension and the reaction mixture was stirred at room temperature for 30 min. Thereafter, the solvent was evaporated thoroughly *in vacuo* and the yellow precipitate was used immediately without further purification. A solution of methyl 3-oxodecanoate (**23**) (200 mg, 1.0 mmol) in degassed dry MeOH (4 mL) was added to the vial containing [RuBr_2_((*S*)-BINAP)] ligand with a syringe under argon. The flask was purged with H_2_ and heated at 55 °C under a hydrogen balloon overnight. Next, the reaction mixture was cooled, filtered and concentrated *in vacuo*. The residue was purified by Teledyne ISCO Combi-Flash system (0–40% EtOAc/Hex) to afford **24** (182 mg, 0.90 mmol, 90% yield) as a colorless oil. [α]_D_^22^ +23.5 (*c* 1.0, CHCl_3_).^7^ ^1^H NMR (400 MHz, CDCl_3_) δ 4.06–3.93 (m, 1H), 3.71 (s, 3H), 2.52 (dd, *J* = 16.4, 3.1 Hz, 1H), 2.41 (dd, *J* = 16.4, 9.0 Hz, 1H), 1.57–1.20 (m, 12H), 0.88 (t, *J* = 6.8 Hz, 3H). ^13^C NMR (101 MHz, CDCl_3_) δ 173.7, 68.2, 51.9, 41.2, 36.7, 31.9, 29.6, 29.4, 25.6, 22.8, 14.2. NMR aligns with previous report.^7^ HRMS (ESI) calculated for C_10_H_20_O_3_: [M - H]^−^ *m*/*z* = 187.1340, found *m*/*z* = 187.1348.
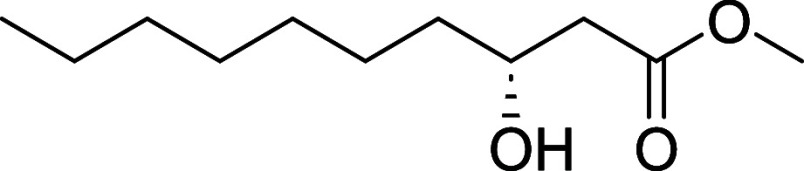


#### Methyl (*R*)-3-Hydroxydecanoate (**27**)

Compound was prepared from **23** (100 mg, 0.50 mmol) as described for compound **24** using an (*R*)-BINAP. Yield = 60 mg, 0.30 mmol, 59%; colorless oil. [α]_D_^22^ −13.5 (*c* 1.0, CHCl_3_).^7^ ^1^H NMR (400 MHz, CDCl_3_) δ 4.06–3.95 (m, 1H), 3.71 (d, *J* = 1.1 Hz, 3H), 2.52 (dd, *J* = 16.4, 3.1 Hz, 1H), 2.41 (dd, *J* = 16.4, 9.0 Hz, 1H), 1.57–1.19 (m, 12H), 0.88 (t, *J* = 7.1 Hz, 3H). ^13^C NMR (101 MHz, CDCl_3_) δ 173.7, 68.2, 51.9, 41.2, 36.7, 31.9, 31.8, 29.6, 29.4, 25.6, 22.8, 14.2. NMR aligns with previous report.^7^ HRMS (ESI) calculated for C_10_H_20_O_3_: [M - H]^−^ *m*/*z* = 187.1340, found *m*/*z* = 187.1348.
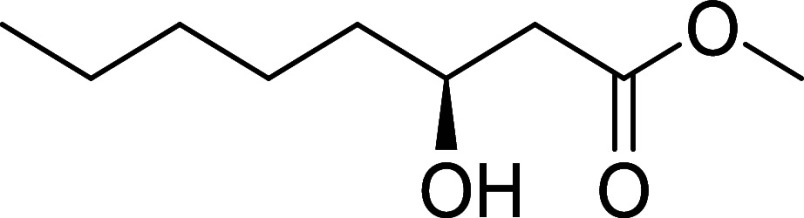


#### Methyl (S)-3-Hydroxyoctanoate (**28**)

Compound was prepared from **25** (200 mg, 0.90 mmol) as described for compound **24**. Yield = 196 mg, 1.12 mmol, 97%; pale yellow oil. [α]_D_^22^ +21.3 (*c* 1.0, CHCl_3_).^21^ ^1^H NMR (400 MHz, CDCl_3_) δ 4.05–3.95 (m, 1H), 3.71 (d, *J* = 0.8 Hz, 3H), 2.51 (dd, *J* = 16.4, 3.1 Hz, 1H), 2.41 (dd, *J* = 16.4, 9.0 Hz, 1H), 1.59–1.21 (m, 8H), 0.89 (t, *J* = 6.7 Hz, 3H). ^13^C NMR (101 MHz, CDCl_3_) δ 173.7, 68.2, 51.9, 41.2, 36.6, 31.9, 25.3, 22.7, 14.2. HRMS (ESI) calculated for C_8_H_16_O_3_: [M - H]^−^ *m*/*z* = 159.1027, found *m*/*z* = 159.1030.
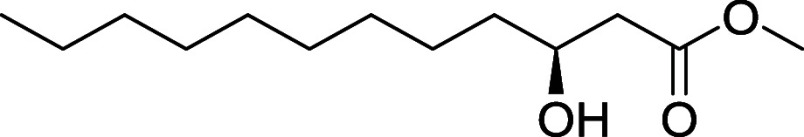


#### Methyl (*S*)-3-Hydroxydodecanoate (**29**)

Compound was prepared from **26** (200 mg, 1.00 mmol) as described for compound **24**. Yield = 152 mg, 0.66 mmol, 75%; colorless oil. [α]_D_^22^ +16.5 (*c* 1.0, CHCl_3_). ^1^H NMR (400 MHz, CDCl_3_) δ 4.05–3.95 (m, 1H), 3.71 (s, 3H), 2.52 (dd, *J* = 16.4, 3.1 Hz, 1H), 2.41 (dd, *J* = 16.4, 9.0 Hz, 1H), 1.58–1.21 (m, 17H), 0.88 (t, *J* = 6.6 Hz, 3H). ^13^C NMR (101 MHz, CDCl_3_) δ 173.7, 68.2, 51.9, 41.2, 36.7, 32.0, 29.7, 29.7, 29.7, 29.5, 25.6, 22.8, 14.3. HRMS (ESI) calculated for C_12_H_24_O_3_: [M - H]^−^ *m*/*z* = 215.1653, found *m*/*z* = 215.1655.
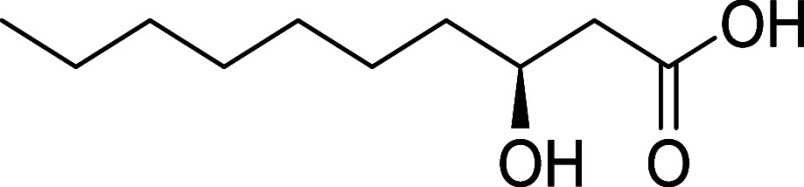


#### (*S*)-3-Hydroxydecanoic Acid (**22**)

1 M aqueous NaOH (2.67 mL, 2.67 mmol) was added to methyl (3*S*)-3-hydroxydecanoate (**24**) (270 mg, 1.33 mmol) at 0 °C. The reaction mixture was warmed to room temperature and stirred for 2 h. The reaction mixture was then acidified with aqueous 2 N HCl dropwise, and pH adjusted to 2–3. The solution was extracted with EtOAc (3 × 10 mL). The combined organic layers were washed with brine, dried over MgSO_4_, filtered and evaporated to give (3*S*)-3-hydroxydecanoic acid (198 mg, 1.05 mmol, 79% yield) as a white solid. [α]_D_^22^ +11.6 (*c* 1.0, CHCl_3_).^7^ ^1^H NMR (400 MHz, CDCl_3_) δ 6.85 (br, 1H), 4.03 (tt, *J* = 8.1, 3.8 Hz, 1H), 2.56 (dd, *J* = 16.5, 3.2 Hz, 1H), 2.46 (dd, *J* = 16.5, 8.9 Hz, 1H), 1.62–1.21 (m, 12H), 0.87 (t, *J* = 7.0, 6.5 Hz, 3H). ^13^C NMR (101 MHz, CDCl_3_) δ 178.1, 68.2, 41.2, 36.6, 31.9, 29.6, 29.3, 25.6, 22.8, 14.2. NMR aligns with previous report.^7^ Note: compound failed to ionize in both negative and positive modes in HRMS.
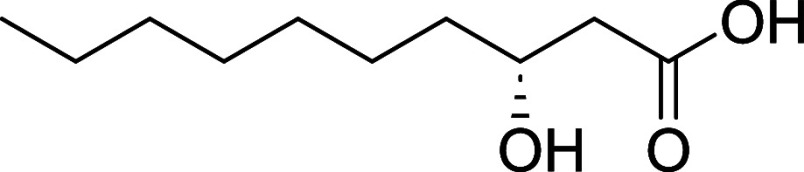


#### (*R*)-3-Hydroxydecanoic Acid (**30**)

Compound was prepared from **27** (50 mg, 0.25 mmol) as described for compound **22**. Yield = 42 mg, 0.23 mmol, 91%. [α]_D_^22^ −22.6 (*c* 1.0, CHCl_3_).^7^ ^1^H NMR (400 MHz, CDCl_3_) δ 4.03 (tt, *J* = 8.0, 3.7 Hz, 1H), 2.58 (dd, *J* = 16.6, 3.2 Hz, 1H), 2.47 (dd, *J* = 16.6, 8.9 Hz, 1H), 1.60–1.22 (m, 12H), 0.88 (t, *J* = 6.8 Hz, 3H). ^13^C NMR (101 MHz, CDCl_3_) δ 177.7, 68.1, 41.1, 36.6, 31.9, 29.6, 29.3, 25.6, 22.7, 14.2. NMR aligns with previous report.^7^ Note: compound failed to ionize in both negative and positive modes in HRMS.
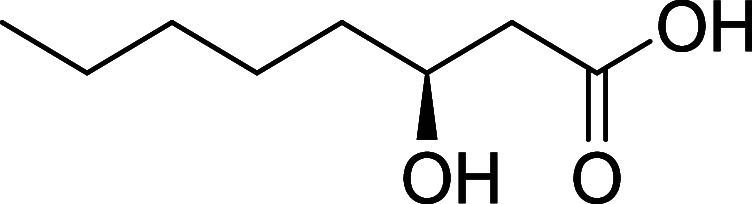


#### (*S*)-3-Hydroxyoctanoic Acid (**31**)

(*S*)-3-hydroxyoctanoic acid was prepared from **28** (57 mg, 0.33 mmol) as described for **22**. Yield = 38 mg, 0.24 mmol, 72%; colorless oil. [α]_D_^22^ +29.3 (*c* 1.0, CHCl_3_).^22^ ^1^H NMR (400 MHz, CDCl_3_) δ 4.09–3.98 (m, 1H), 2.58 (dd, *J* = 16.6, 3.2 Hz, 1H), 2.48 (dd, *J* = 16.6, 8.9 Hz, 1H), 1.60–1.24 (m, 8H), 0.89 (t, *J* = 7.1 Hz, 3H). ^13^C NMR (101 MHz, CDCl_3_) δ 177.6, 68.1, 41.1, 36.6, 31.8, 25.3, 22.7, 14.1. Note: compound failed to ionize in both negative and positive modes in HRMS.
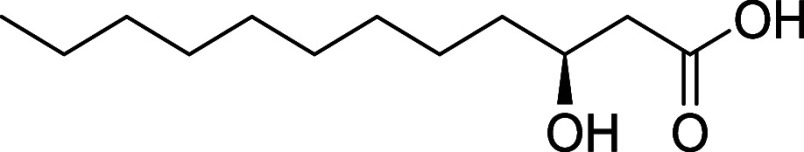


#### (*S*)-3-Hydroxydodecanoic Acid (**32**)

Compound was prepared from **29** (106 mg, 0.46 mmol) as describe for **22**. Yield = 67 mg, 0.31 mmol, 67%; white solid. [α]_D_^22^ +18.7 (*c* 1.0, CHCl_3_).^23^ ^1^H NMR (400 MHz, CDCl_3_) δ 4.03 (tdd, *J* = 8.1, 4.5, 3.0 Hz, 1H), 2.58 (dd, *J* = 16.6, 3.2 Hz, 1H), 2.47 (dd, *J* = 16.6, 8.9 Hz, 1H), 1.61–1.19 (m, 16H), 0.88 (t, *J* = 6.7 Hz, 3H). ^13^C NMR (101 MHz, CDCl_3_) δ 177.8, 68.1, 41.2, 36.6, 32.0, 29.7, 29.7, 29.6, 29.4, 25.6, 22.8, 14.3. Note: compound failed to ionize in both negative and positive modes in HRMS.
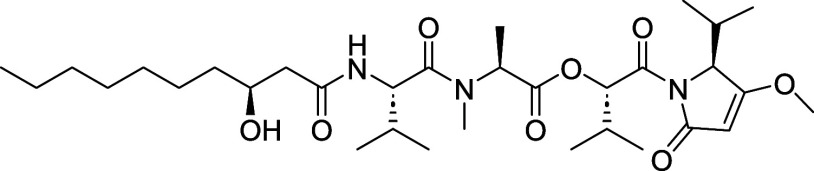


#### Kavaratamide A (**1**)

To compound **19** (10 mg, 0.02 mmol) was added 0.5 mL of 50% (v/v) TFA/DCM and stirred for 30 min. After the deprotection was completed, the solvent was removed *in vacuo* and the crude residue taken to the next step without further purification.

To (3*S*)-3-hydroxydecanoic acid (**22**) (3.5 mg, 0.02 mmol) dissolved in DMF (1 mL) was added HATU (11 mg, 0.03 mmol) and DIPEA (9.68 μL, 0.06 mmol), and the reaction was stirred for 10 min at room temperature. Deprotected intermediate **19** was taken up in 0.2 mL of DMF and added to the reaction mixture. The reaction was then stirred at room temperature for 2 h. The reaction mixture was purified directly on preparative HPCL to afford **1** as an amorphous solid (7.20 mg, 0.012 mmol, 64% yield). [α]_D_^22^ −18.4 (*c* 1.0, MeOH). ^1^H NMR (400 MHz, CDCl_3_) δ 6.44 (d, *J* = 8.9 Hz, 1H), 5.82 (d, *J* = 3.2 Hz, 1H), 5.29 (q, *J* = 7.3 Hz, 1H), 5.07 (s, 1H), 4.84 (dd, *J* = 8.9, 5.5 Hz, 1H), 4.50 (d, *J* = 2.8 Hz, 1H), 3.95 (br. s, 1H), 3.85 (s, 3H), 3.04 (s, 3H), 2.59 (pd, *J* = 7.0, 2.8 Hz, 1H), 2.38 (dd, *J* = 15.0, 2.7 Hz, 1H), 2.33–2.27 (m, 1H), 2.26–2.18 (m, 1H), 2.06 (hept, *J* = 13.0, 6.6 Hz, 1H), 1.54 (dd, *J* = 14.3, 7.7 Hz, 1H), 1.46 (d, *J* = 7.3 Hz, 3H), 1.43–1.39 (m, 1H), 1.27 (d, *J* = 8.5 Hz, 10H), 1.09 (d, *J* = 7.2 Hz, 3H), 1.05 (d, *J* = 6.8 Hz, 3H), 1.01 (d, *J* = 6.8 Hz, 3H), 0.90 (m, 9H), 0.78 (d, *J* = 6.9 Hz, 3H). ^13^C NMR (101 MHz, CDCl3) δ 180.0, 172.9, 172.2, 170.2, 171.2, 169.1, 94.7, 78.4, 69.0, 64.4, 58.7, 53.9, 52.7, 42.9, 37.1, 31.9, 31.9, 31.3, 29.7, 29.4, 28.9, 28.5, 25.6, 22.8, 19.8, 18.8, 17.4, 16.1, 15.3, 14.3, 14.2. HRMS (ESI) calculated for C_32_H_55_N_3_O_8_: [M + Na]^+^ *m*/*z* = 632.3880, found *m*/*z* = 632.3881.
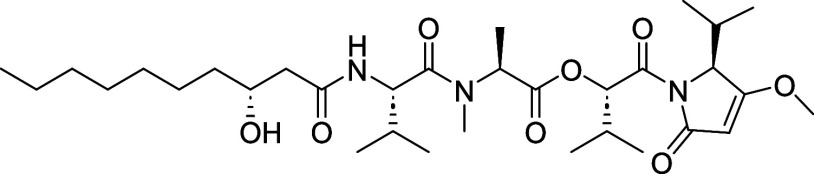


#### C25-*epi*-Kavaratamide A (**5**)

Compound was prepared from **19** (14 mg, 0.03 mmol) and **30** (5 mg, 0.03 mmol) as described for **1**. Yield = 9.90 mg, 0.016 mmol, 63% as a white solid. [α]_D_^22^ −32.6 (*c* 1.0, MeOH). ^1^H NMR (400 MHz, CDCl_3_) δ 6.43 (d, *J* = 9.0 Hz, 1H), 5.82 (d, *J* = 3.2 Hz, 1H), 5.27 (q, *J* = 7.2 Hz, 1H), 5.07 (s, 1H), 4.88 (dd, *J* = 8.9, 5.6 Hz, 1H), 4.50 (d, *J* = 2.8 Hz, 1H), 3.95 (br. s, 1H), 3.85 (s, 3H), 3.05 (s, 3H), 2.59 (pd, *J* = 7.1, 2.9 Hz, 1H), 2.41 (dd, *J* = 15.3, 2.8 Hz, 1H), 2.30 (dd, *J* = 15.4, 8.8 Hz, 1H), 2.26–2.18 (m, 1H), 2.12–1.99 (m, 1H), 1.58–1.48 (m, 1H), 1.45 (d, *J* = 7.3 Hz, 3H), 1.43–1.39 (m, 1H), 1.33–1.21 (m, 10H), 1.09 (d, *J* = 7.2 Hz, 3H), 1.05 (d, *J* = 6.9 Hz, 3H), 1.00 (d, *J* = 6.8 Hz, 3H), 0.94–0.84 (m, 9H), 0.78 (d, *J* = 6.9 Hz, 3H). ^13^C NMR (101 MHz, CDCl_3_) δ 180.0, 172.7, 172.0, 171.1, 170.2, 169.1, 94.7, 78.4, 68.7, 64.4, 58.7, 53.6, 52.8, 42.4, 36.9, 32.0, 31.9, 31.5, 29.7, 29.4, 28.9, 28.5, 25.7, 22.8, 19.8, 19.7, 18.8, 17.4, 16.1, 15.3, 14.3, 14.2. HRMS (ESI) calculated for C_32_H_55_N_3_O_8_: [M + H]^+^ *m*/*z* = 610.4060, found *m*/*z* = 610.4062.
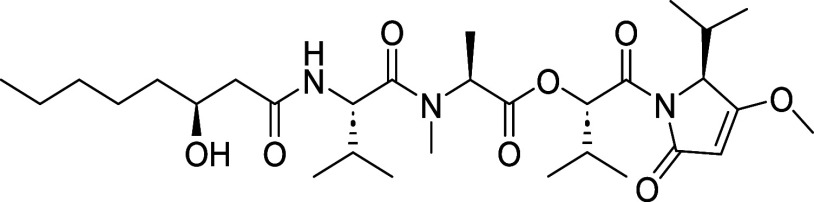


#### Kavaratamide B (**6**)

Compound was prepared from **19** (14 mg, 0.03 mmol) and **31** (4.20 mg, 0.03 mmol) as described for **1**. Yield = 13.30 mg, 0.0229 mmol, 87%; white solid. [α]_D_^22^ −32.6 (*c* 1.0, MeOH). ^1^H NMR (400 MHz, CDCl_3_) δ 6.45 (d, *J* = 8.9 Hz, 1H), 5.82 (d, *J* = 3.2 Hz, 1H), 5.29 (q, *J* = 7.2 Hz, 1H), 5.07 (s, 1H), 4.83 (dd, *J* = 8.9, 5.5 Hz, 1H), 4.50 (d, *J* = 2.8 Hz, 1H), 3.95 (br. s, 1H), 3.85 (s, 3H), 3.04 (s, 3H), 2.64–2.53 (m, 1H), 2.38 (dd, *J* = 15.0, 2.7 Hz, 1H), 2.33–2.27 (m, 1H), 2.25 (s, 1H), 2.12–1.99 (m, 1H), 1.57–1.51 (m, 1H), 1.45 (d, *J* = 7.3 Hz, 3H), 1.41 (d, *J* = 4.0 Hz, 1H), 1.38–1.22 (m, 6H), 1.09 (d, *J* = 7.2 Hz, 3H), 1.05 (d, *J* = 6.8 Hz, 3H), 1.01 (d, *J* = 6.8 Hz, 3H), 0.95–0.85 (m, 9H), 0.78 (d, *J* = 6.9 Hz, 3H). ^13^C NMR (101 MHz, CDCl_3_) δ 180.0, 172.9, 172.2, 171.1, 170.1, 169.1, 94.7, 78.4, 69.0, 64.4, 58.7, 53.9, 52.7, 42.9, 37.1, 31.9, 31.9, 31.3, 28.9, 28.5, 25.3, 22.7, 19.8, 19.8, 18.8, 17.4, 16.1, 15.3, 14.3, 14.2. HRMS (ESI) calculated for C_30_H_51_N_3_O_8_: [M + H]^+^ *m*/*z* = 582.3749, found *m*/*z* = 582.3749.
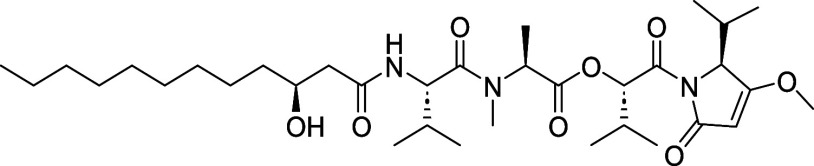


#### Kavaratamide C (**7**)

Compound was prepared from **19** (14 mg, 0.03 mmol) and **32** (5 mg, 0.03 mmol) as described for **1**. Yield = 11.8 mg, 0.018 mmol, 71%; white solid. [α]_D_^21^ −16.5 (*c* 1.0, MeOH). ^1^H NMR (400 MHz, CDCl_3_) δ 6.45 (d, *J* = 8.9 Hz, 1H), 5.82 (d, *J* = 3.2 Hz, 1H), 5.29 (q, *J* = 7.2 Hz, 1H), 5.07 (s, 1H), 4.84 (dd, *J* = 8.9, 5.5 Hz, 1H), 4.50 (d, *J* = 2.8 Hz, 1H), 3.95 (br. s, 1H), 3.85 (s, 3H), 3.04 (s, 3H), 2.38 (dd, *J* = 15.0, 2.7 Hz, 1H), 2.30 (d, *J* = 9.3 Hz, 1H), 2.27–2.19 (m, 1H), 2.13–1.98 (m, 1H), 1.54 (dd, *J* = 14.4, 7.9 Hz, 1H), 1.45 (d, *J* = 7.3 Hz, 3H), 1.41 (s, 1H), 1.34–1.21 (m, 14H), 1.09 (d, *J* = 7.2 Hz, 3H), 1.05 (d, *J* = 6.9 Hz, 3H), 1.01 (d, *J* = 6.8 Hz, 3H), 0.95–0.84 (m, 9H), 0.78 (d, *J* = 6.9 Hz, 3H). ^13^C NMR (101 MHz, CDCl_3_) δ 180.0, 173.0, 172.2, 171.1, 170.1, 169.1, 94.7, 78.4, 69.0, 64.4, 58.7, 53.9, 52.8, 42.9, 37.1, 32.0, 31.9, 31.3, 29.7, 29.7, 29.5, 28.9, 28.5, 25.6, 22.8, 19.8, 19.8, 18.8, 17.4, 16.1, 15.3, 14.3. HRMS (ESI) calculated for C_34_H_59_N_3_O_8_: [M + H]^+^ *m*/*z* = 638.4375, found *m*/*z* = 638.4373.
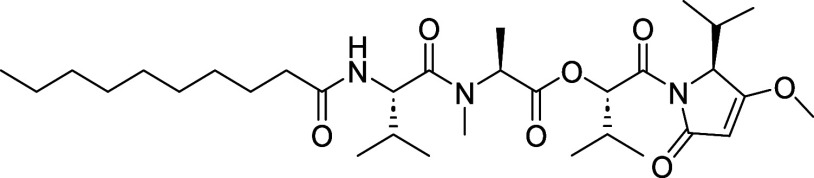


#### Deoxy-Kavaratamide A (**8**)

To **19** (15 mg, 0.03 mmol) was added 0.5 mL of 50% (v/v) TFA/DCM and stirred for 1 h. After the deprotection was completed, the solvent was removed *in vacuo* and the crude residue taken to the next step without further purification.

Deprotected **19** and DIPEA (9.68 mL, 0.06 mmol) were dissolved in DMF (0.5 mL) and cooled to 0 °C. Decanoyl chloride (0.01 mL, 0.03 mmol) was dissolved in DMF (0.5 mL) and then added slowly to the reaction mixture. After that, the reaction was warmed to room temperature and left to stir for 15 min. The reaction mixture was directly loaded onto preparative HPCL to afford **8** (9 mg, 0.0152 mmol, 55% yield) as white solid. [α]_D_^21^ −26.9 (*c* 1.0, MeOH). ^1^H NMR (400 MHz, CDCl_3_) δ 6.18 (d, *J* = 8.9 Hz, 1H), 5.82 (d, *J* = 3.1 Hz, 1H), 5.27 (q, *J* = 7.3 Hz, 1H), 5.07 (s, 1H), 4.87 (dd, *J* = 8.9, 5.8 Hz, 1H), 4.50 (d, *J* = 2.8 Hz, 1H), 3.85 (s, 3H), 3.05 (s, 3H), 2.59 (pd, *J* = 7.1, 2.8 Hz, 1H), 2.20 (t, *J* = 7.7 Hz, 2H), 2.04 (dq, *J* = 13.2, 6.6 Hz, 1H), 1.69–1.59 (m, 2H), 1.44 (d, *J* = 7.2 Hz, 3H), 1.27 (d, *J* = 13.9 Hz, 12H), 1.09 (d, *J* = 7.2 Hz, 3H), 1.05 (d, *J* = 6.9 Hz, 3H), 0.99 (d, *J* = 6.7 Hz, 3H), 0.96–0.84 (m, 9H), 0.78 (d, *J* = 6.9 Hz, 3H). ^13^C NMR (101 MHz, CDCl_3_) δ 180.0, 173.2, 172.3, 171.2, 170.1, 169.2, 94.7, 78.4, 64.4, 58.7, 53.5, 52.7, 37.0, 32.0, 32.0, 31.6, 29.6, 29.5, 29.4, 29.4, 28.9, 28.5, 25.9, 22.8, 19.8, 19.7, 18.8, 17.5, 16.1, 15.3, 14.3. HRMS (ESI) calculated for C_32_H_55_N_3_O_7_: [M + H]^+^ *m*/*z* = 594.4113, found *m*/*z* = 594.4111.

## Supplementary Material



## Notes

NMR spectra of synthesized compounds (^1^H and ^13^C NMR) along with additional chemical procedures (PDF)
