# Supplementary material for: Total Synthesis of Kavaratamides A–C and Unnatural Analogs
Source: ACS Omega. 2025 Jun 13;10(24):26052–60. doi: 10.1021/acsomega.5c02929 (PMC12199036; doi:10.1021/acsomega.5c02929)
Supplement: Supplementary file 1 [file ao5c02929_si_001.pdf]

Supporting Information

Total Synthesis of Kavaratamide A–C and Unnatural Analogs

Tomayo I. Berida[1], Craig W. Lindsey[1*].

[1]*Warren Center for Neuroscience Drug Discovery, Department of Pharmacology, Vanderbilt University, Nashville, Tennessee 37067, Unites States*

**Corresponding authors*

Table of Content

General procedure

Synthesis of β-keto ester (**23**, **25** and **26**) S3

^{1}H and ^{13}C NMR spectra of kavaratamide A, its analogs and intermediates S5

Referencies S28

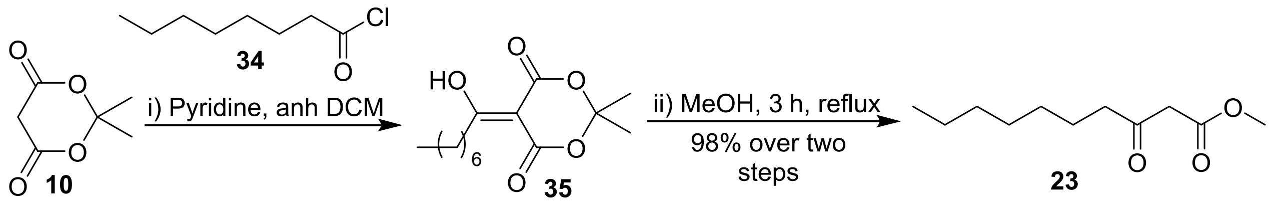

Methyl 3-oxodecanoate (23) Methyl 3-oxodecanoate was prepared as previously described.[1] Dry pyridine (1.99 mL, 24.59 mmol) was added Meldrum's acid (**10**) (1.06 g, 7.38 mmol) in dry DCM (5 mL) at 0°C under argon atmosphere. A solution of octanoyl chloride (**34**) (1.05 mL, 6.15 mmol) dissolved in dry DCM (1 mL) was added dropwise. The cooling bath was removed and the reaction mixture stirred for 3 h. The reaction mixture was then washed with 2M HCl (2 x 10 mL). The aqueous phase was extracted with DCM (2 x 10 mL) and the combined organic layers were washed with 2M HCl (2 x 10 mL) and brine (20 mL) and dried to give acylated Meldrum's acid (**35**) as brown oil. The acylated Meldrum's acid (**35**) (1.6 g, 6.16 mmol) was dissolved in dry MeOH (10 mL) and refluxed overnight. The solvent was removed under reduced pressure and the residue was purified using Teledyne ISCO Combi-Flash system (0–40% EtOAc/Hex). Yield = 1.2 g, 5.99 mmol, 97 %; pale yellow oil. ^{1}H NMR* (400 MHz, $CDCl_3$) ^{1}H NMR (400 MHz, $CDCl_3$) δ 3.74 (s, 3H), 3.45 (s, 2H), 2.53 (t, *J* = 7.4 Hz, 2H), 1.64–1.51 (m, 3H), 1.28 (dt, *J* = 8.2, 5.0 Hz, 8H), 0.88 (t, *J* = 7.2 Hz, 3H). ^{13}C NMR (101 MHz, $CDCl_3$) δ 203.0, 167.9, 52.5, 49.2, 43.2, 31.8, 29.1, 29.1, 23.6, 22.7, 14.2. HRMS (ESI) calculated for $C_{11}H_{20}O_3$: $[M + H]^+$ m/z = 201.1485, found m/z = 201.1485. *Note: NMR peak of keto tautomer are reported here.

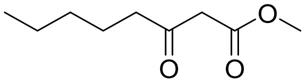

Methyl 3-oxooctanoate (25). Compound was prepared from hexanoyl chloride (1.04 mL, 7.43 mmol) as described for compound **23.** Yield = 1.12 g, 6.50 mmol, 88 %; clear oil. ^{1}H NMR (400 MHz, $CDCl_3$) δ 3.73 (s, 3H), 3.44 (s, 2H), 2.52 (t, *J* = 7.4 Hz, 2H), 1.65–1.52 (m, 2H), 1.29 (dqd, *J* = 16.6, 8.6, 3.6 Hz, 4H), 0.88 (t, *J* = 6.9 Hz, 3H). ^{13}C NMR (101 MHz, $CDCl_3$) δ 203.0, 167.8, 52.4, 49.1, 43.1, 31.3, 23.3, 22.5, 14.0. HRMS (ESI) calculated for $C_9H_{16}O_3$: $[M + H]^+$ m/z = 173.1172, found m/z = 173.1172

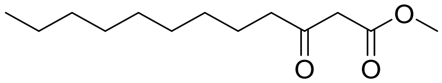

Methyl 3-oxododecanoate (26). Compound was prepared from of decanoyl chloride (1.09 mL, 5.24 mmol) as described for compound **23**. Yield = 0.73 g, 3.20 mmol, 61%; clear oil. ^{1}H NMR (400 MHz, $CDCl_3$) δ 3.72 (s, 3H), 3.43 (s, 2H), 2.51 (t, *J* = 7.4 Hz, 2H), 1.57 (p, *J* = 7.2 Hz, 2H), 1.26 (dd, *J* = 9.9, 5.1 Hz, 12H), 0.86 (t, *J* = 6.8 Hz, 3H). ^{13}C NMR (101 MHz, $CDCl_3$) δ 203.0, 167.8, 52.5, 49.2, 32.0, 29.5, 29.5, 29.4, 29.1, 23.6, 22.8, 14.2. HRMS (ESI) calculated for $C_9H_{16}O_3$: $[M + H]^+$ m/z = 229.1798, found m/z = 229.1798

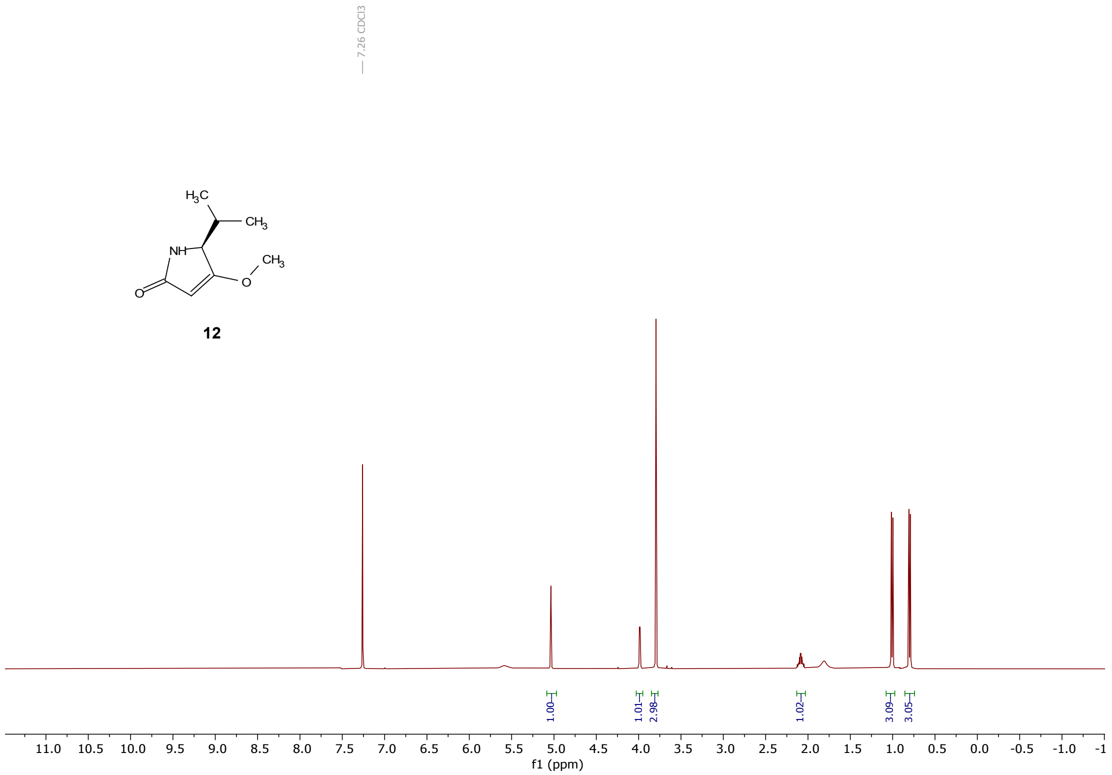
7.26 CDCl3
H3C
CH3
NH
CH3
O
O
12
1.00
1.01
2.98
1.02
3.09
3.05
f1 (ppm)

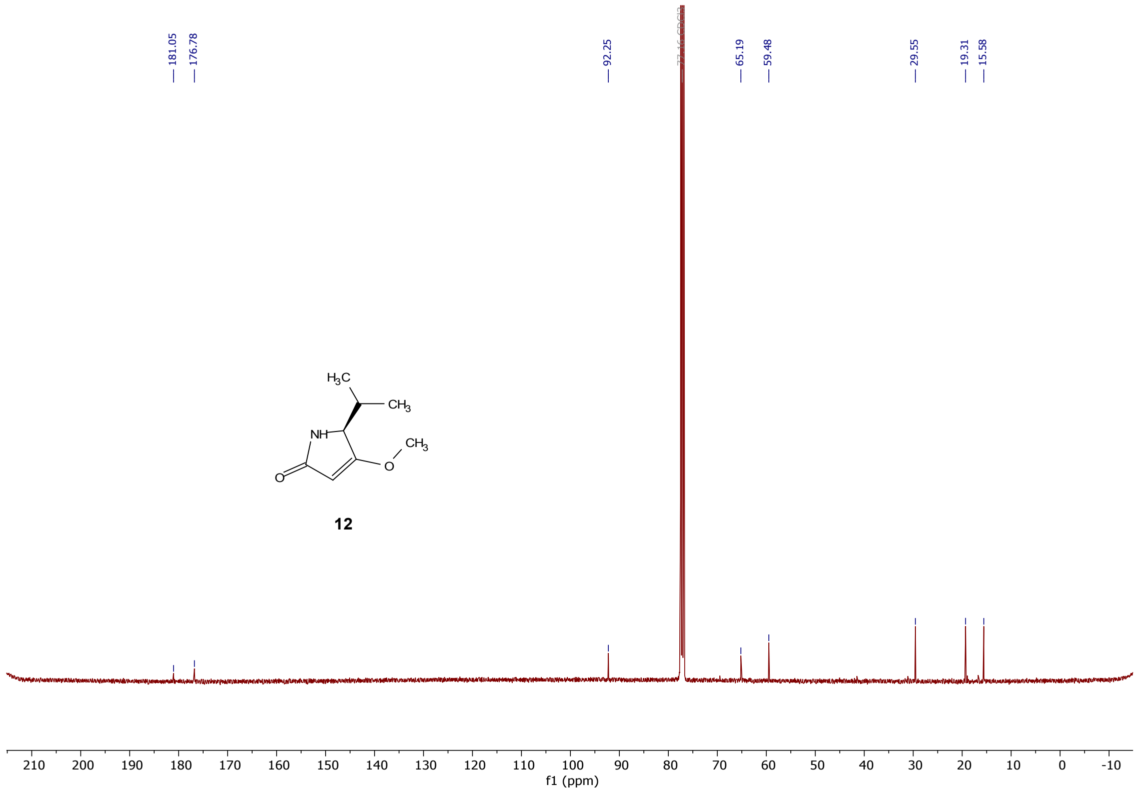
181.05
176.78
92.25
65.19
59.48
29.55
19.31
15.58
H3C
CH3
NH
CH3
O
O
12
f1 (ppm)

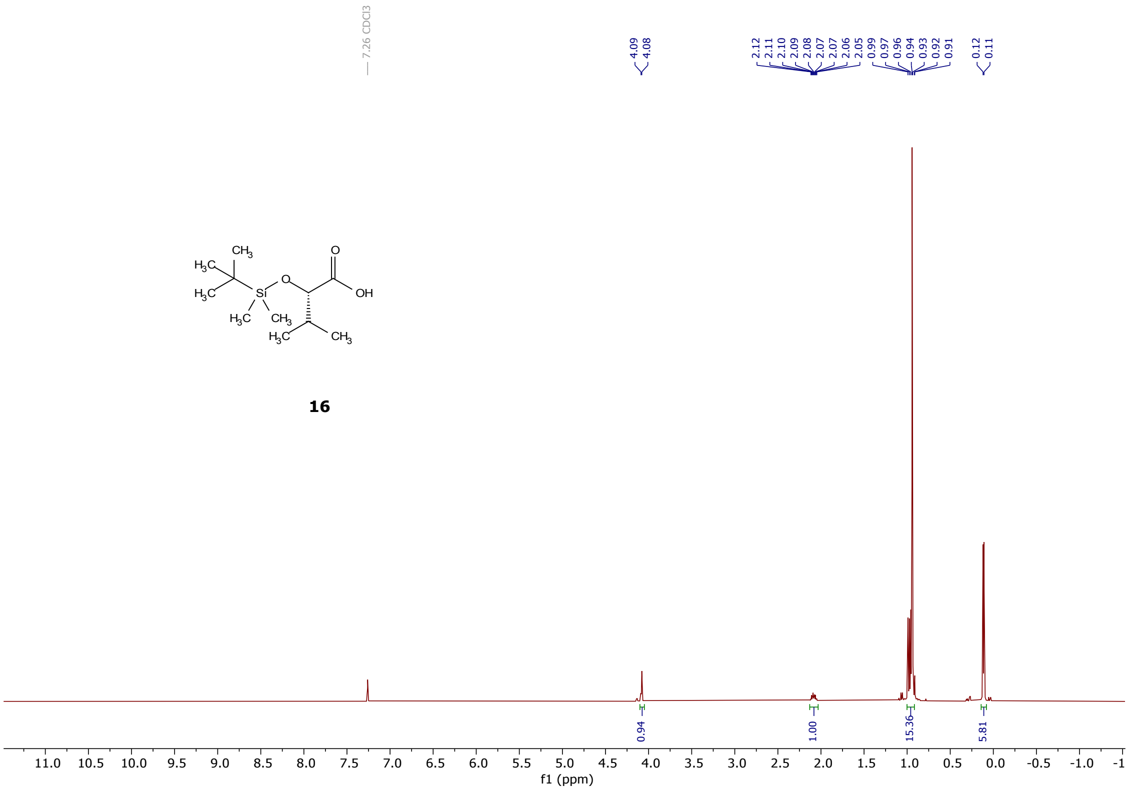
7.26 CDCl3
4.09
4.08
2.12
2.11
2.10
2.09
2.08
2.07
2.07
2.06
2.05
0.99
0.97
0.96
0.94
0.93
0.92
0.91
0.12
0.11
CH3
H3C
H3C
Si
O
O
OH
H3C
CH3
H3C
CH3
16
0.94
1.00
15.36
5.81
11.0 10.5 10.0 9.5 9.0 8.5 8.0 7.5 7.0 6.5 6.0 5.5 5.0 4.5 4.0 3.5 3.0 2.5 2.0 1.5 1.0 0.5 0.0 -0.5 -1.0 -1
f1 (ppm)

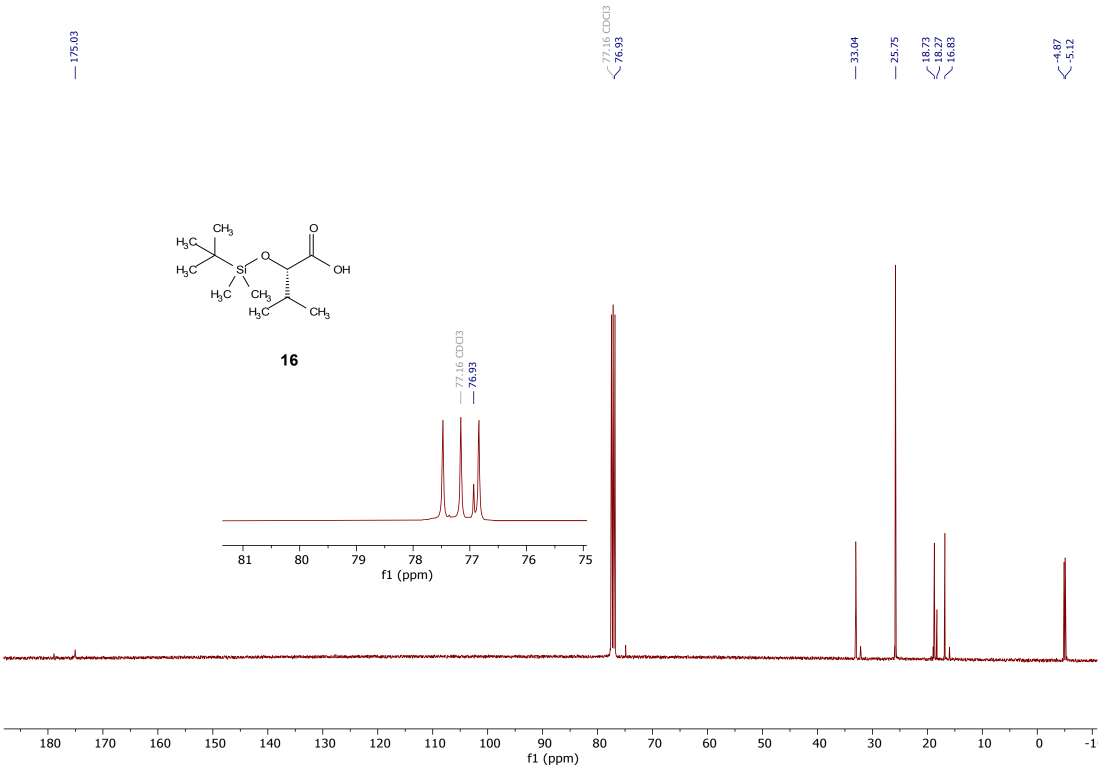
175.03
77.16 CDCl3
76.93
33.04
25.75
18.73
18.27
16.83
-4.87
-5.12
CH3
H3C
H3C
Si
O
O
OH
H3C
CH3
H3C
CH3
16
77.16 CDCl3
76.93
81 80 79 78 77 76 75
f1 (ppm)
180 170 160 150 140 130 120 110 100 90 80 70 60 50 40 30 20 10 0 -1
f1 (ppm)

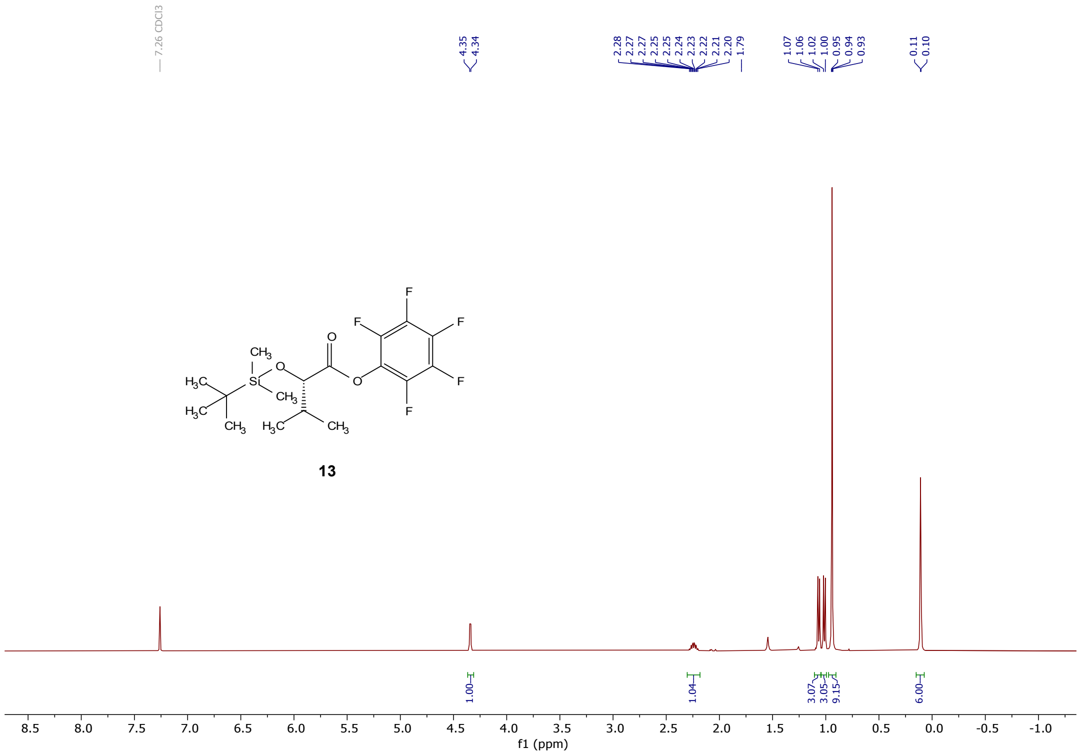

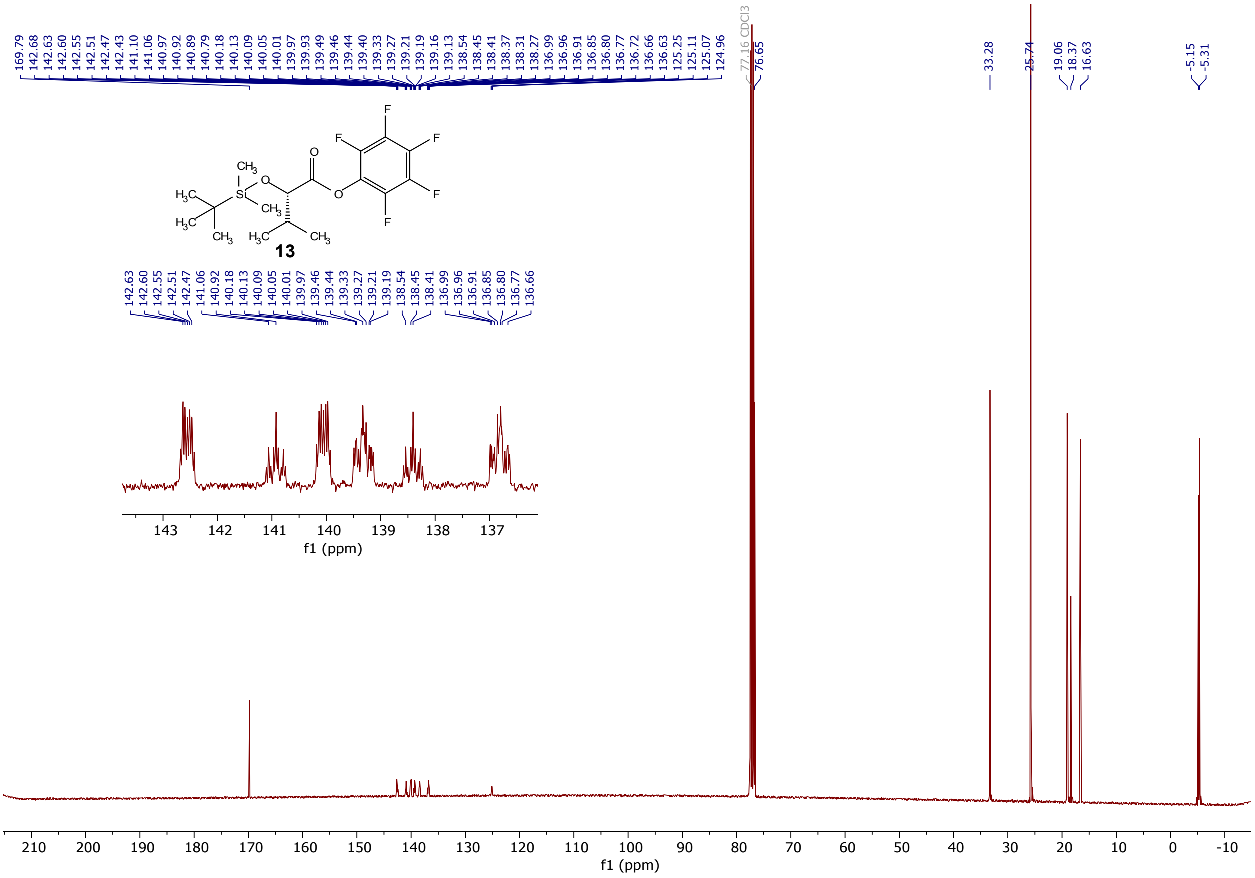

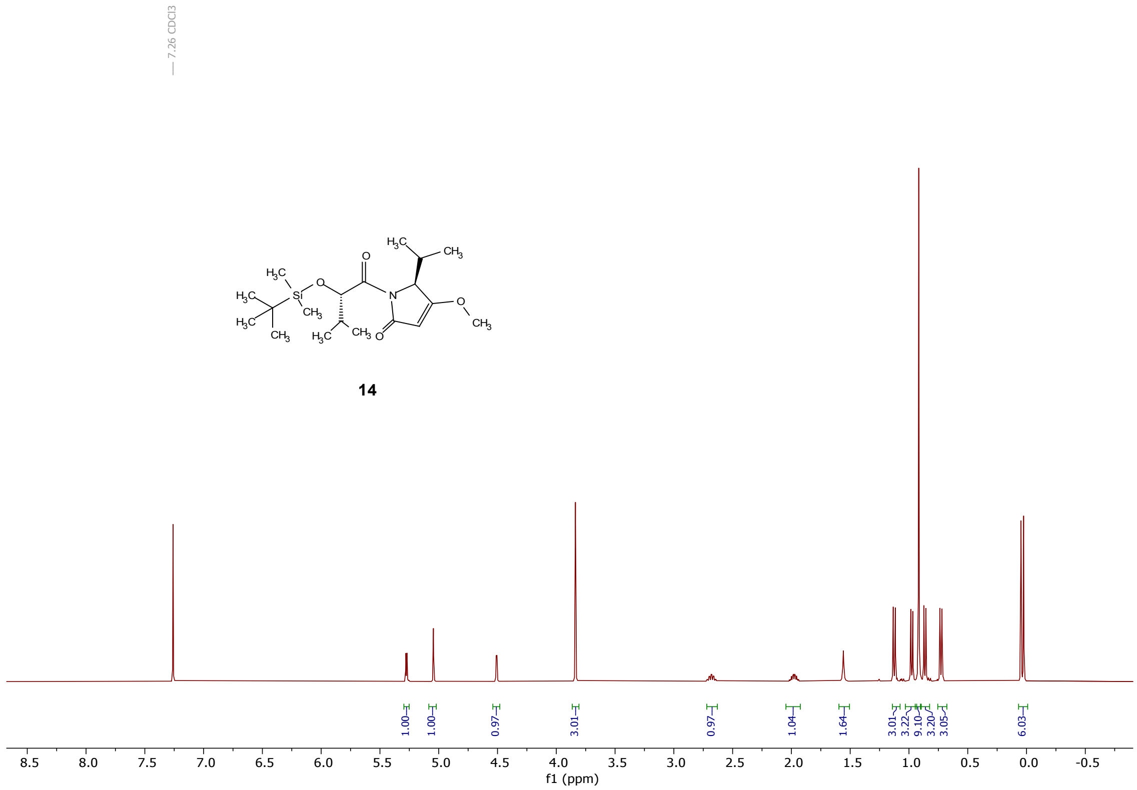
7.26 CDCl3
14
1.00
1.00
0.97
3.01
0.97
1.04
1.64
3.01
3.22
9.10
3.20
3.05
6.03
8.5 8.0 7.5 7.0 6.5 6.0 5.5 5.0 4.5 4.0 3.5 3.0 2.5 2.0 1.5 1.0 0.5 0.0 -0.5
f1 (ppm)

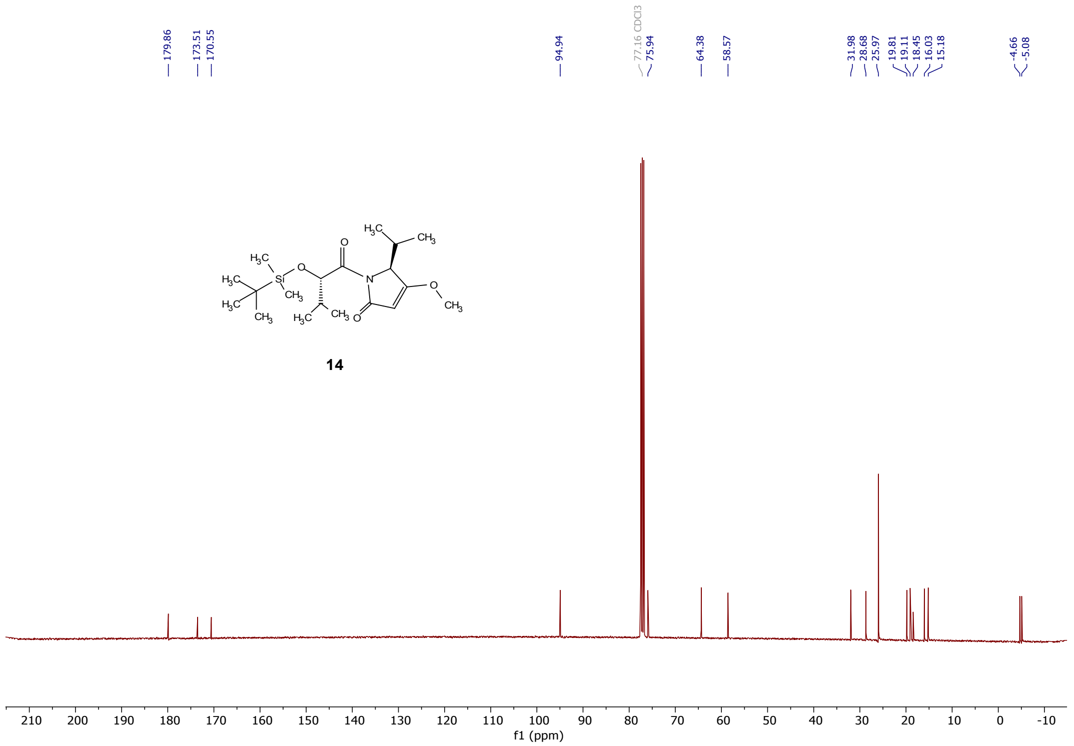
179.86
173.51
170.55
94.94
77.16 CDCl3
75.94
64.38
58.57
31.98
28.68
25.97
19.81
19.11
18.45
16.03
15.18
-4.66
-5.08
14
210 200 190 180 170 160 150 140 130 120 110 100 90 80 70 60 50 40 30 20 10 0 -10
f1 (ppm)

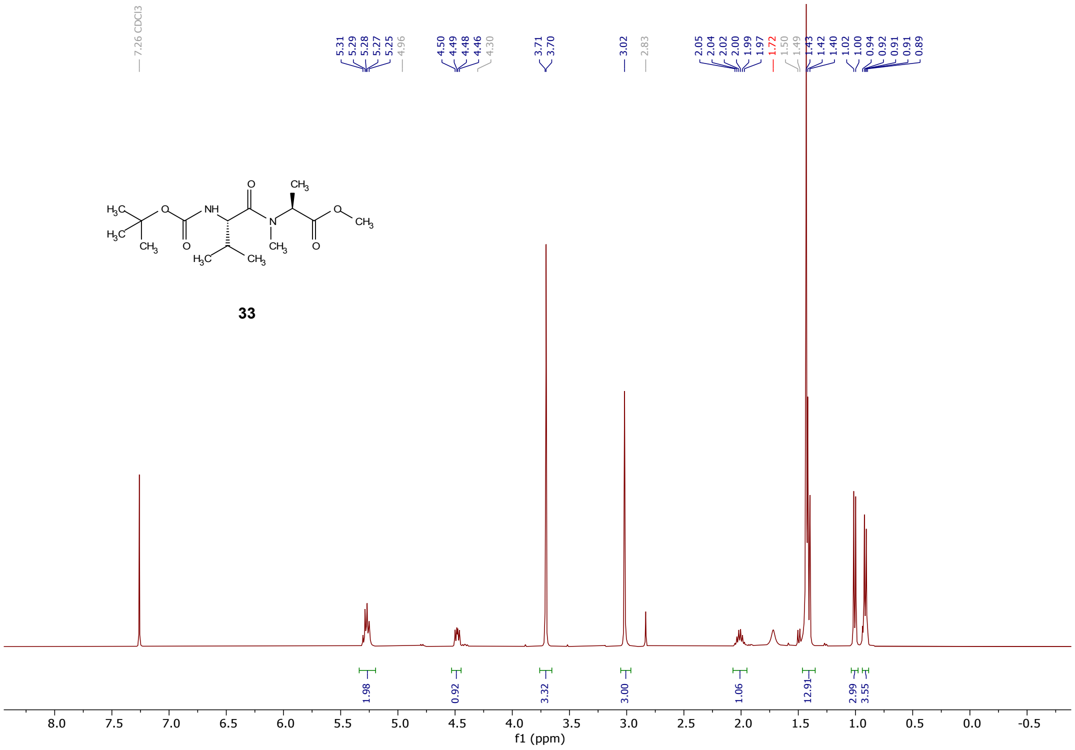
7.26 CDCl3
5.31
5.29
5.28
5.27
5.25
4.96
4.50
4.49
4.48
4.46
4.30
3.71
3.70
3.02
2.83
2.05
2.04
2.02
2.00
1.99
1.97
1.72
1.50
1.49
1.43
1.42
1.40
1.02
1.00
0.94
0.92
0.91
0.89
33
1.98
0.92
3.32
3.00
1.06
12.91
2.99
3.55
8.0 7.5 7.0 6.5 6.0 5.5 5.0 4.5 4.0 3.5 3.0 2.5 2.0 1.5 1.0 0.5 0.0 -0.5
f1 (ppm)

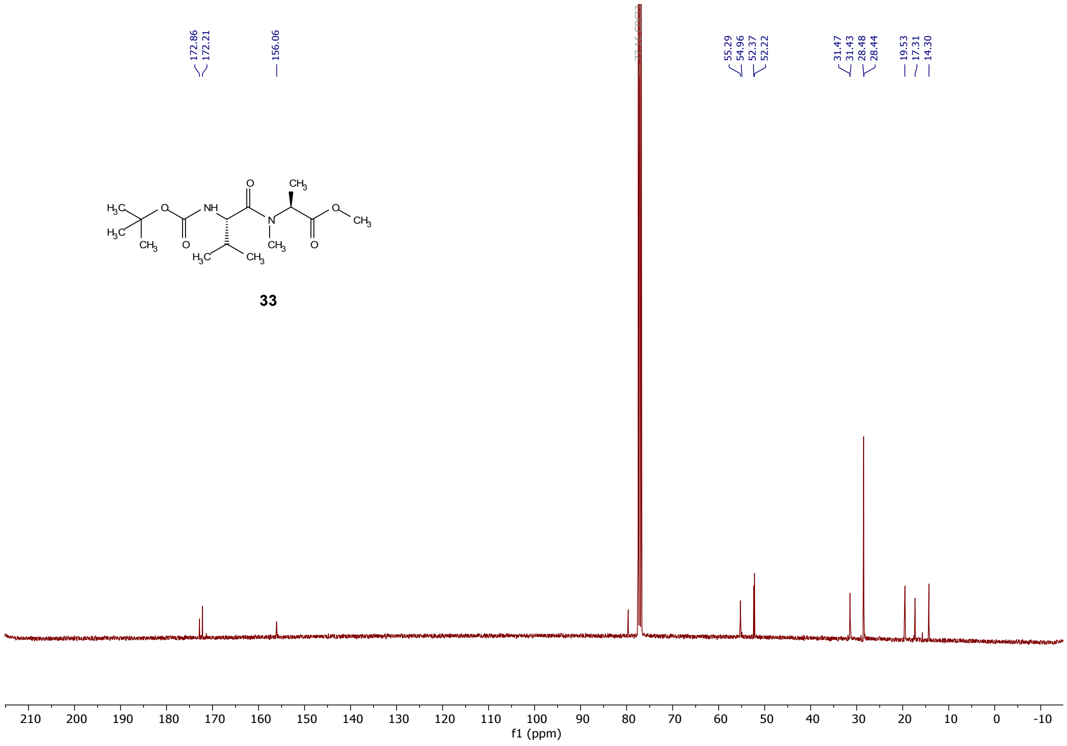
172.86
172.21
156.06
55.29
54.96
52.37
52.22
31.47
31.43
28.48
28.44
19.53
17.31
14.30
33
210 200 190 180 170 160 150 140 130 120 110 100 90 80 70 60 50 40 30 20 10 0 -10
f1 (ppm)

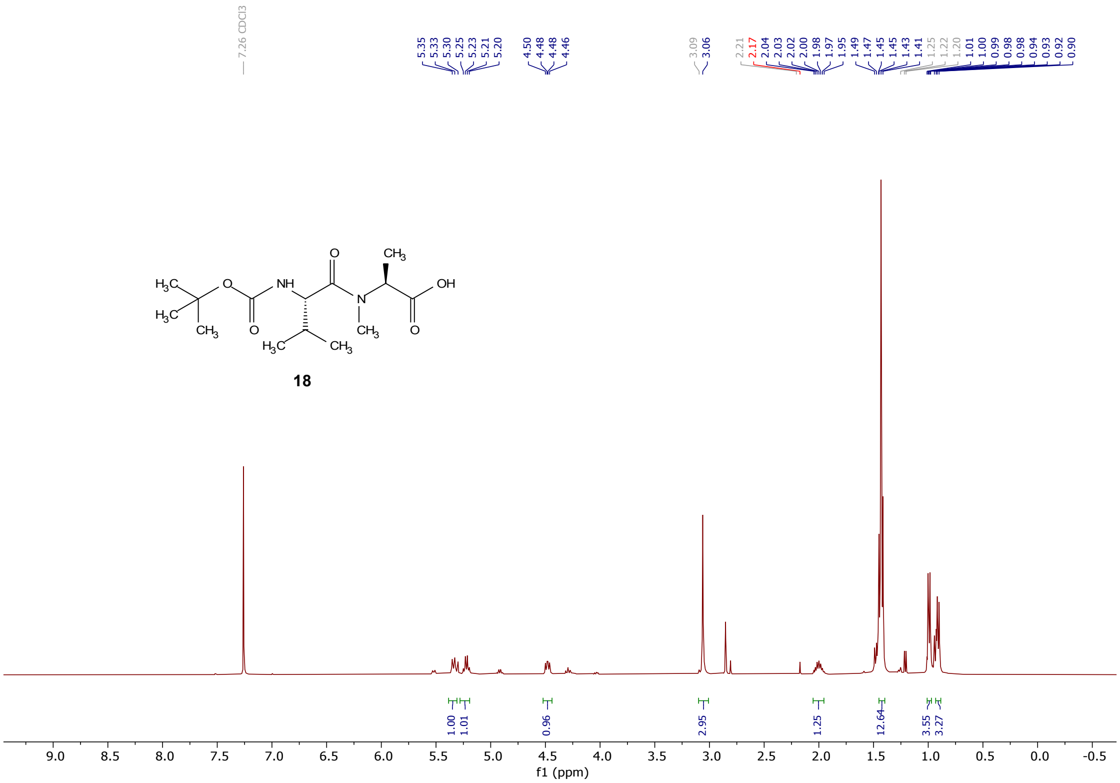
7.26 CDCl3
5.35 5.33 5.30 5.25 5.23 5.21 5.20
4.50 4.48 4.48 4.46
3.09 3.06
2.21 2.17 2.04 2.03 2.02 2.00 1.98 1.97 1.95 1.49 1.47 1.45 1.45 1.43 1.41 1.25 1.22 1.20 1.01 1.00 0.99 0.98 0.98 0.94 0.93 0.92 0.90
18
1.00 1.01 0.96 2.95 1.25 12.64 3.55 3.27
f1 (ppm)

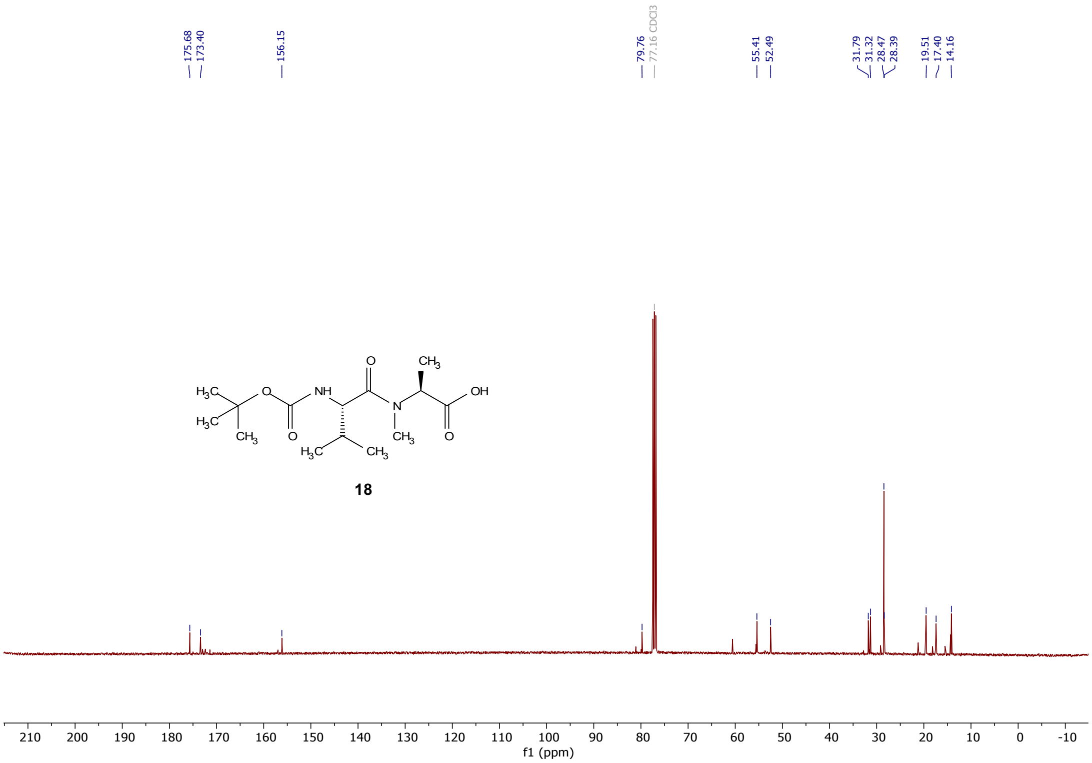
175.68 173.40 156.15 79.76 77.16 CDCl3 55.41 52.49 31.79 31.32 28.47 28.39 19.51 17.40 14.16
18
f1 (ppm)

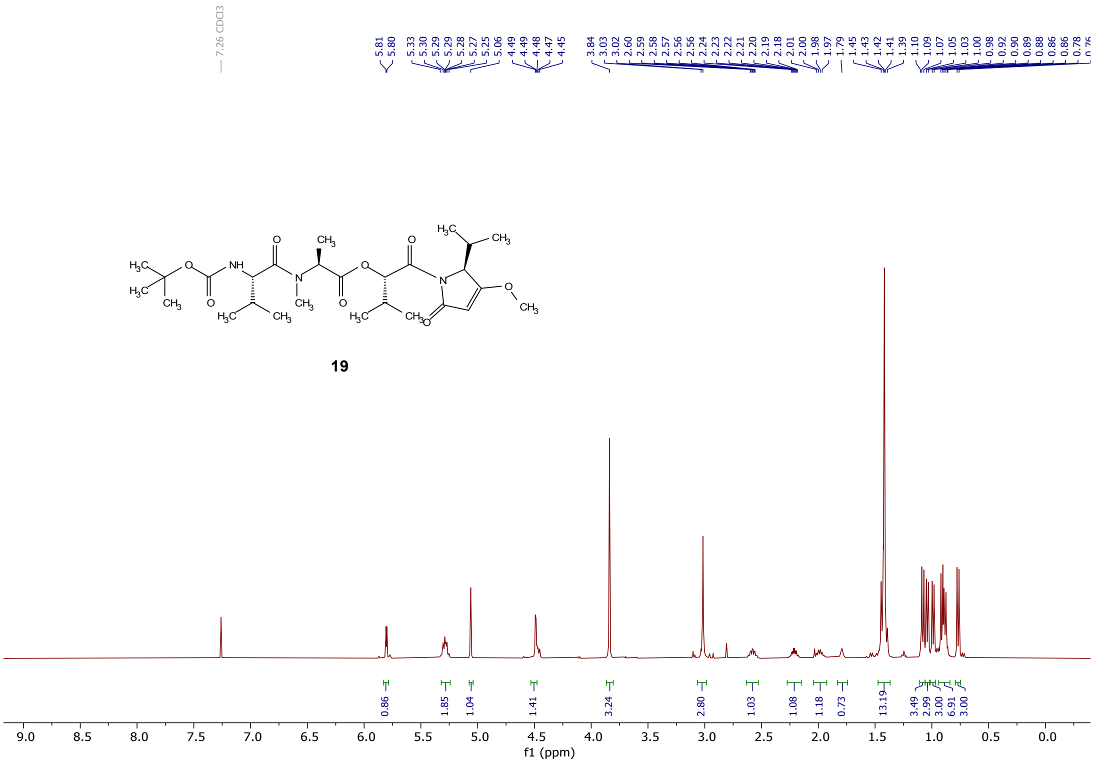
7.26 CDCl3
5.81
5.80
5.33
5.30
5.29
5.29
5.28
5.27
5.25
5.06
4.49
4.48
4.47
4.45
3.84
3.03
3.02
2.60
2.59
2.58
2.57
2.56
2.56
2.24
2.23
2.22
2.21
2.20
2.19
2.18
2.01
2.00
1.98
1.97
1.79
1.45
1.43
1.42
1.41
1.39
1.10
1.09
1.07
1.05
1.03
1.00
0.98
0.92
0.90
0.89
0.88
0.86
0.86
0.78
19
0.86
1.85
1.04
1.41
3.24
2.80
1.03
1.08
1.18
0.73
13.19
3.49
2.99
3.00
6.91
3.00
9.0 8.5 8.0 7.5 7.0 6.5 6.0 5.5 5.0 4.5 4.0 3.5 3.0 2.5 2.0 1.5 1.0 0.5 0.0
f1 (ppm)

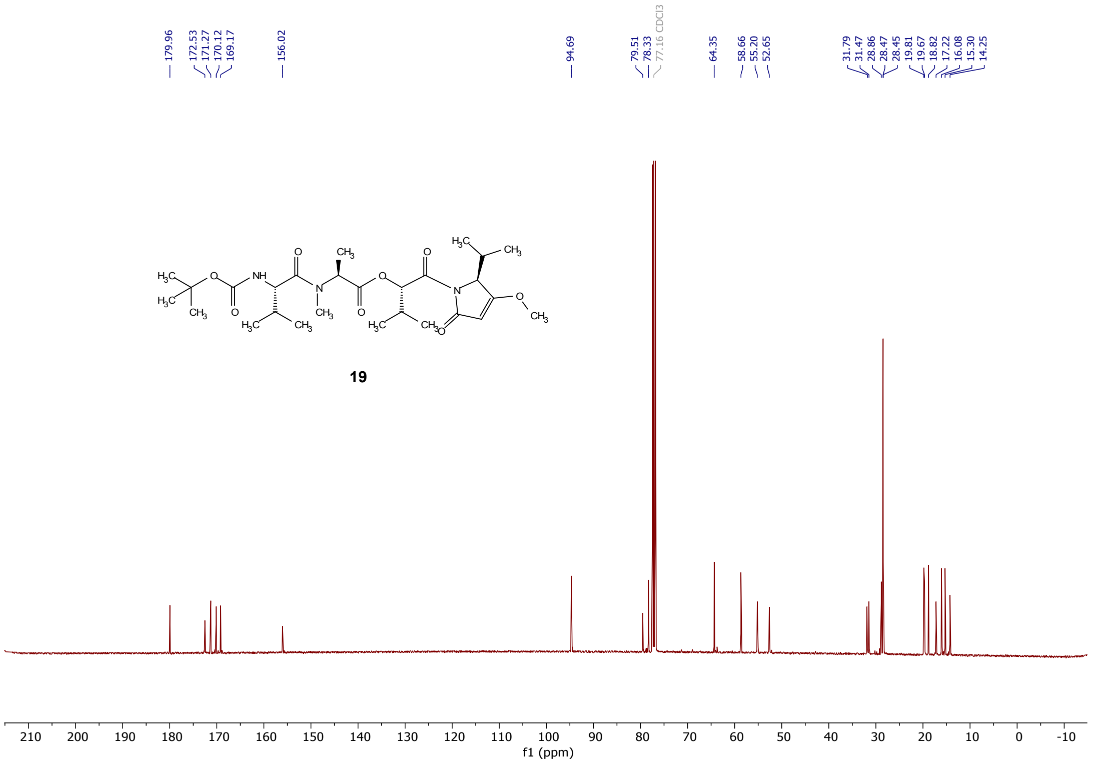
179.96
172.53
171.27
170.12
169.17
156.02
94.69
79.51
78.33
77.16 CDCl3
64.35
58.66
55.20
52.65
31.79
31.47
28.86
28.47
28.45
19.81
19.67
18.82
17.22
16.08
15.30
14.25
19
210 200 190 180 170 160 150 140 130 120 110 100 90 80 70 60 50 40 30 20 10 0 -10
f1 (ppm)

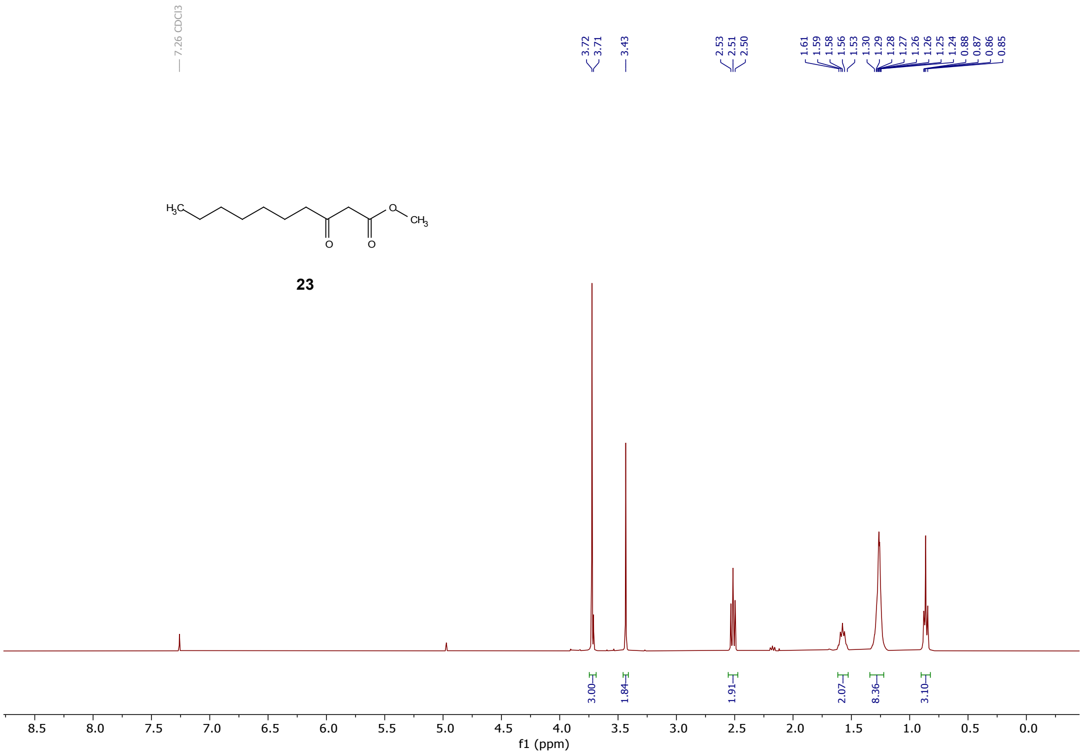
7.26 CDCl3
3.72
3.71
3.43
2.53
2.51
2.50
1.61
1.59
1.58
1.56
1.53
1.30
1.29
1.28
1.27
1.26
1.26
1.25
1.24
0.88
0.87
0.86
0.85
H3C
O
O
O
CH3
23
3.00
1.84
1.91
2.07
8.36
3.10
8.5 8.0 7.5 7.0 6.5 6.0 5.5 5.0 4.5 4.0 3.5 3.0 2.5 2.0 1.5 1.0 0.5 0.0
f1 (ppm)

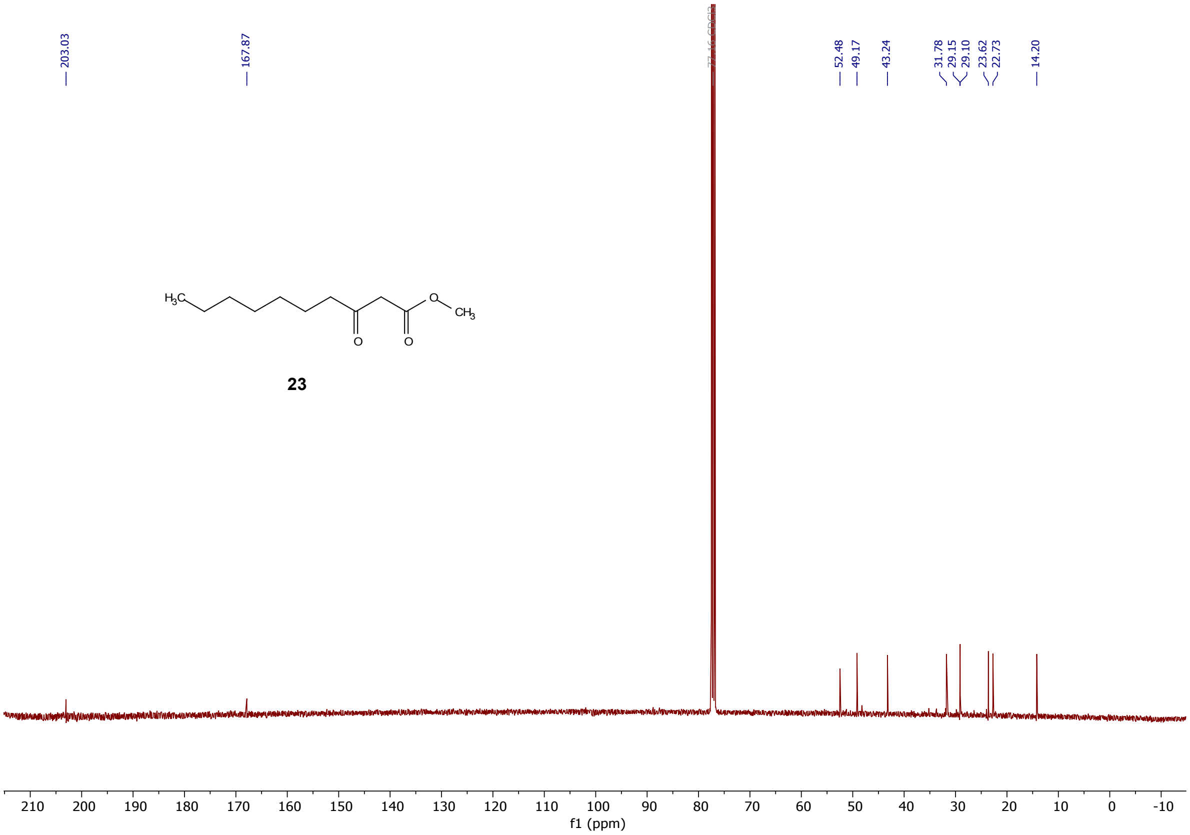
203.03
167.87
52.48
49.17
43.24
31.78
29.15
29.10
23.62
22.73
14.20
H3C
O
O
O
CH3
23
210 200 190 180 170 160 150 140 130 120 110 100 90 80 70 60 50 40 30 20 10 0 -10
f1 (ppm)

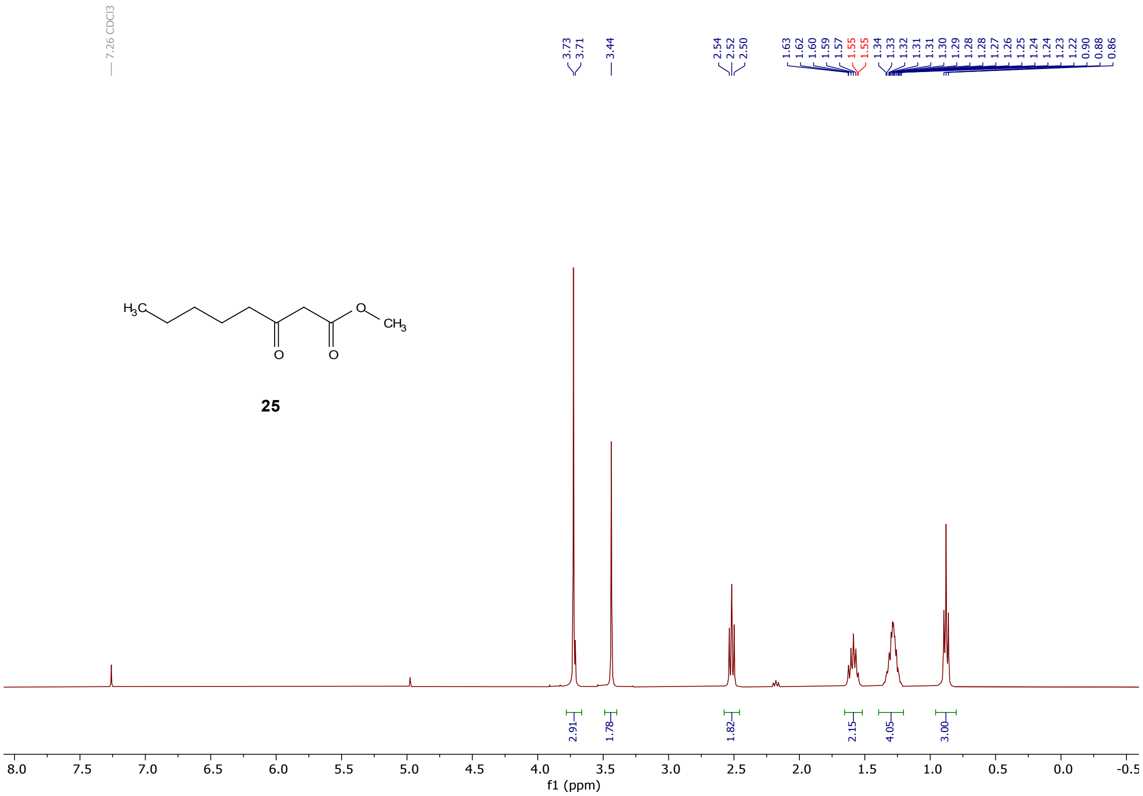
7.26 CDCl3
3.73
3.71
3.44
2.54
2.52
2.50
1.63
1.62
1.60
1.59
1.57
1.55
1.55
1.34
1.33
1.32
1.31
1.31
1.30
1.29
1.28
1.28
1.27
1.26
1.25
1.24
1.24
1.23
1.22
0.90
0.88
0.86
H_3C
O
CH_3
O
O
25
2.91
1.78
1.82
2.15
4.05
3.00
8.0 7.5 7.0 6.5 6.0 5.5 5.0 4.5 4.0 3.5 3.0 2.5 2.0 1.5 1.0 0.5 0.0 -0.5
f1 (ppm)

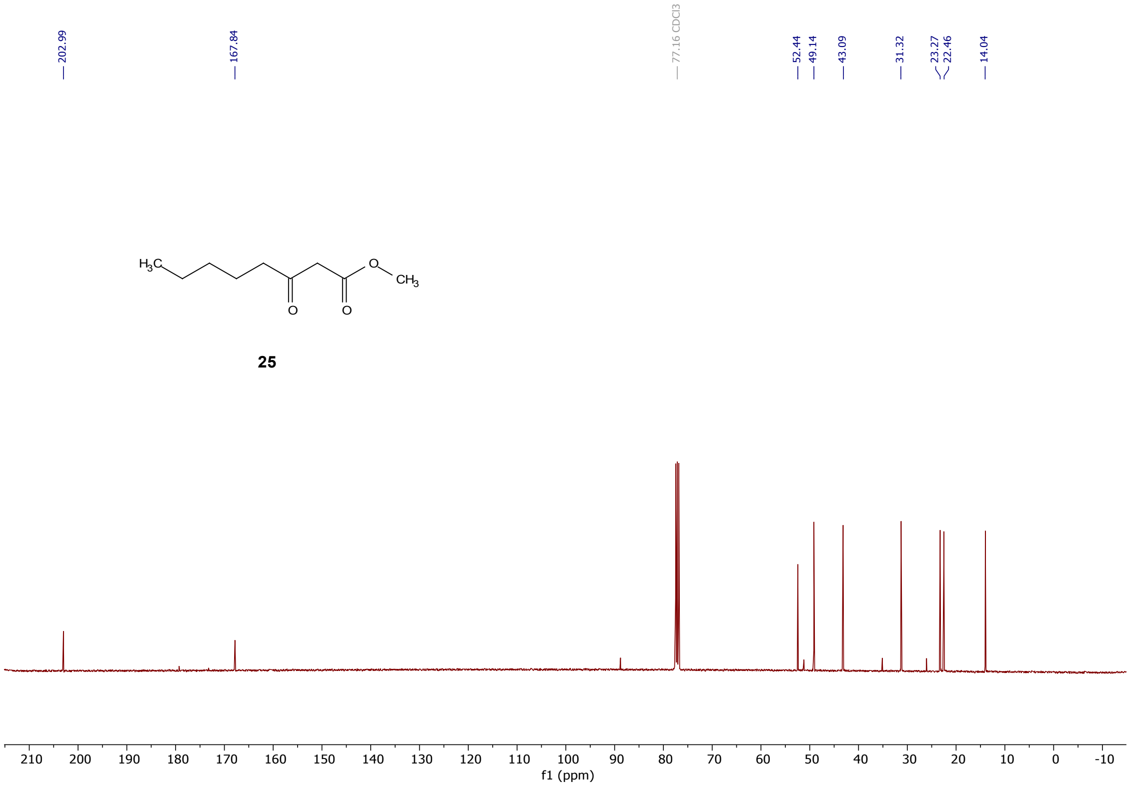
202.99
167.84
77.16 CDCl3
52.44
49.14
43.09
31.32
23.27
22.46
14.04
H_3C
O
CH_3
O
O
25
210 200 190 180 170 160 150 140 130 120 110 100 90 80 70 60 50 40 30 20 10 0 -10
f1 (ppm)

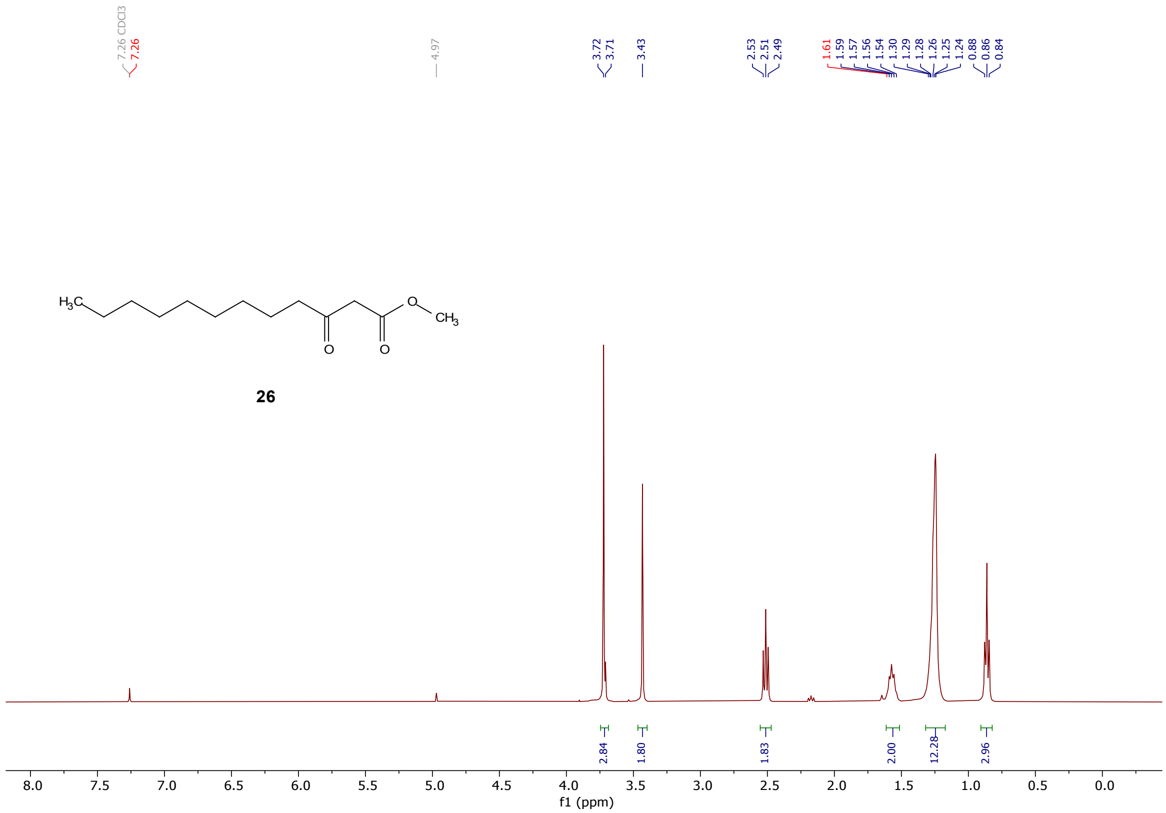
7.26 CDCl3
7.26
4.97
3.72
3.71
3.43
2.53
2.51
2.49
1.61
1.59
1.57
1.56
1.54
1.30
1.29
1.28
1.26
1.25
1.24
0.88
0.86
0.84
2.84
1.80
1.83
2.00
12.28
2.96
8.0 7.5 7.0 6.5 6.0 5.5 5.0 4.5 4.0 3.5 3.0 2.5 2.0 1.5 1.0 0.5 0.0
f1 (ppm)

26

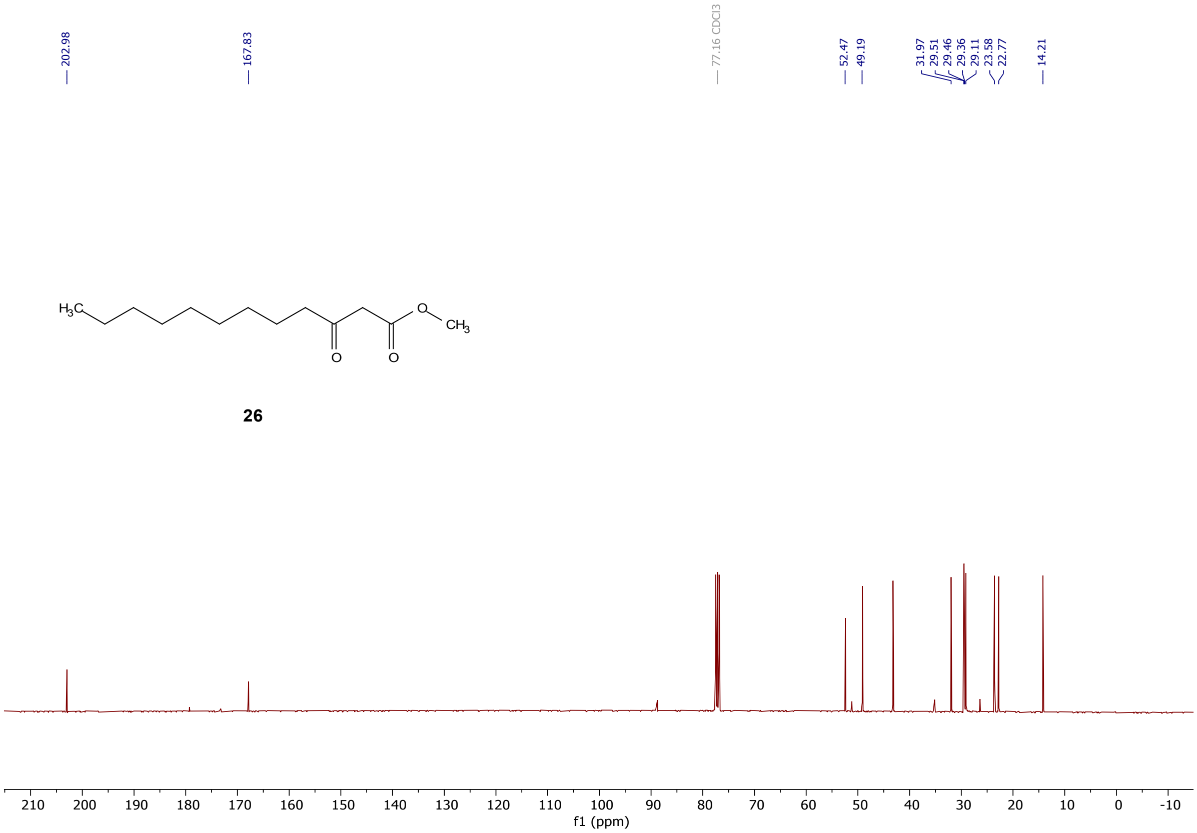
202.98
167.83
77.16 CDCl3
52.47
49.19
31.97
29.51
29.46
29.36
29.11
23.58
22.77
14.21
210 200 190 180 170 160 150 140 130 120 110 100 90 80 70 60 50 40 30 20 10 0 -10
f1 (ppm)

26

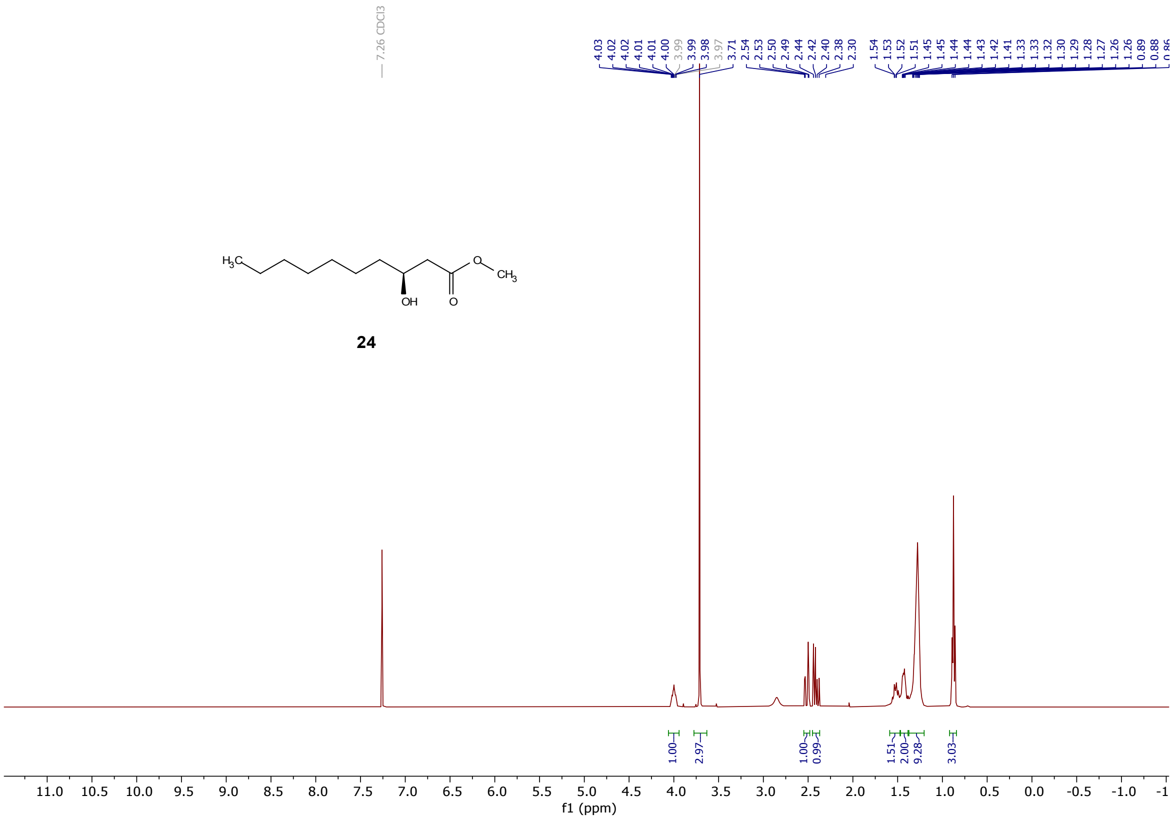
7.26 CDCl3
4.03
4.02
4.02
4.01
4.01
4.00
3.99
3.99
3.98
3.97
3.71
2.54
2.53
2.50
2.49
2.44
2.42
2.40
2.38
2.30
1.54
1.53
1.52
1.51
1.45
1.45
1.44
1.44
1.43
1.42
1.41
1.33
1.33
1.32
1.30
1.29
1.28
1.27
1.26
1.26
0.89
0.88
H_3C
OH
O
O
CH_3
24
1.00
2.97
1.00
0.99
1.51
2.00
9.28
3.03
11.0 10.5 10.0 9.5 9.0 8.5 8.0 7.5 7.0 6.5 6.0 5.5 5.0 4.5 4.0 3.5 3.0 2.5 2.0 1.5 1.0 0.5 0.0 -0.5 -1.0
f1 (ppm)

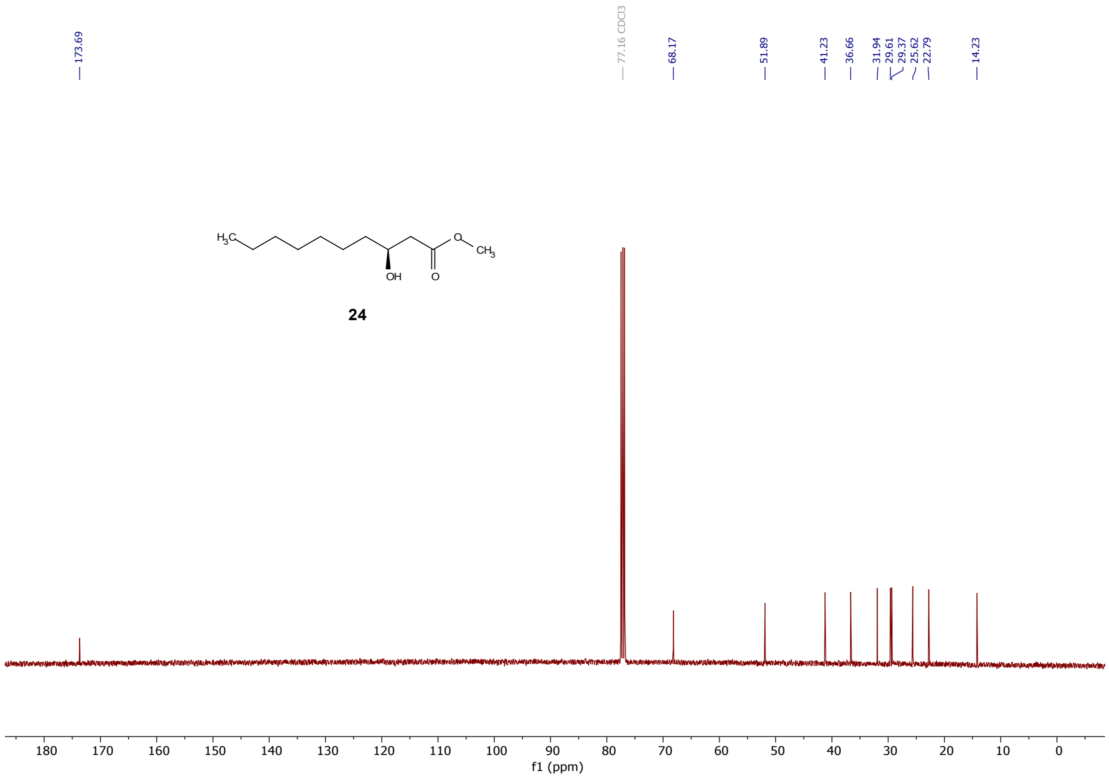
173.69
77.16 CDCl3
68.17
51.89
41.23
36.66
31.94
29.61
29.37
25.62
22.79
14.23
H_3C
OH
O
O
CH_3
24
180 170 160 150 140 130 120 110 100 90 80 70 60 50 40 30 20 10 0
f1 (ppm)

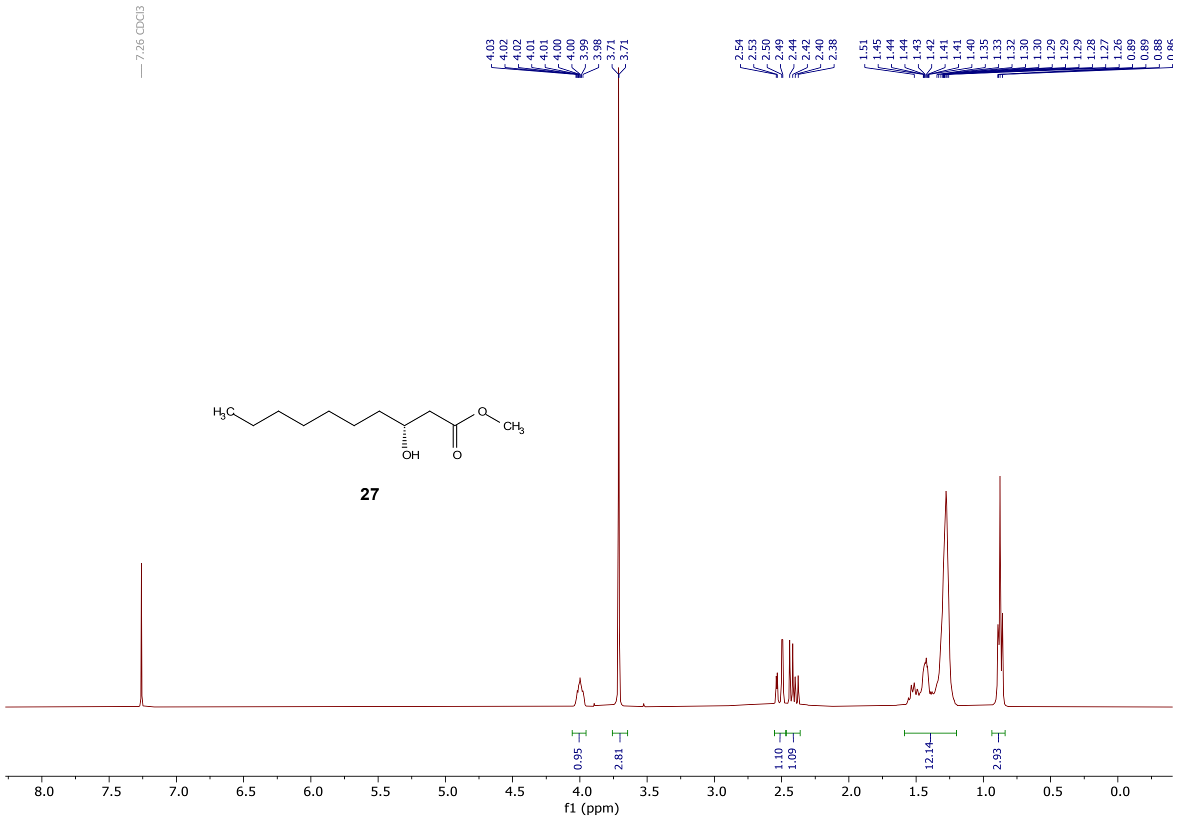
7.26 CDCl3
4.03
4.02
4.02
4.01
4.01
4.00
4.00
3.99
3.98
3.71
3.71
2.54
2.53
2.50
2.49
2.44
2.42
2.40
2.38
1.51
1.45
1.44
1.44
1.43
1.42
1.41
1.41
1.40
1.35
1.33
1.32
1.30
1.30
1.29
1.29
1.29
1.28
1.27
1.26
0.89
0.89
0.88
H_3C
OH
O
O
CH_3
27
0.95
2.81
1.10
1.09
12.14
2.93
f1 (ppm)

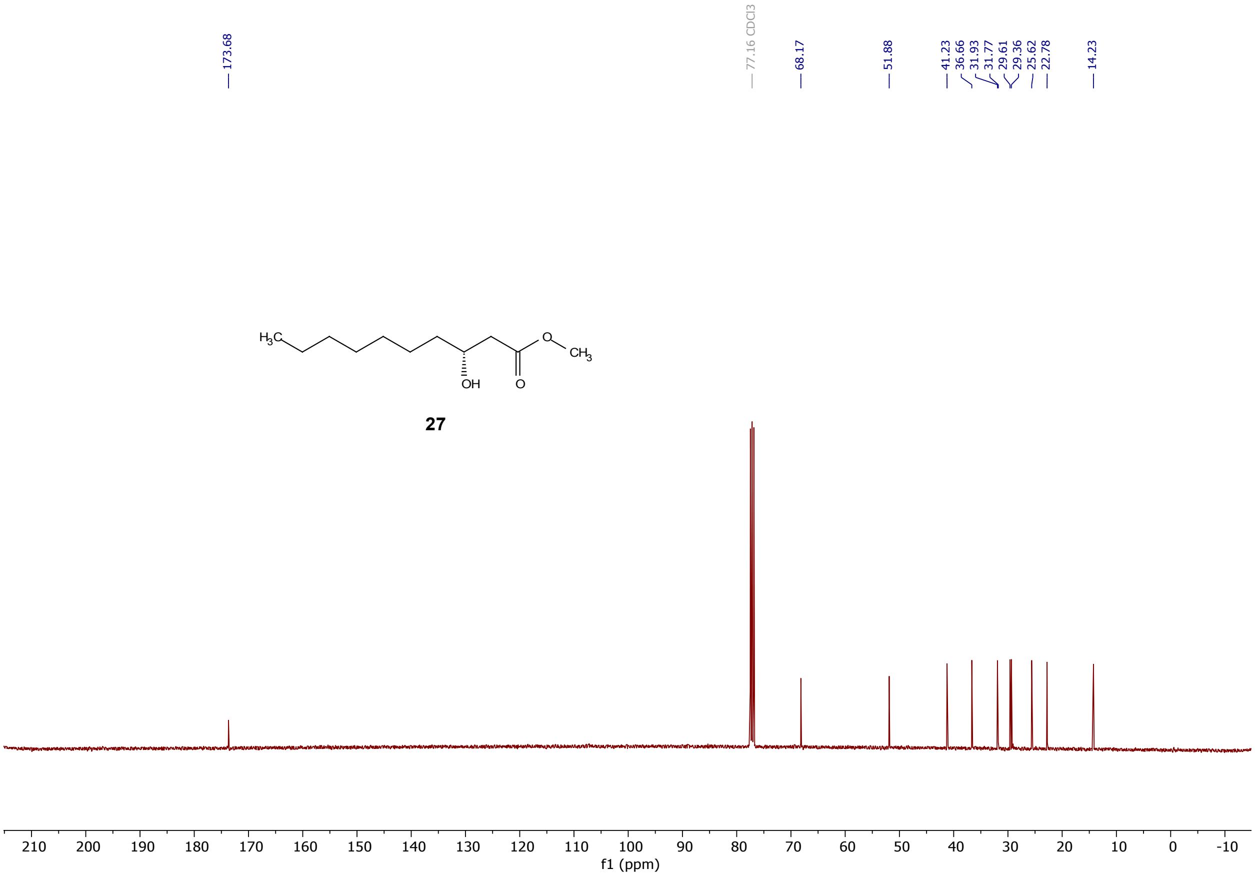
173.68
77.16 CDCl3
68.17
51.88
41.23
36.66
31.93
31.77
29.61
29.36
25.62
22.78
14.23
H_3C
OH
O
O
CH_3
27
f1 (ppm)

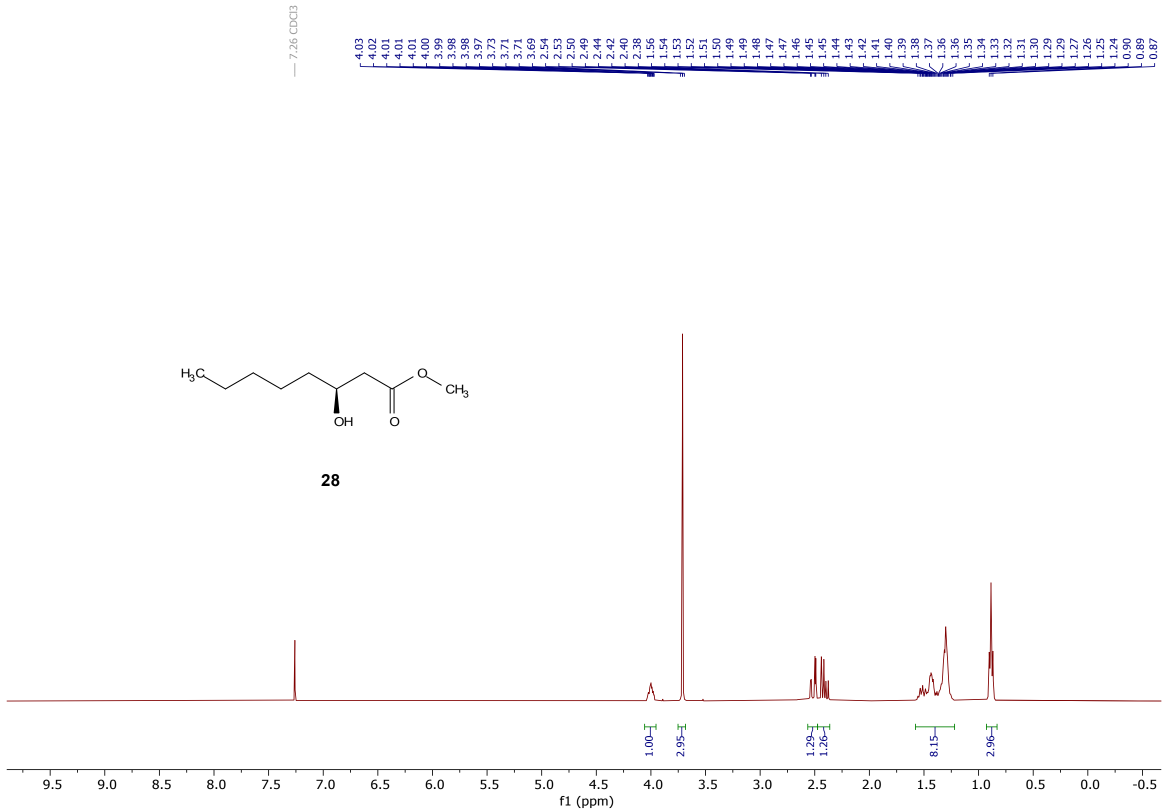
7.26 CDCl3
4.03 4.02 4.01 4.01 4.01 4.00 3.99 3.98 3.98 3.97 3.73 3.71 3.71 3.69 2.54 2.53 2.50 2.49 2.44 2.42 2.40 2.38 1.56 1.54 1.53 1.52 1.51 1.50 1.49 1.49 1.48 1.47 1.47 1.46 1.45 1.45 1.44 1.43 1.42 1.41 1.40 1.39 1.38 1.37 1.36 1.36 1.35 1.34 1.33 1.32 1.31 1.30 1.29 1.29 1.27 1.26 1.25 1.24 0.90 0.89 0.87
H3C
OH
O
O
CH3
28
1.00
2.95
1.29
1.26
8.15
2.96
9.5 9.0 8.5 8.0 7.5 7.0 6.5 6.0 5.5 5.0 4.5 4.0 3.5 3.0 2.5 2.0 1.5 1.0 0.5 0.0 -0.5
f1 (ppm)

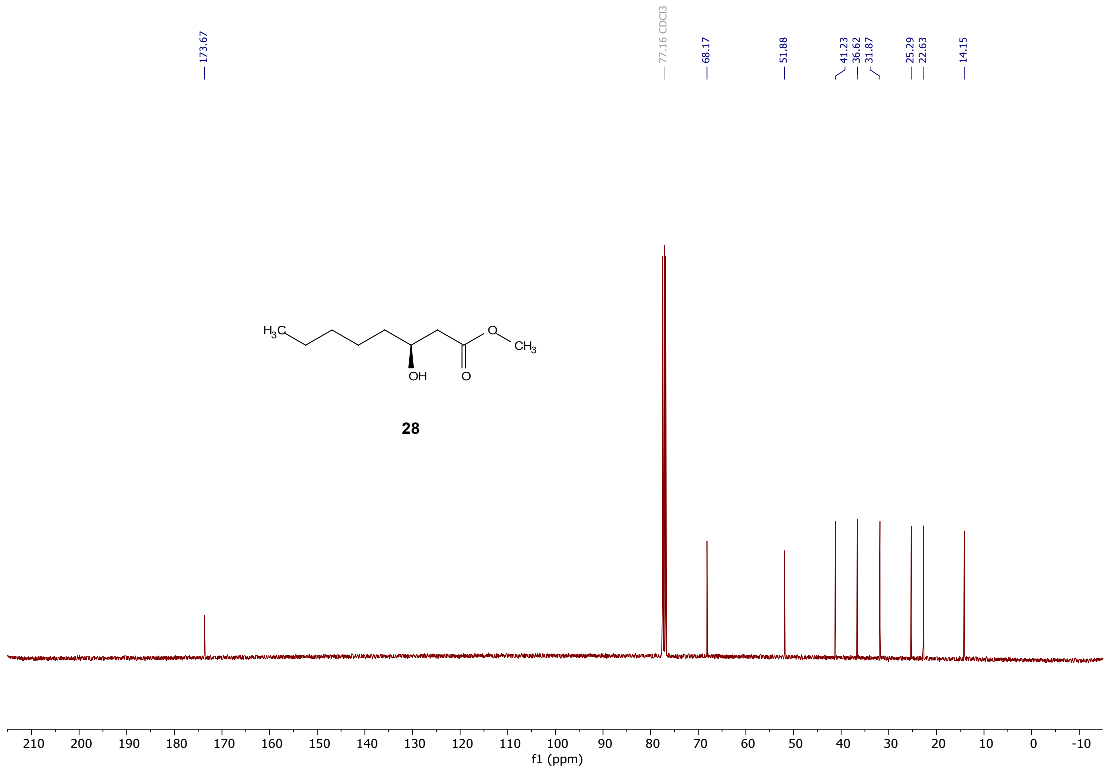
173.67
77.16 CDCl3
68.17
51.88
41.23
36.62
31.87
25.29
22.63
14.15
H3C
OH
O
O
CH3
28
210 200 190 180 170 160 150 140 130 120 110 100 90 80 70 60 50 40 30 20 10 0 -10
f1 (ppm)

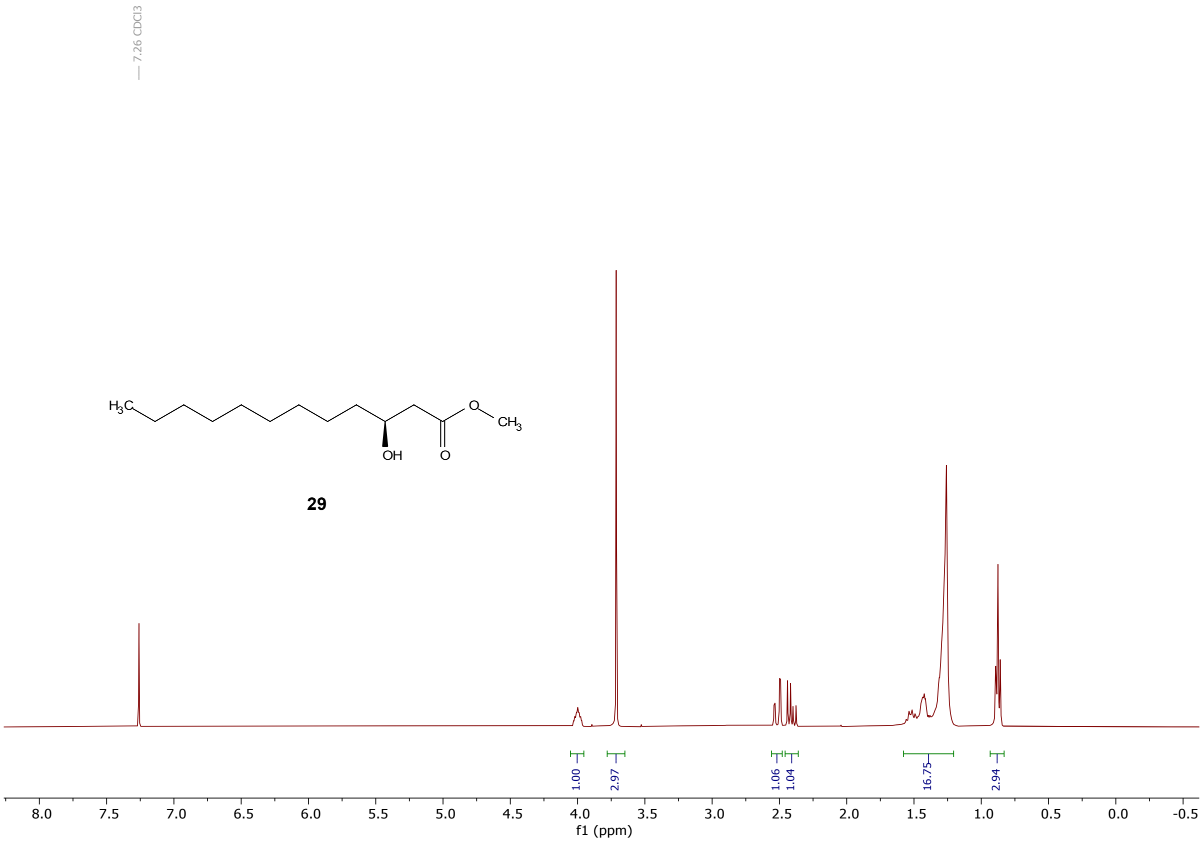
7.26 CDCl3
H_3C
OH
O
O
CH_3
29
1.00
2.97
1.06
1.04
16.75
2.94
8.0 7.5 7.0 6.5 6.0 5.5 5.0 4.5 4.0 3.5 3.0 2.5 2.0 1.5 1.0 0.5 0.0 -0.5
f1 (ppm)

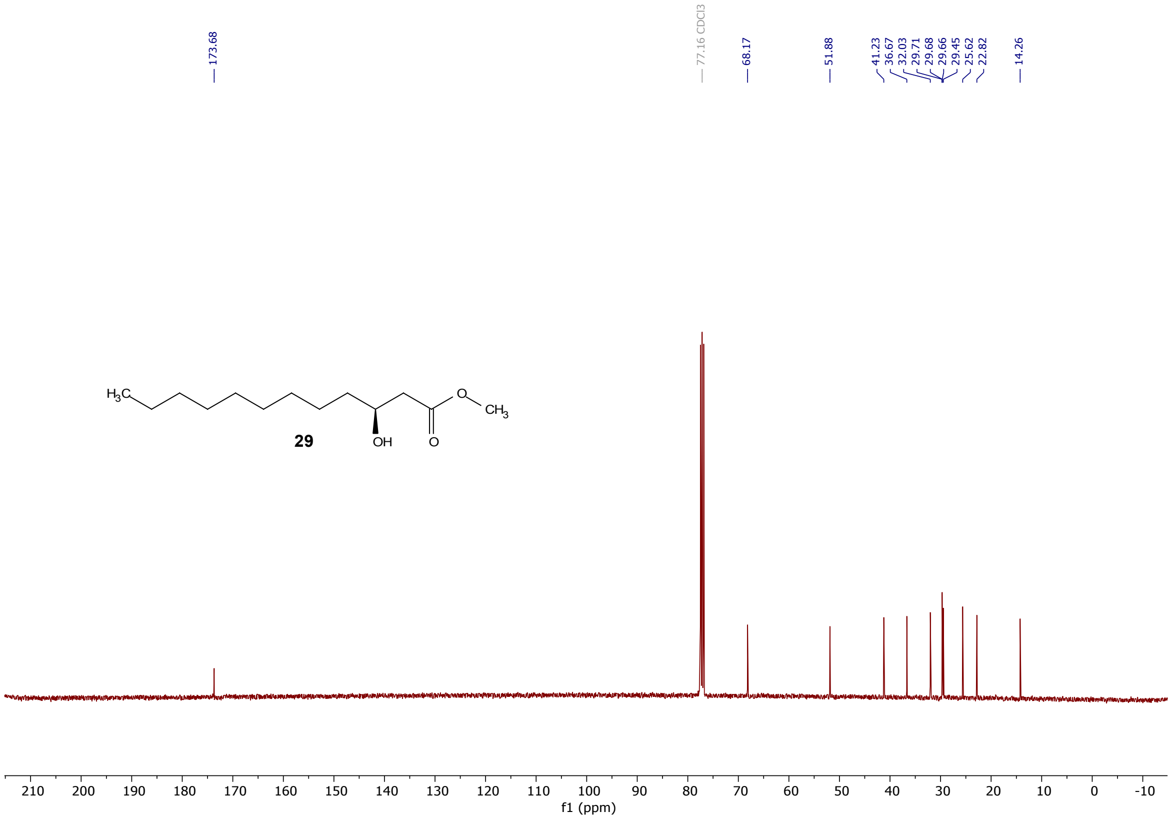
173.68
77.16 CDCl3
68.17
51.88
41.23
36.67
32.03
29.71
29.68
29.66
29.45
25.62
22.82
14.26
H_3C
29
OH
O
O
CH_3
210 200 190 180 170 160 150 140 130 120 110 100 90 80 70 60 50 40 30 20 10 0 -10
f1 (ppm)

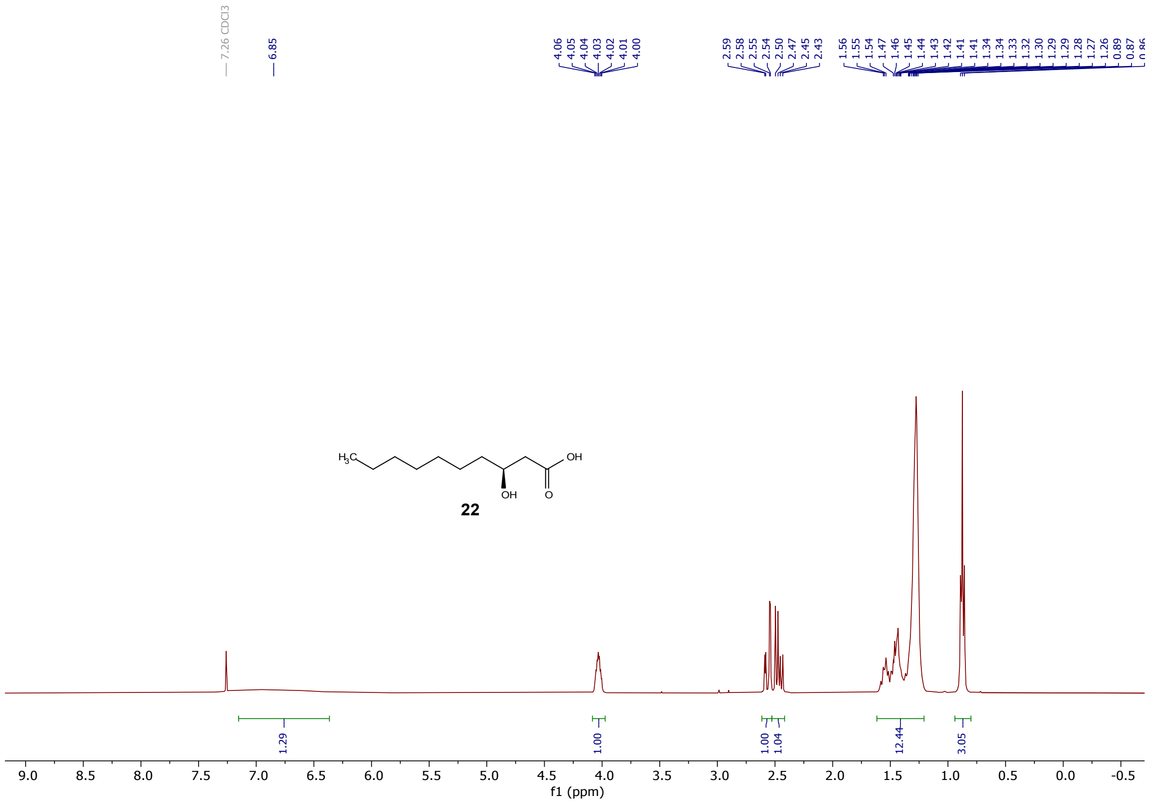
7.26 CDCl3
6.85
4.06
4.05
4.04
4.03
4.02
4.01
4.00
2.59
2.58
2.55
2.54
2.50
2.47
2.45
2.43
1.56
1.55
1.54
1.47
1.46
1.45
1.44
1.43
1.42
1.41
1.41
1.34
1.33
1.32
1.30
1.29
1.29
1.28
1.27
1.26
0.89
0.87
H3C
OH
OH
O
22
1.29
1.00
1.00
1.04
12.44
3.05
9.0 8.5 8.0 7.5 7.0 6.5 6.0 5.5 5.0 4.5 4.0 3.5 3.0 2.5 2.0 1.5 1.0 0.5 0.0 -0.5
f1 (ppm)

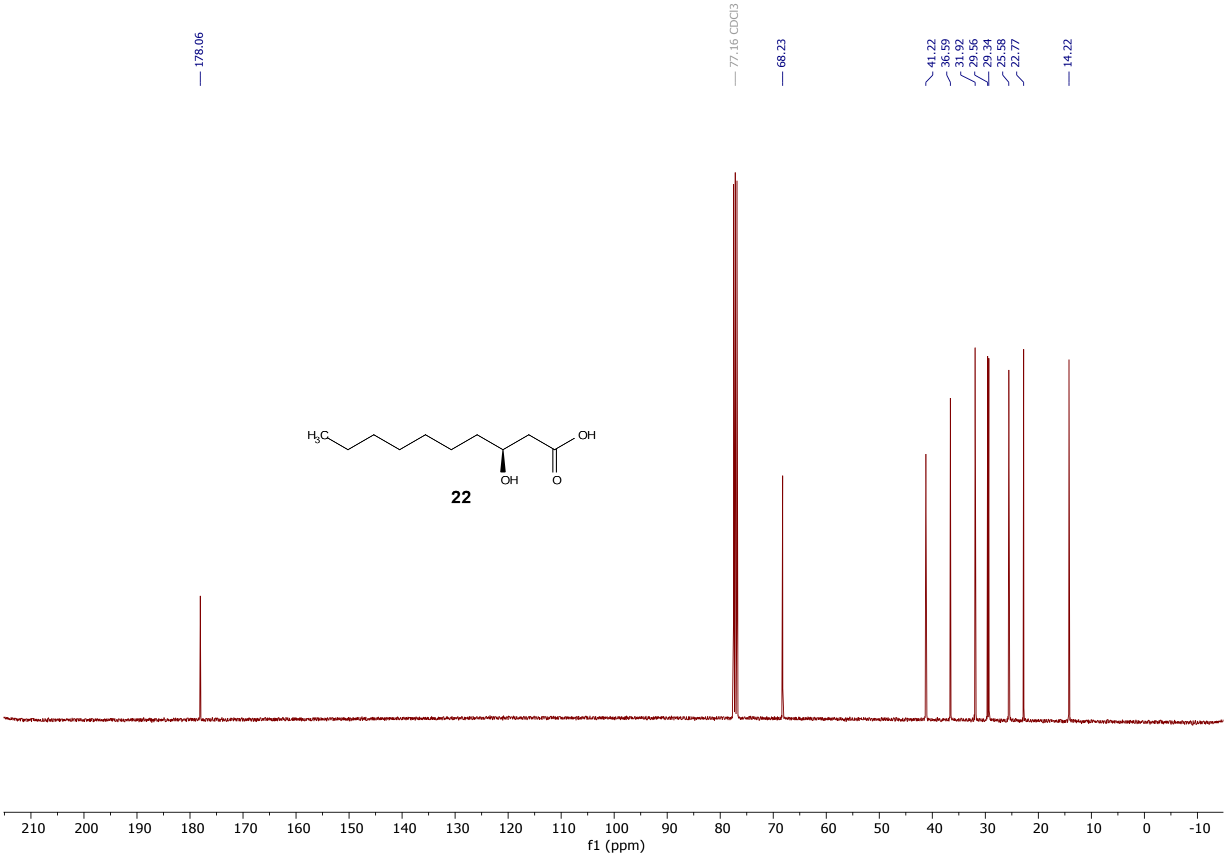
178.06
77.16 CDCl3
68.23
41.22
36.59
31.92
29.56
29.34
25.58
22.77
14.22
H3C
OH
OH
O
22
210 200 190 180 170 160 150 140 130 120 110 100 90 80 70 60 50 40 30 20 10 0 -10
f1 (ppm)

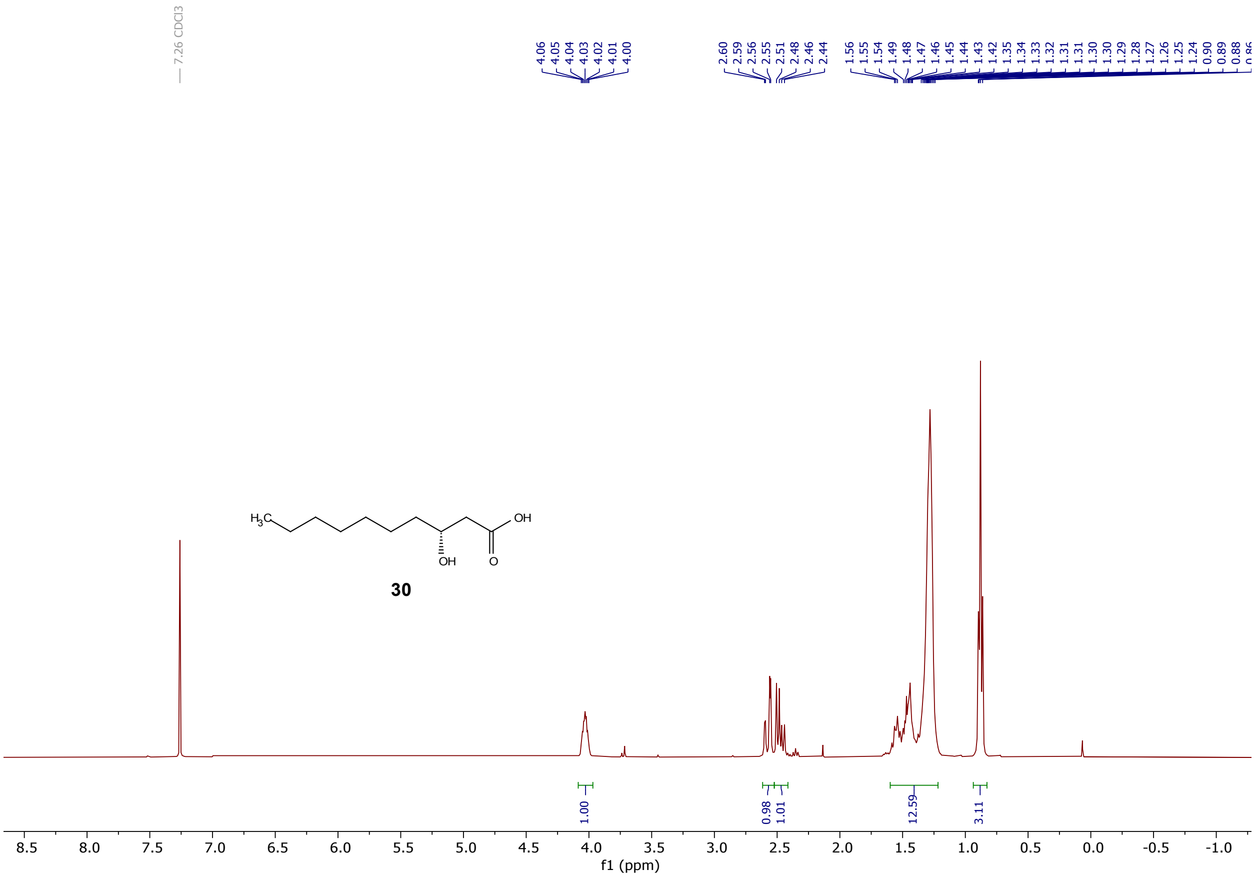
7.26 CDCl3
4.06
4.05
4.04
4.03
4.02
4.01
4.00
2.60
2.59
2.56
2.55
2.51
2.48
2.46
2.44
1.56
1.55
1.54
1.49
1.48
1.47
1.46
1.45
1.44
1.43
1.42
1.35
1.34
1.33
1.32
1.31
1.31
1.30
1.29
1.28
1.27
1.26
1.25
1.24
0.90
0.89
0.88
H3C
OH
OH
O
30
1.00
0.98
1.01
12.59
3.11
8.5
8.0
7.5
7.0
6.5
6.0
5.5
5.0
4.5
4.0
3.5
3.0
2.5
2.0
1.5
1.0
0.5
0.0
-0.5
-1.0
f1 (ppm)

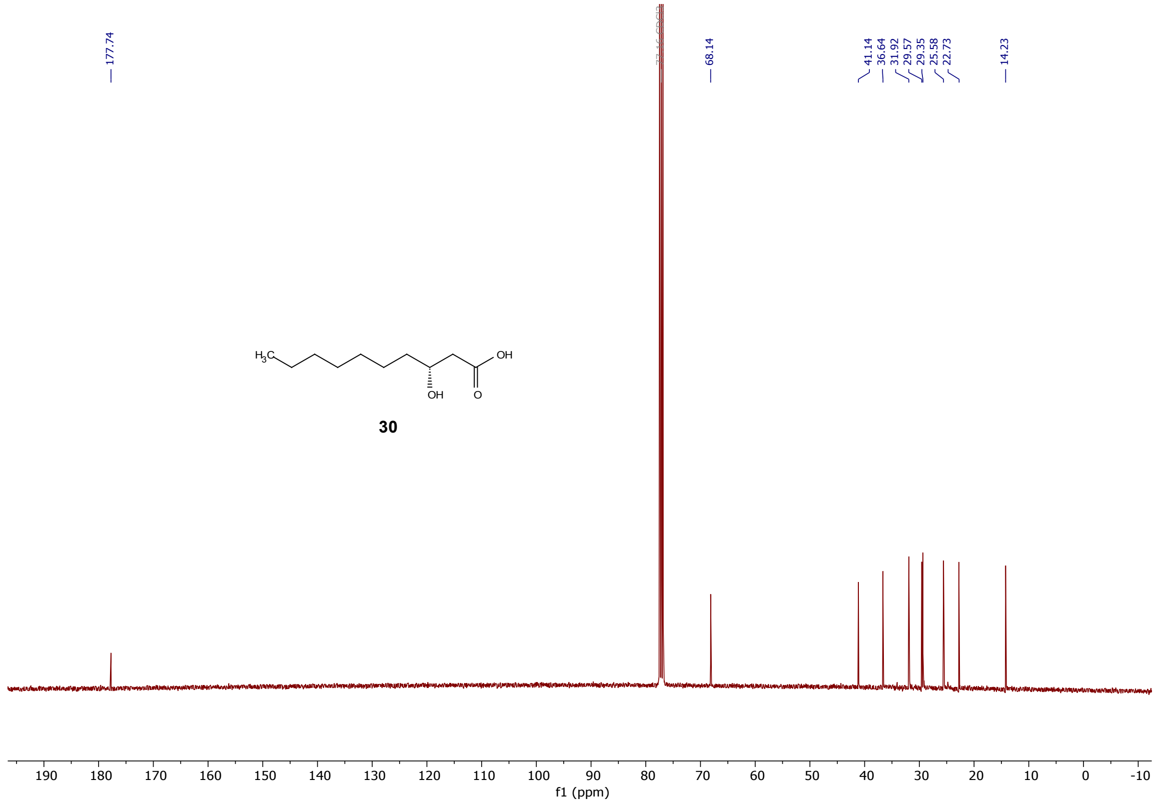
177.74
68.14
41.14
36.64
31.92
29.57
29.35
25.58
22.73
14.23
H3C
OH
OH
O
30
190
180
170
160
150
140
130
120
110
100
90
80
70
60
50
40
30
20
10
0
-10
f1 (ppm)

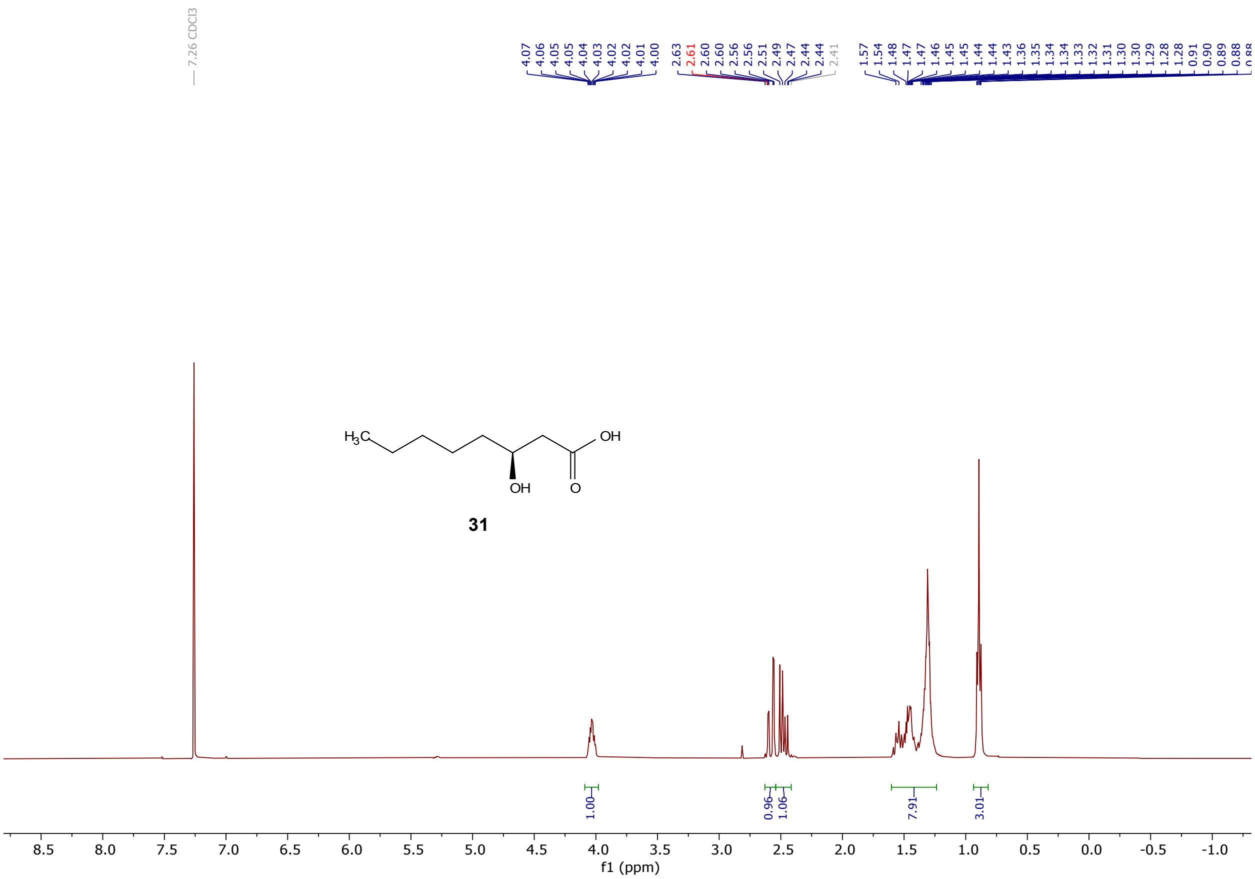
7.26 CDCl3
4.07 4.06 4.05 4.05 4.04 4.03 4.02 4.02 4.01 4.00
2.63 2.61 2.60 2.60 2.56 2.56 2.51 2.49 2.47 2.44 2.44 2.41
1.57 1.54 1.48 1.47 1.47 1.46 1.45 1.45 1.44 1.44 1.43 1.36 1.35 1.34 1.34 1.33 1.32 1.31 1.30 1.30 1.29 1.28 1.28 0.91 0.90 0.89
H3C
OH
OH
O
31
1.00
0.96
1.06
7.91
3.01
8.5 8.0 7.5 7.0 6.5 6.0 5.5 5.0 4.5 4.0 3.5 3.0 2.5 2.0 1.5 1.0 0.5 0.0 -0.5 -1.0
f1 (ppm)

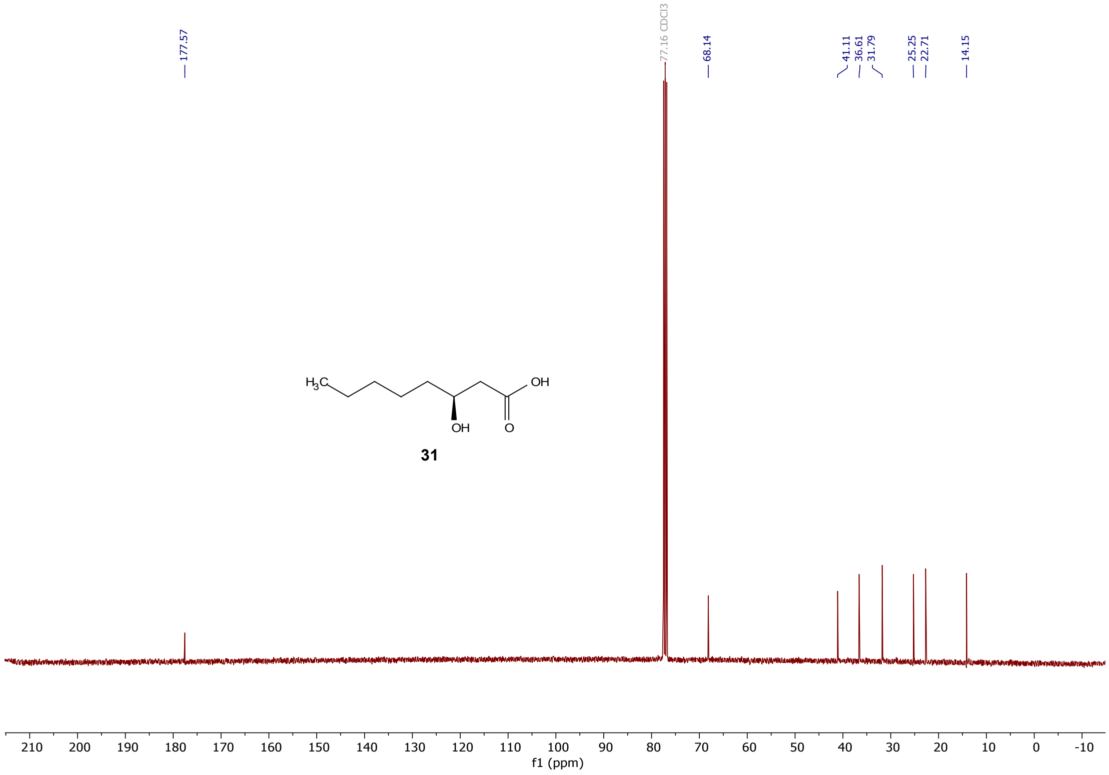
177.57
77.16 CDCl3
68.14
41.11
36.61
31.79
25.25
22.71
14.15
H3C
OH
OH
O
31
210 200 190 180 170 160 150 140 130 120 110 100 90 80 70 60 50 40 30 20 10 0 -10
f1 (ppm)

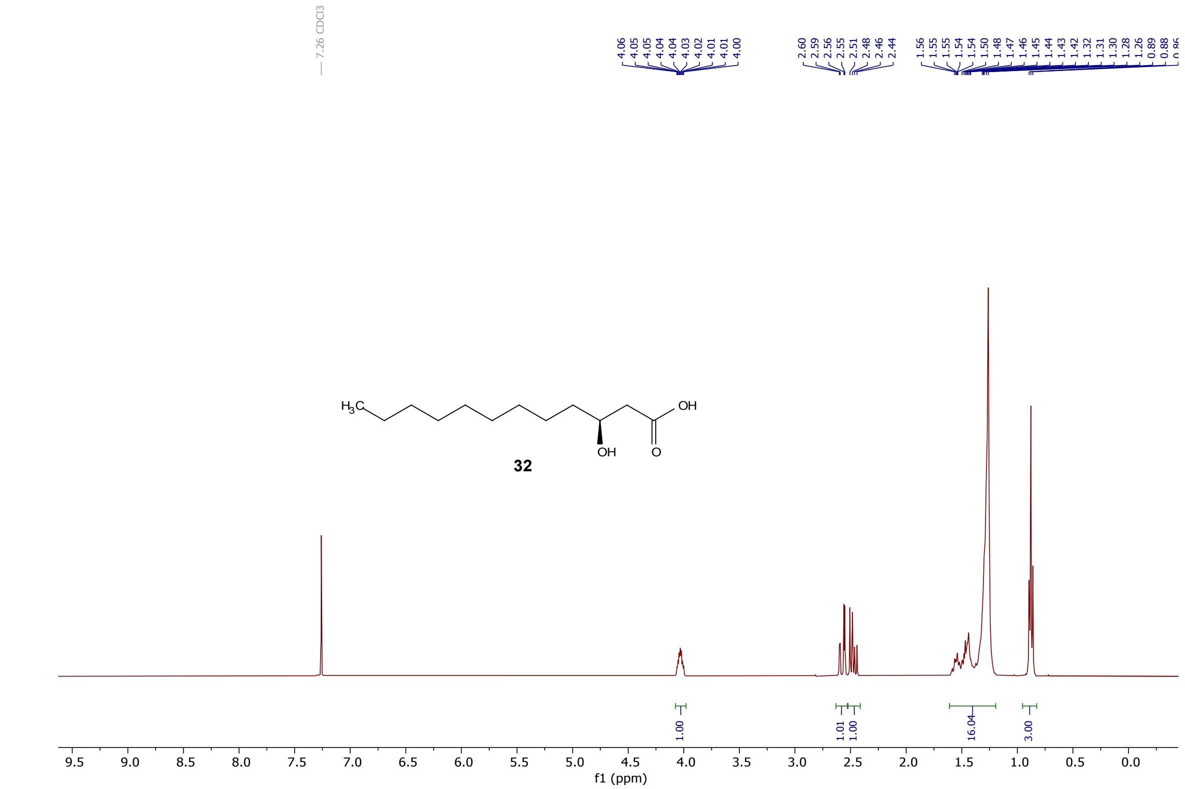
7.26 CDCl3
4.06
4.05
4.05
4.04
4.04
4.03
4.02
4.01
4.01
4.00
2.60
2.59
2.56
2.55
2.51
2.48
2.46
2.44
1.56
1.55
1.55
1.54
1.54
1.50
1.48
1.47
1.46
1.45
1.44
1.43
1.42
1.32
1.31
1.30
1.28
1.26
0.89
0.88
H3C
OH
OH
O
32
1.00
1.01
1.00
16.04
3.00
9.5 9.0 8.5 8.0 7.5 7.0 6.5 6.0 5.5 5.0 4.5 4.0 3.5 3.0 2.5 2.0 1.5 1.0 0.5 0.0
f1 (ppm)

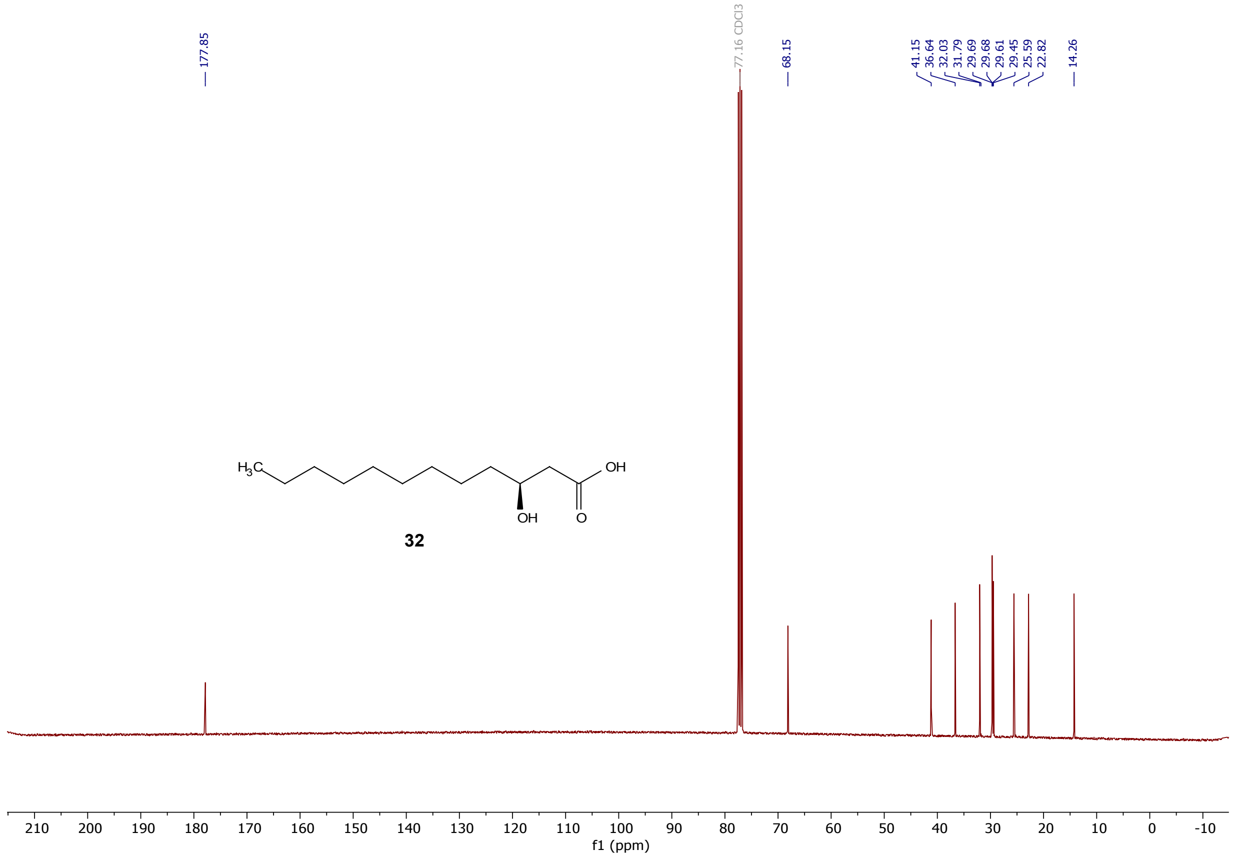
177.85
77.16 CDCl3
68.15
41.15
36.64
32.03
31.79
29.69
29.68
29.61
29.45
25.59
22.82
14.26
H3C
OH
OH
O
32
210 200 190 180 170 160 150 140 130 120 110 100 90 80 70 60 50 40 30 20 10 0 -10
f1 (ppm)

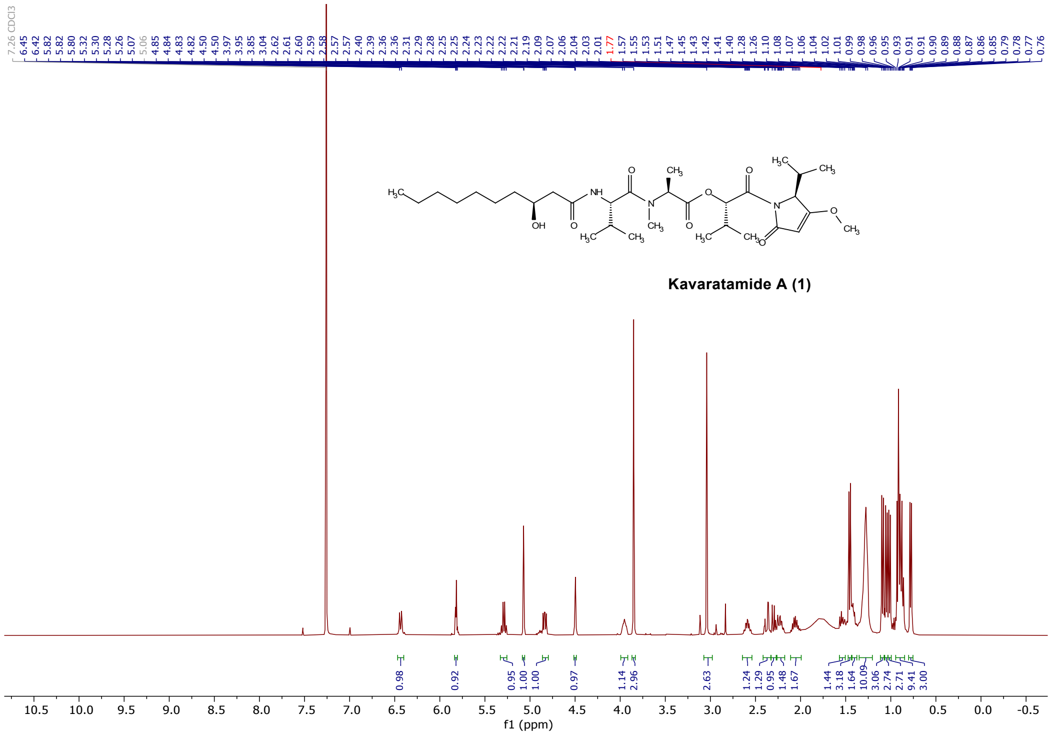
7.26 CDCl3
6.45 6.42 5.82 5.80 5.32 5.30 5.28 5.26 5.07 5.06 4.85 4.84 4.83 4.82 4.50 4.50 3.97 3.95 3.85 3.04 2.62 2.61 2.60 2.59 2.58 2.57 2.57 2.40 2.39 2.36 2.36 2.31 2.29 2.28 2.25 2.25 2.24 2.23 2.22 2.22 2.21 2.19 2.09 2.07 2.06 2.04 2.03 2.01 1.77 1.57 1.55 1.53 1.51 1.47 1.45 1.43 1.42 1.41 1.40 1.28 1.26 1.10 1.08 1.07 1.06 1.04 1.02 1.01 0.99 0.98 0.96 0.95 0.93 0.91 0.91 0.90 0.89 0.88 0.87 0.86 0.85 0.79 0.78 0.77 0.76
Kavaratamide A (1)
0.98 0.92 0.95 1.00 1.00 0.97 1.14 2.96 2.63 1.24 1.29 0.95 1.48 1.67 1.44 3.18 1.64 10.09 3.06 2.74 2.71 9.41 3.00
10.5 10.0 9.5 9.0 8.5 8.0 7.5 7.0 6.5 6.0 5.5 5.0 4.5 4.0 3.5 3.0 2.5 2.0 1.5 1.0 0.5 0.0 -0.5
f1 (ppm)

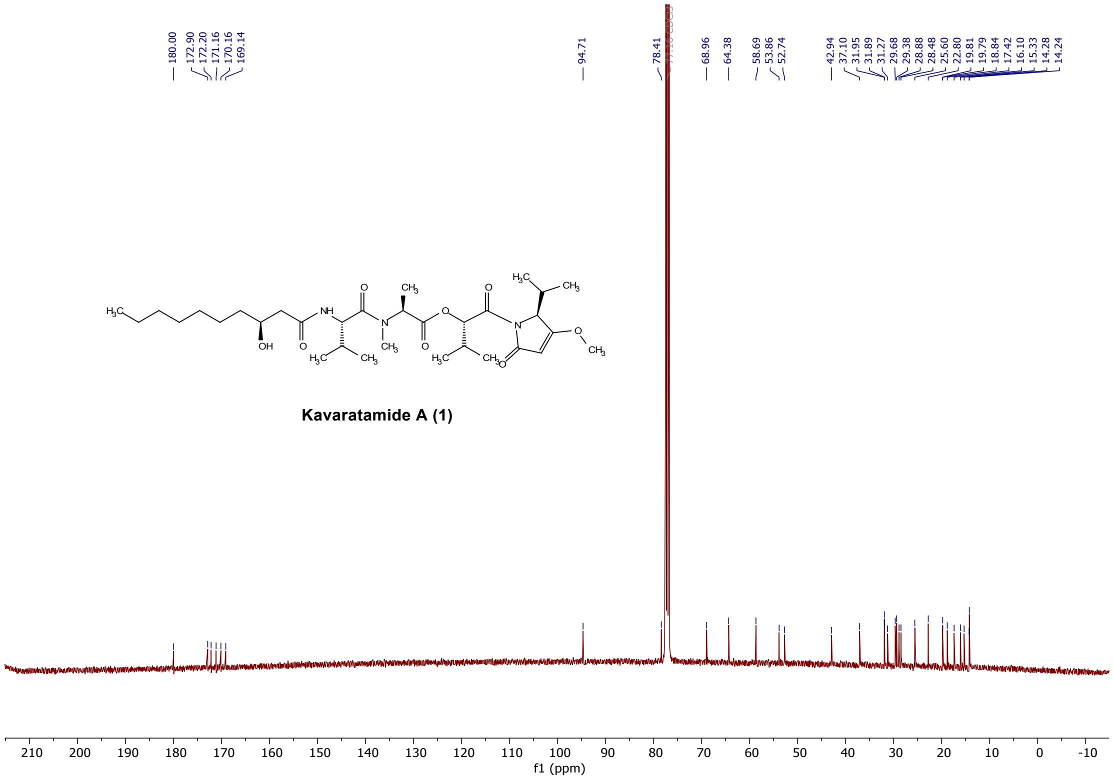
180.00 172.90 172.20 171.16 170.16 169.14
94.71
78.41
68.96 64.38 58.69 53.86 52.74
42.94 37.10 31.95 31.89 31.27 29.68 29.38 28.88 28.48 25.60 22.80 19.81 19.79 18.84 17.42 16.10 15.33 14.28 14.24
Kavaratamide A (1)
210 200 190 180 170 160 150 140 130 120 110 100 90 80 70 60 50 40 30 20 10 0 -10
f1 (ppm)

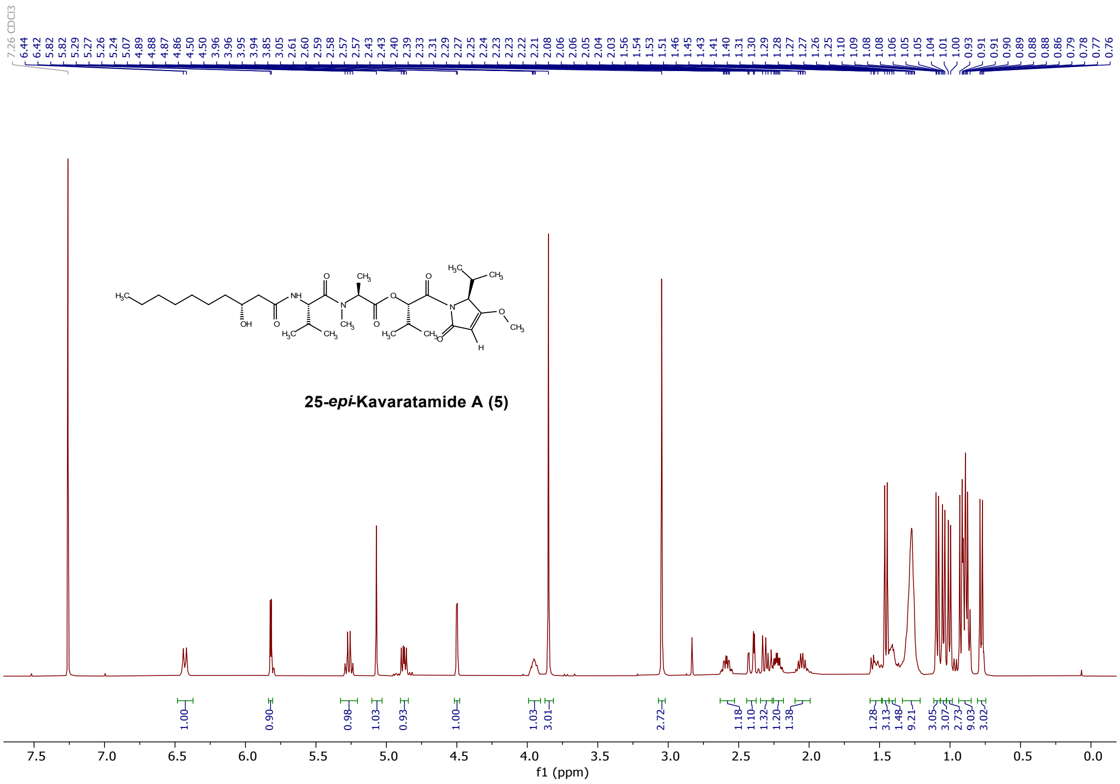
7.26 CDCl3
6.44 6.42 5.82 5.82 5.29 5.27 5.26 5.24 5.07 4.89 4.88 4.87 4.86 4.50 3.96 3.96 3.95 3.94 3.85 3.05 2.61 2.60 2.59 2.58 2.57 2.57 2.43 2.43 2.40 2.39 2.33 2.31 2.29 2.27 2.25 2.24 2.23 2.22 2.22 2.21 2.08 2.06 2.06 2.05 2.04 2.03 1.56 1.54 1.53 1.51 1.46 1.45 1.43 1.41 1.40 1.31 1.30 1.29 1.28 1.27 1.27 1.26 1.25 1.10 1.09 1.08 1.08 1.06 1.05 1.05 1.04 1.01 1.00 0.93 0.91 0.91 0.90 0.89 0.88 0.88 0.86 0.79 0.78 0.77 0.76
1.00 0.90 0.98 1.03 0.93 1.00 1.03 3.01 2.72 1.18 1.10 1.32 1.20 1.38 1.28 3.13 1.48 9.21 3.05 3.07 2.73 9.03 3.02
7.5 7.0 6.5 6.0 5.5 5.0 4.5 4.0 3.5 3.0 2.5 2.0 1.5 1.0 0.5 0.0
f1 (ppm)
25-*epi*-Kavaratamide A (5)

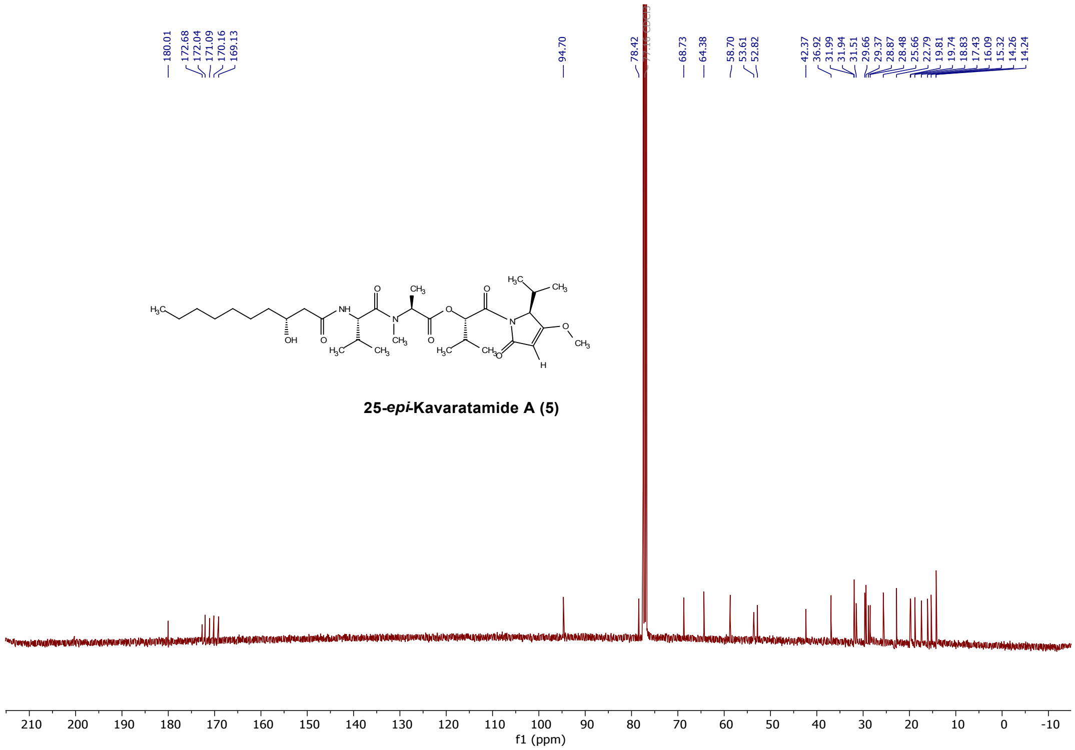
180.01 172.68 172.04 171.09 170.16 169.13 94.70 78.42 77.16 CDCl3 68.73 64.38 58.70 53.61 52.82 42.37 36.92 31.99 31.94 31.51 29.66 29.37 28.87 28.48 25.66 22.79 19.81 19.74 18.83 17.43 16.09 15.32 14.26 14.24
210 200 190 180 170 160 150 140 130 120 110 100 90 80 70 60 50 40 30 20 10 0 -10
f1 (ppm)
25-*epi*-Kavaratamide A (5)

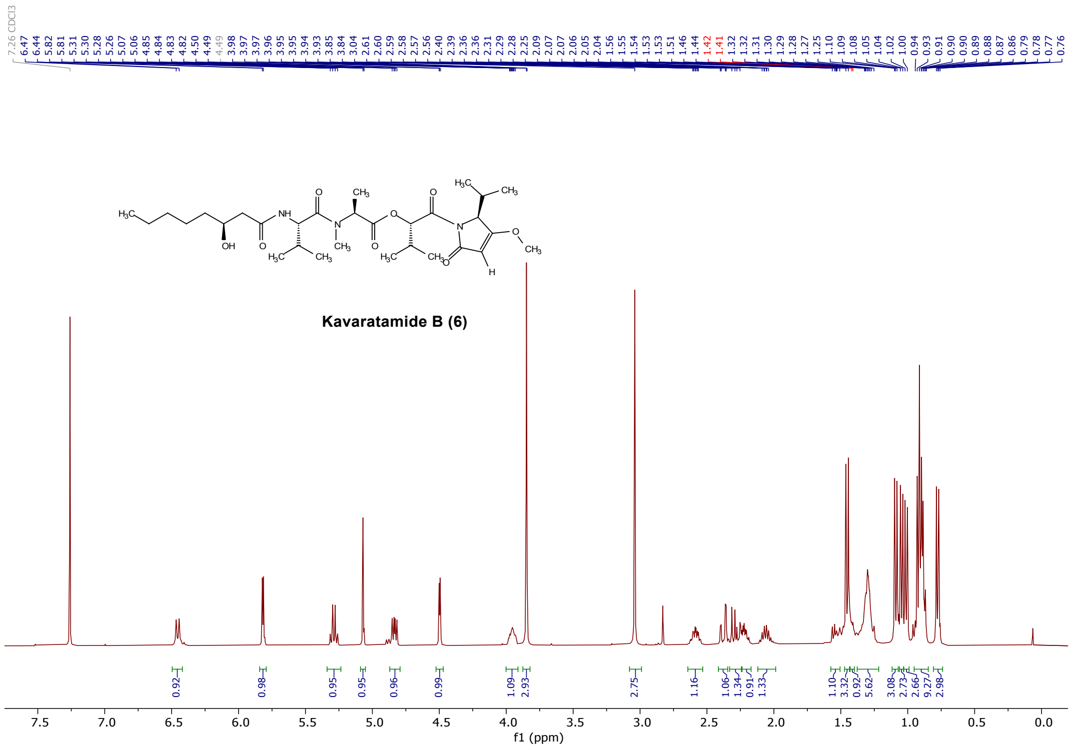
7.26 CDCl3
6.47 6.44 5.82 5.81 5.31 5.30 5.28 5.26 5.07 5.06 4.85 4.84 4.83 4.82 4.50 4.49 4.49 3.98 3.97 3.97 3.96 3.95 3.95 3.94 3.93 3.85 3.84 3.04 2.61 2.60 2.59 2.58 2.57 2.56 2.40 2.39 2.36 2.36 2.31 2.29 2.28 2.25 2.09 2.07 2.07 2.06 2.05 2.04 1.56 1.55 1.54 1.53 1.53 1.51 1.46 1.44 1.42 1.41 1.32 1.32 1.31 1.30 1.29 1.28 1.27 1.25 1.10 1.09 1.08 1.05 1.04 1.02 1.00 0.94 0.93 0.91 0.90 0.90 0.89 0.88 0.87 0.86 0.79 0.78 0.77 0.76
Kavaratamide B (6)
0.92 0.98 0.95 0.95 0.96 0.99 1.09 2.93 2.75 1.16 1.06 1.34 0.91 1.33 1.10 3.32 0.92 5.62 3.08 2.73 2.66 9.27 2.98
f1 (ppm)

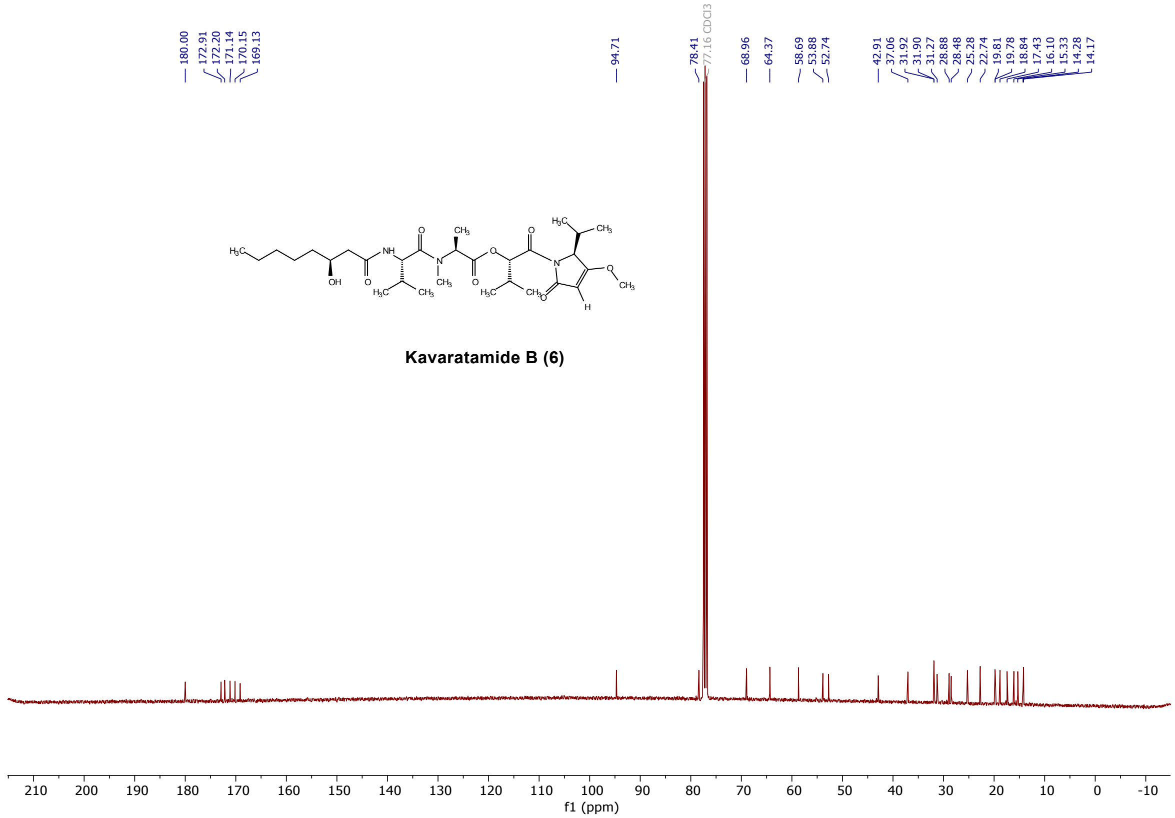
180.00 172.91 172.20 171.14 170.15 169.13 94.71 78.41 77.16 CDCl3 68.96 64.37 58.69 53.88 52.74 42.91 37.06 31.92 31.90 31.27 28.88 28.48 25.28 22.74 19.81 19.78 18.84 17.43 16.10 15.33 14.28 14.17
Kavaratamide B (6)
f1 (ppm)

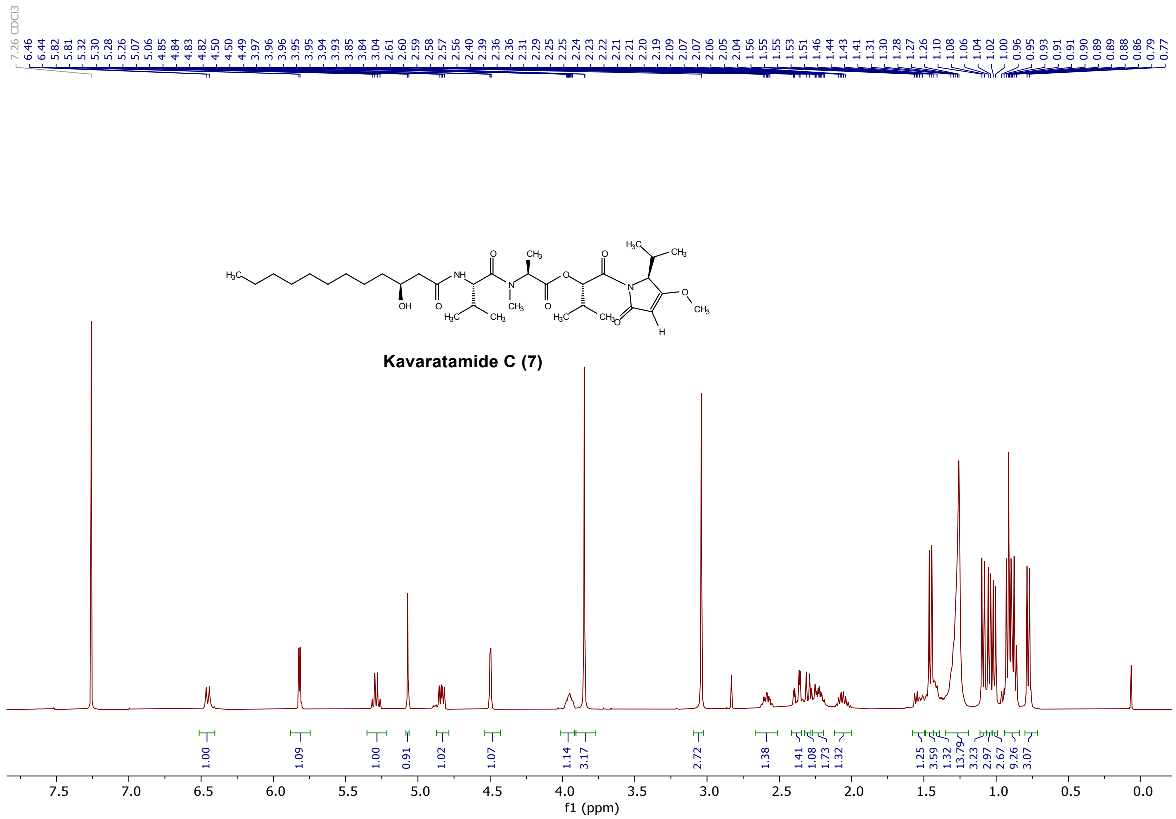

Kavaratamide C (7)

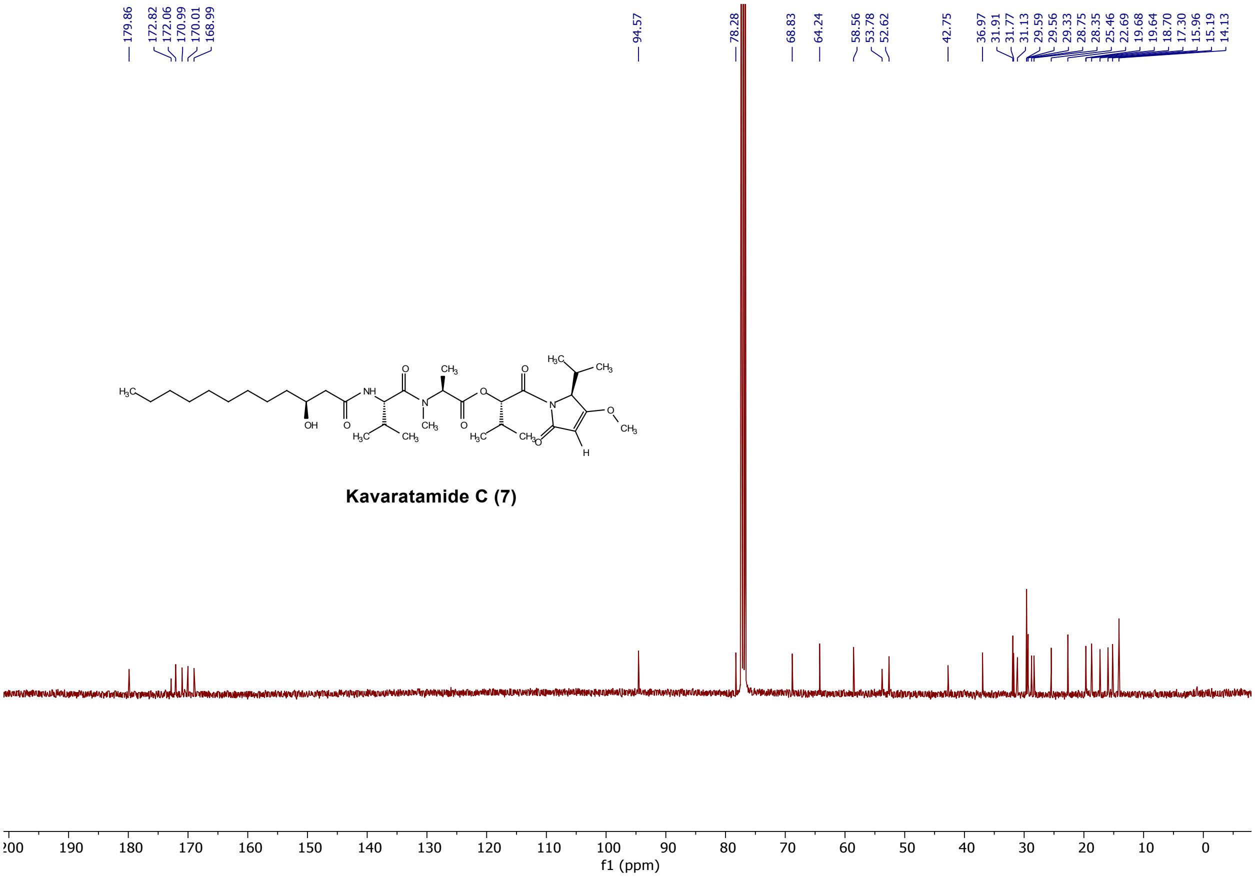

Kavaratamide C (7)

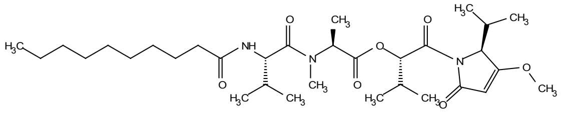

Deoxy-kavaratamide A (8)

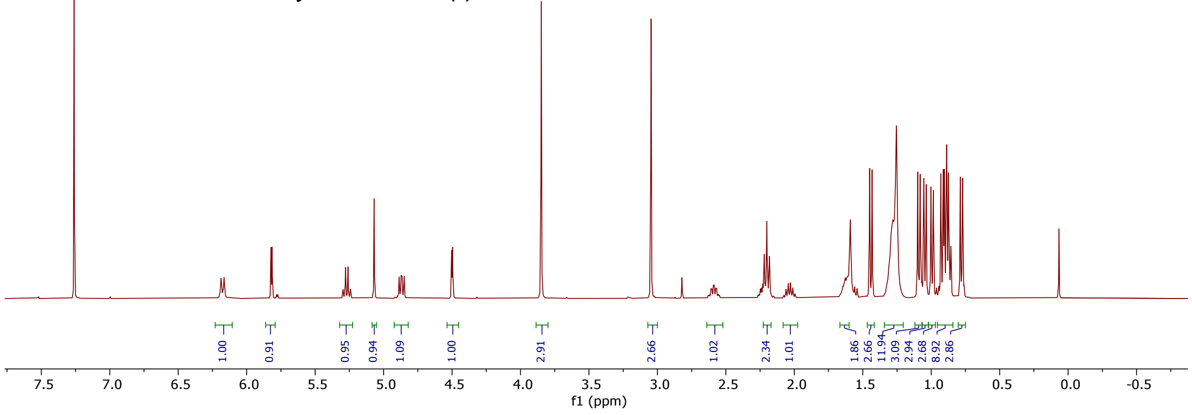

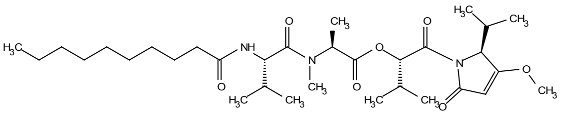

Deoxy-kavaratamide A (8)

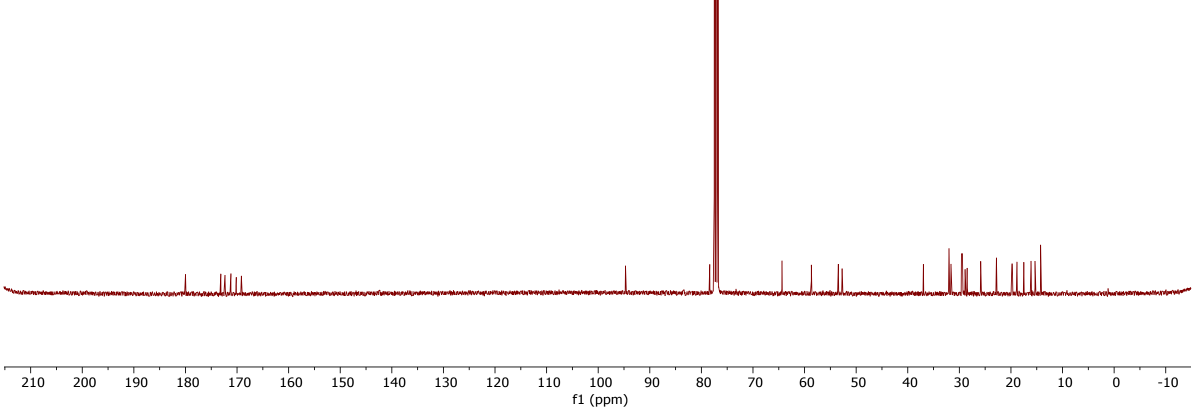

Reference

(1) De Vleeschouwer, M.; Sinnaeve, D.; Van den Begin, J.; Coenye, T.; Martins, J. C.; Madder, A. Rapid Total Synthesis of Cyclic Lipodepsipeptides as a Premise to Investigate Their Self-Assembly and Biological Activity. *Chemistry – A European Journal* **2014**, *20* (25), 7766–7775. https://doi.org/10.1002/chem.201402066.
